# Direct and spillover hospitalisation patterns during climate hazards across regions of different health-system resilience levels in China: a nationwide retrospective analysis

**DOI:** 10.1016/j.lanplh.2026.101512

**Published:** 2026-09-01

**Authors:** Teng Wang, Hanxu Shi, Yujie Shi, Peng Wang, Weiwen Li, Jin Lv, Wenliang Yao, Zhisheng Liang, Mintao Su, Siwen Li, Bin Wang, Murray Ramanathan, Shyam Biswal, Steve Hung-Lam Yim, Ming Xu, Wenjia Cai, Wei Huang, Tiantian Li, Cunrui Huang, Haidong Kan, Minghui Ren, Gordon G Liu, Chung Yee Kwok, Zhenyu Zhang

**Affiliations:** a Department of Global Health, Peking University School of Public Health, Beijing, China; b Department of Architecture and Civil Engineering, School of Engineering, City University of Hong Kong, Hong Kong, China; c Guangdong Provincial Key Laboratory of Durability for Marine Civil Engineering, Shenzhen University, Shenzhen, Guangdong, China; d Department of Radiotherapy, The PLA Rocket Force Characteristic Medical Center, Beijing, China; e Department of Nuclear Medicine, Zibo Central Hospital, Zibo, Shandong, China; f Institute of Reproductive and Child Health, Peking University School of Public Health, Beijing, China; g Bloomberg School of Public Health, Johns Hopkins University, Baltimore, MD, USA; h School of Medicine, Johns Hopkins University, Baltimore, MD, USA; i Centre for Climate Change and Environmental Health (CCEH), Nanyang Technological University, Singapore; j Asian School of the Environment, Nanyang Technological University, Singapore; k Lee Kong China School of Medicine, Nanyang Technological University, Singapore; l Ministry of Education Key Laboratory for Earth System Modeling, Department of Earth System Science, Tsinghua University, Beijing, China; m Institute of Environmental and Health, Peking University School of Public Health, Beijing, China; n National Institute of Environmental Health, Chinese Center for Disease Control and Prevention, Beijing, China; o Vanke School of Public Health, Tsinghua University, Beijing, China; p School of Public Health, Key Lab of Public Health Safety of the Ministry of Education and NHC Key Lab of Health Technology Assessment, Fudan University, Shanghai, China; q China Center for Health Development Studies, Peking University, Beijing, China; r Institute for Global Health and Development, Peking University, Beijing, China; s Institute of Carbon Neutrality, Peking University, Beijing, China

## Abstract

**Background:**

Health-system resilience serves as a key contributor in mitigating adverse health impacts during climate hazards. However, quantitative insights into resilience-associated health-care utilisation patterns and targeted adaptation policies remain scarce. We aimed to capture the spatiotemporal health impacts in disaster-exposed counties and their neighbouring counties in China during storms, floods, tropical cyclones, and blizzards or winter storms; understand the association between health-system resilience metrics and hazard-attributable hospitalisations; and develop evidence-based adaptation policies towards climate extremes.

**Methods:**

In this retrospective, observational analysis of county-level aggregated hospitalisation data, we used a propensity score matching–difference-in-differences framework to assess the spatiotemporal changes of nine types of disease-specific hospitalisations in both disaster-exposed and neighbouring regions during storms, floods, tropical cyclones, and blizzards in China. We quantified the relative importance and health gains of health-system metrics during such hazards through random forest approach with interpretable partial dependence plots to derive evidence-based adaptation recommendations.

**Findings:**

We included hospitalisation data from Jan 1, 2016 to Dec 31, 2023. In this period, 3241 county-hazard event combinations and 41 747 482 hospitalisations were recorded across 955 Chinese counties. The disaster-exposed regions experienced an initial decline in hospitalisation rates, followed by admission surges after disasters. For example, infectious disease admissions decreased by 11·92% (95% CI −10·53 to −13·31) during the flood-active period but increased by 7·68% (6·46–8·91) after 1–2 weeks of floods. Neighbouring zones were also affected through spillover effects, with infectious disease admissions increasing by 3·18% (1·76–4·61) after 1–2 weeks of the floods. Cardiovascular disease, injuries, infectious, respiratory, and mental disorders were more sensitive across all regions. Particularly for disaster-exposed counties, cardiovascular hospitalisations increased by 14·31% (7·34–21·29) during the tropical cyclone-active period. Notably, compared with low-resilience counties, high-resilience counties were associated with 19·48–30·03% smaller hazard-related relative changes in hospitalisation rates during the hazard-active period and 27·07–31·08% smaller hazard-related relative changes in hospitalisation rates in post-hazard periods. For instance, during the storm-active period, the increase in respiratory hospitalisations was 7·21% (0·67–13·75) in high-resilience counties versus 12·13% (5·20–19·05) in low-resilience counties. Health workforce (relative importance 14·58% during the hazard-active period and 13·80% during the post-hazard period) and service delivery (14·10% during the hazard-active period and 14·17% during the post-hazard period) were identified as key contributors of health-system resilience. Empirical synergistic effects were observed when combining interventions during the post-hazard period, with the combined effect of service delivery (individual contribution 8%) and workforce (individual contribution 4%) exceeding the sum of their individual contributions (16% reduction in cumulative excess admissions) by 33%.

**Interpretation:**

Climate hazards are associated with substantial changes in hospitalisation rates in both disaster-exposed and neighbouring regions. Health-system resilience is essential in addressing disaster–health challenges. Targeted adaptation interventions should be context-appropriate and threshold-aware, thereby maximising the public health benefits relative to resilience-oriented investments in health systems.

**Funding:**

Gates Foundation and the National Natural Science Foundation of China.

## Introduction

Climate hazards pose unprecedented challenges to health-care systems worldwide, necessitating urgent attention to disaster-related health consequences and evidence-based adaptation strategies.^1^ During disasters, health-care systems in neighbouring non-exposed regions display complex patterns of collaboration and adaptation,^2^ sharing excess health-care burden through environmental dispersion and patient migration. Environmental dispersion refers to the spread of harmful conditions from disaster epicentres to adjacent areas, directly threatening public health in neighbouring counties. For example, smoke plumes and elevated particulate matter (PM)_2.5_ concentrations can extend beyond the immediate vicinity of wildfires,^3^ and heavy rainfall can affect areas far from the centre of floods.^4^ Patient migration represents the movement of individuals seeking medical care outside disaster zones,^5^ substantially redistributing health-care demand. Measuring these spillover effects is essential for fully assessing hazard effects and guiding targeted preparedness, resource allocation, and risk management^6^ in disaster-exposed and neighbouring areas.

**Research in context**

**Evidence before this study**

Health-system resilience is conceptually recognised as an essential determinant in mitigating adverse health impacts during climate hazards. However, quantitative insights into spatiotemporal disaster–health impacts, resilience-associated health outcomes, and targeted adaptation policies remain scarce. Before undertaking this study, we searched PubMed and Web of Science for papers published from database inception to Oct 31, 2024 using the terms (“climate hazards” OR “extreme weather disasters” OR “storm” OR “flood” OR “tropical cyclone” OR “blizzard” OR “winter storm”) AND (“health impacts” OR “hospitalizations” OR “hospitalization rates” OR “spillover”) AND (“health system resilience” OR “health system disparities” OR “adaptation policy”). Our search was restricted to articles published in English. Studies were excluded if they were conference abstracts or review articles without original data. We found that most studies focused on localised settings or a single health outcome and often used time-series methods, thereby failing to capture the broader health consequences, spillover pathways, or targeted adaptation policies associated with climate hazards. Although review papers increasingly recognised the role of resilience guidelines in addressing climate hazard challenges, no previous studies offered quantitative insights into real-world practice by assessing the relative importance of health-system components, the explicit public health gains from health-system interventions, or the potential investment thresholds beyond which health returns diminish across diverse contexts.

**Added value of this study**

Our study advances the evidence base on climate hazard-related hospitalisation patterns in several ways. First, we comprehensively investigated the spatiotemporal health impacts across all hazard phases in both disaster-exposed and neighbouring regions. Second, by explicitly quantifying the spillover pathways associated with environmental dispersion and patient migration, our analysis showed complex crossregional patterns of health-care utilisation. Third, we introduced a framework that integrated static resilience metrics with observed hospitalisation patterns associated with climate hazards, enabling examination of associations between baseline and hazard-related differences in hospitalisation burden across regions. To the best of our knowledge, this study presents the first multiple-hazard–health impact analysis at the national scale. Our findings established empirical evidence linking health-system characteristics to differential hospitalisation patterns during hazard-active and post-hazard periods, based on which we identified the most influential health-system components. Moreover, partial dependence plots enabled us to quantify the potential health gains associated with resilience-oriented interventions and to identify investment thresholds relevant to health-system resilience, thereby providing policy makers with context-specific guidance for resource allocation. As climate extremes intensify globally, our findings provide timely, actionable evidence for policy makers, public health professionals, and climate adaptation specialists seeking to build adaptive, equitable, and resilient health systems in both developed and developing contexts.

**Implications of all the available evidence**

Synthesising conceptual guidelines with our new evidence highlights the urgent need for targeted, resilience-oriented adaptation policies in disaster risk management. Our findings indicate that although climate hazards impose substantial direct and spillover health-care burdens across regions, regions with high health-system resilience experience approximately 30% smaller increases in hospitalisations during hazards. For effective local resilience enhancement, our data-driven approach offers the following targeted adaptation recommendations: low-resilience regions should prioritise fundamental investments in the health workforce, financing, and service accessibility, whereas high-resilience regions would benefit from multisectoral strategies that maximise health returns during hazards through synergistic effects. Moreover, elucidation of the spillover impacts and underlying pathways underscore the necessity for policy makers to implement pre-disaster coordination protocols and develop targeted resource allocation frameworks that optimise health-care system utilisation among disaster-exposed and neighbouring counties. By advancing an integrated, resilience-oriented approach to disaster–health management, our work contributes to the broader agenda of achieving universal health coverage and health equity as articulated by WHO. Future research should prioritise longitudinal and multisectoral approaches, leveraging more comprehensive data sources (eg, community health surveys and targeted sampling in underserved areas) and cross-disciplinary collaborations to better understand the pathways through which climate hazards affect health and to inform the design of adaptive, equitable, and sustainable health-care systems worldwide.

Cause-specific hospitalisations and mortality in disaster-exposed regions^7^^,^^8^ and spatial spillover effects, including the health impacts of air pollution^9^ and infectious disease transmission,^10^ have been reported previously. However, systematic quantification of climate hazard-induced spillovers in health-care demand across exposed and neighbouring regions remains insufficient, particularly in the health-system resilience contexts.

Health-system resilience, defined as the capacity to prepare for, absorb, and recover from adverse events while maintaining essential functions, has emerged as a key contributor for hazard mitigation.^1^^,^^6^^,^^11^ However, current resilience assessments predominantly rely on static composite indexes^12^^,^^13^ that describe the inherent characteristics of regional health systems and hence cannot capture how health-care utilisation patterns vary across disaster phases (eg, hazard-active and post-hazard periods) and between regions with different resilience levels.^6^^,^^11^^,^^14^ This methodological limitation hinders the translation of conceptual insights into actionable policy measures. Although regions with robust health systems are more prepared and adaptable than regions without such systems,^15^ statistical evidence linking health-system resilience to improved health outcomes during disasters remains scarce.^1^ Therefore, we propose a comprehensive assessment framework that combines static resilience metrics with observed health-care utilisation patterns during and after disasters to collect data that can inform resilience interventions.

Furthermore, previous studies primarily addressed policy implications by differences in health risks among clinically vulnerable populations (eg, children and older people^3^) or across regions with different levels of economic prosperity.^16^ Although these approaches were informative, they overlooked the complex relationship between resilience metrics and detailed health outcomes, thus restricting their quantitative insights for policy making. For example, priority health-care components or thresholds beyond which further improvements in resilience yield diminishing marginal health benefits following hazards have not been identified previously. To address this methodological gap, we integrated resilience metrics with disaster-related health impacts using an interpretable machine learning approach, to support policy makers in developing more targeted adaptation strategies that account for regional contexts and resource allocation thresholds.

Specifically, we used a propensity score matching (PSM)–difference-in-differences (DID) method to evaluate resilience-stratified health-care utilisation patterns, spillover effects, and redistribution of the hospitalisation burden across disaster-exposed and neighbouring regions in China during storms, floods, tropical cyclones, and blizzards or winter storms between 2016 and 2023. Using daily hospitalisation data across nine major disease categories from the Chinese multiple counties (CMC) Hospital Network, we aimed to identify the spatiotemporal patterns of hazard-related hospitalisation changes and spillover pathways in disaster-exposed and neighbouring regions; develop a comprehensive health-system resilience assessment framework that integrates static resilience metrics with observed health-care utilisation patterns during hazards; and provide evidence-based policy recommendations to optimise resilience-oriented interventions across diverse regions by prioritising underdeveloped regions vulnerable to climate hazards.

## Methods

### Data collection and procedures

In this retrospective, observational study, we collected daily cause-specific hospitalisation data from the CMC Hospital Network between Jan 1, 2016 and Dec 31, 2023. Climate hazard data were obtained from the emergency events database (EM-DAT). The local Köppen–Geiger climate classifications were obtained from the GloH2O database.^17^ Statistical data for health-system resilience assessment and confounding variables for the PSM–DID analysis were obtained from the National Healthcare System Administration and the National Bureau of Statistics.

We used county-level aggregated hospitalisation data from the CMC Hospital Network, integrated with multiple climate hazard records and health-system-related statistical data, to examine associations between climate hazard exposure and cause-specific hospitalisation patterns across regions with different levels of health-system resilience. To characterise the spatiotemporal health impacts of disasters and inform evidence-based adaptation interventions, we first developed a comprehensive framework for assessing health-system resilience across all administrative regions in China. We then used a PSM–DID approach to quantify the effects of hazards on cause-specific hospitalisations in disaster-exposed and neighbouring regions during hazard-active and post-hazard periods. Next, we compared health outcomes between regions with high and low resilience and used a random forest approach with partial dependence plots to identify the key contributors, effects, and potential thresholds of health-system components for hazard mitigation through resilience-oriented interventions. Finally, we performed sensitivity analyses to assess the robustness of the primary findings and policy implications.

This study used aggregated public health data without personally identifiable information. The committee of Peking University waived the requirement for written informed consent.

### Health-system resilience metrics

Following the WHO conceptional guidelines^18^ and an extensive literature review, we developed a comprehensive framework to assess health-system resilience, including 39 indicators across nine pillars: service delivery, health workforce, preventive interventions, longevity, financing, physical health, mental health, maternal and child health, and infectious disease (appendix pp 39–40). For each region, we aggregated resilience indicators at the pillar level and compiled them into a composite resilience score. Counties ranked in the top half were classified as high-resilience regions, and the remainder as low-resilience regions. Details of data preparation, aggregation, and sensitivity analyses are provided in the appendix (pp 4–7).

### Climate hazard exposure and observation windows

We analysed 20 storms, 58 floods, 29 tropical cyclones, and four blizzards or winter storms in China recorded in the EM-DAT between Jan 1, 2016 and Dec 31, 2023. In this study, storms included convective storms (four general storms, five severe weather events, three tornadoes, and eight lightning–thunderstorms), as defined by the 2014 IRDR Peril Classification and Hazard Glossary.^19^ Tropical cyclones and blizzards or winter storms were analysed as separate categories following established conventions in environmental health research.^7^ These disasters were documented at the county level, and EM-DAT entry criteria required at least one of the following conditions met—namely, ten or more fatalities, 100 or more individuals affected, a declaration of emergency, or a call for international assistance.^20^ We crossvalidated the hazard distribution against the geocoded disasters database^21^ to ensure that no substantial temporal, threshold, or geographical bias was present.^22^ To address potential bias from co-occurring or cascading hazards (eg, flooding induced by tropical cyclones), we excluded counties that experienced more than one hazard simultaneously or had an interval of less than 3 months between disasters (appendix pp 7–8).

We set sequential observation windows spanning the complete hazard-effective phases. For each event, we assumed that the hazard lasted for $T_{e}$ days with its onset as day 1; the observation windows before and after the hazard for comparison using the DID framework were defined as follows: warning (13 days to 7 days before the event [the baseline reference representing health-care utilisation unaffected by any hazard-related information] *vs* 6 days to 0 days before the event [the period immediately preceding hazard onset, during which early warning effects on health-care seeking behaviour might already be apparent]); enduring (6+$T_{e}$ days to 7 days before the event *vs* day 1 to $T_{E}$); stabilisation 1 (20 days to 7 days before the event *vs* $T_{E}$+1 to $T_{E}$+14 days after the event); stabilisation 2 (20 days to 7 days before the event *vs* $T_{E}$+15 to $T_{E}$+28 days after the event); and return to normalcy (20 days to 7 days before the event *vs* $T_{E}$+29 to $T_{E}$+42 days after the event).

Event-specific recovery trajectories can vary across hazard types and settings. However, we chose a standard 2-week observation window to balance three considerations: sufficient sample size for reliable DID estimation,^7^ alignment with health-care system operational cycles,^23^ and crosshazard comparability. The empirical results substantiated this approach by showing consistent temporal patterns across all hazard types, with peak impacts observed during weeks 1–2 and progressive recovery thereafter. Further details of hazard entry criteria,^20^ crossvalidation, and observation window settings are in the appendix (pp 7–8).

### Health outcomes

We obtained daily cause-specific hospitalisation data from the national inpatient medical record dataset documented by the CMC Hospital Network, which covers 955 counties and represents 44·73% of the national population. Although the CMC Hospital Network covers fewer counties in western China (24·98%) than in other parts of China, the covered counties represent 68·95% of western China’s population and closely reflect the national population density pattern, indicating minimal population-weighted geographical bias (appendix pp 18–19).

We defined disease categories according to the International Classification of Diseases-10 codes.^24^ To calculate disease-specific hospitalisation rates, population denominators were derived from 1-km gridded population data^25^ within an empirically calibrated 16-km service radius of each hospital. Details of the CMC Hospital Network, patient information, and disease coding are provided in the appendix (pp 8–9 and 41).

### County classification

We systematically classified all counties as disaster-exposed, neighbouring, or control counties based on geographical proximity to disaster-affected areas. Disaster-exposed counties were directly affected, whereas neighbouring counties were further stratified into N-1 (0–100 km), N-2 (100–200 km), and N-3 (200–300 km) bands based on the average distance to hospital, the outline of the national medical and health service system plan (2015–20) guideline,^26^ and geographical thresholds for health impacts across China and in the densely populated eastern and sparsely populated western regions.^27^ We defined candidate control counties as those located more than 300 km from disaster-affected areas because DID estimates suggested that spillover effects had attenuated to non-significant levels within the 200–300 km band across regions. Further details on county classification criteria and spatial thresholds justification are available in the appendix (pp 9–10).

We identified a county’s designation as treated (hazard-affected) or control (non-affected) separately for each hazard event based on its spatial and temporal relationship to that event. For example, a county exposed to a flood in 1 year could serve as a matched control for a geographically and temporally distinct flood event in another year, provided it satisfied all matching criteria (eg, same climate type, same resilience level, balanced covariates, and no spatial autocorrelation) and had no concurrent or recent hazard exposure within the last 3 months. We selected this 3-month requirement for control county qualification as hospitalisation rates did not differ notably beyond 3 months after hazard exposure; similar criteria have been used in previous studies.^7^^,^^28^ This event-specific design maximised the pool of eligible control counties while preserving the validity of the DID estimates by ensuring that the treated and control counties showed no differential trends in hospitalisation patterns before hazard exposure.

### Spillover pathways

We distinguished two main spillover pathways contributing to excess hospitalisations in neighbouring regions. One was environmental dispersion, measured by increased hospitalisation rates among local residents; the second was patient migration, quantified by hospital admissions of non-local individuals from disaster-exposed areas. Moreover, to clarify the role of health-system resilience in modulating these spillover pathways, we performed analyses stratified by admission source (emergency department, outpatient, and interinstitutional transfer). Details of the stratification analysis are in the appendix (p 10).

### Statistical analysis

We used the PSM–DID framework to estimate associations between climate hazards and changes in cause-specific hospitalisation rates. This approach enabled the estimation of hazard effects while accounting for observable confounders and unobservable time-invariant heterogeneity across counties.

We applied a multistep matching protocol to ensure comparability between hazard-affected counties and unaffected controls. First, we assessed spatial autocorrelation in cause-specific hospitalisation rates among candidate control counties using global Moran’s *I*. If significant spatial autocorrelation was detected (p<0·05; 999-permutation test), we computed local Moran’s *I* for each candidate county.^29^ Counties with significant positive local spatial autocorrelation (p<0·05, conditional permutation test) were excluded from the control pool before PSM to preserve the independence assumption underlying DID inference. We retained only those counties that shared the same climate type^17^ (appendix p 20) and health-system resilience level. After pre-selection, we used 1:4 nearest-neighbour PSM to balance county-level covariates across multiple domains, including demographic (population density, proportion of older people [aged 65 years or more], educational attainment, and sex ratio), socioeconomic (gross regional product [GRP] per capita and GRP growth rate), environmental (green coverage and urbanisation rate), and health system (service delivery and expenditure) domains (appendix pp 21–22).

After matching, we validated the parallel trends assumption using an event–study approach (appendix pp 23–26).^30^^,^^31^ Across all hazard–disease combinations, prehazard coefficients were consistently centred around zero, with 95% CIs overlapping zero, indicating no significant differential trends before hazard exposure. In contrast, post-hazard coefficients differed significantly from zero, indicating that treatment effects emerged only after hazard exposure. We found no material violations of the parallel trends assumption in any hazard–disease panel. This systematic validation supported the parallel trends assumption held for all analyses, establishing the necessary foundation for reliable DID inference. After validation, we used generalised linear models with county and time-fixed effects to capture the DID effects across all observation periods in both disaster-exposed and neighbouring counties for all categories of climate hazards. Details for matching procedure, PSM confounding factors, covariate balance verification results, and DID model specification are provided in the appendix (pp 10–13).

Using the DID estimates, we quantified differences in hospitalisation risk between high-resilience and low-resilience regions during hazard-active and post-hazard periods (using the excess relative risk [ERR=RR−1], with RR being the relative risk calculated by the PSM–DID analysis). Details of the calculation are provided in appendix p 12.

For adaptation policy insights, we used a random forest approach to estimate the relative importance^32^ of resilience metrics in reducing excess hospitalisations during hazards. We then constructed partial dependence plots^33^ to visualise the individual and combined health benefits and identify potential thresholds from resilience-oriented interventions. Finally, we constructed accumulated local effect^34^ plots to crossvalidate the policy recommendations. Details on random forest modelling, partial dependence plots, and accumulated local effects are available in the appendix (pp 15–17).

We performed a series of sensitivity analyses to assess the robustness and reliability of the primary findings. First, we used a Monte Carlo approach^13^ and a principal component analysis-based data-driven approach to evaluate the sensitivity of the resilience assessment framework to alternative weighting schemes. Second, we conducted complementary tertile-based resilience classifications (low, moderate, and high) to examine resilience gradient effects and assess the robustness of the policy recommendations. Third, we examined the robustness of disaster–health impact modelling by re-estimating the DID models using mixed-effects specifications with county-level random effects. Fourth, we repeated DID analyses without previous PSM to examine potential selection bias^35^^,^^36^ that might arise from selection controls with atypically similar baseline values or from less utilised control counties in western China. Fifth, we repeated the PSM–DID analysis by excluding the pandemic period (Jan 1, 2020 to Dec 31, 2022). Finally, we generated accumulated local effect plots to crossvalidate policy insights (appendix pp 10–13).

All statistical analyses were performed using R version 4.4.1, MATLAB R2023a, and ArcGIS Pro 3.2. Statistical significance was defined as p<0·05, and 95% CIs were calculated for all effect estimates.

### Role of the funding source

The funders of this study had no role in study design, data collection, data analysis, data interpretation, or writing of the report.

## Results

All reported percentage changes represent PSM–DID-estimated excess relative risks (ERR = RR − 1), reflecting the hazard-related relative change in hospitalisation rates in hazard-affected counties compared with matched control counties.

From 2016 to 2023, 3241 county-hazard event combinations (521 storms, 1959 floods, 324 tropical cyclones, and 437 blizzards or winter storms) were recorded across 955 Chinese counties, indirectly threatening 5417 neighbouring counties; 436 (8·05%) of these counties belonged to the N-1 band, 1787 (32·99%) belonged to the N-2, and 3194 (58·96%) belonged to the N-3 band (figure 1A, appendix pp 27 and 42).

**Figure 1:**
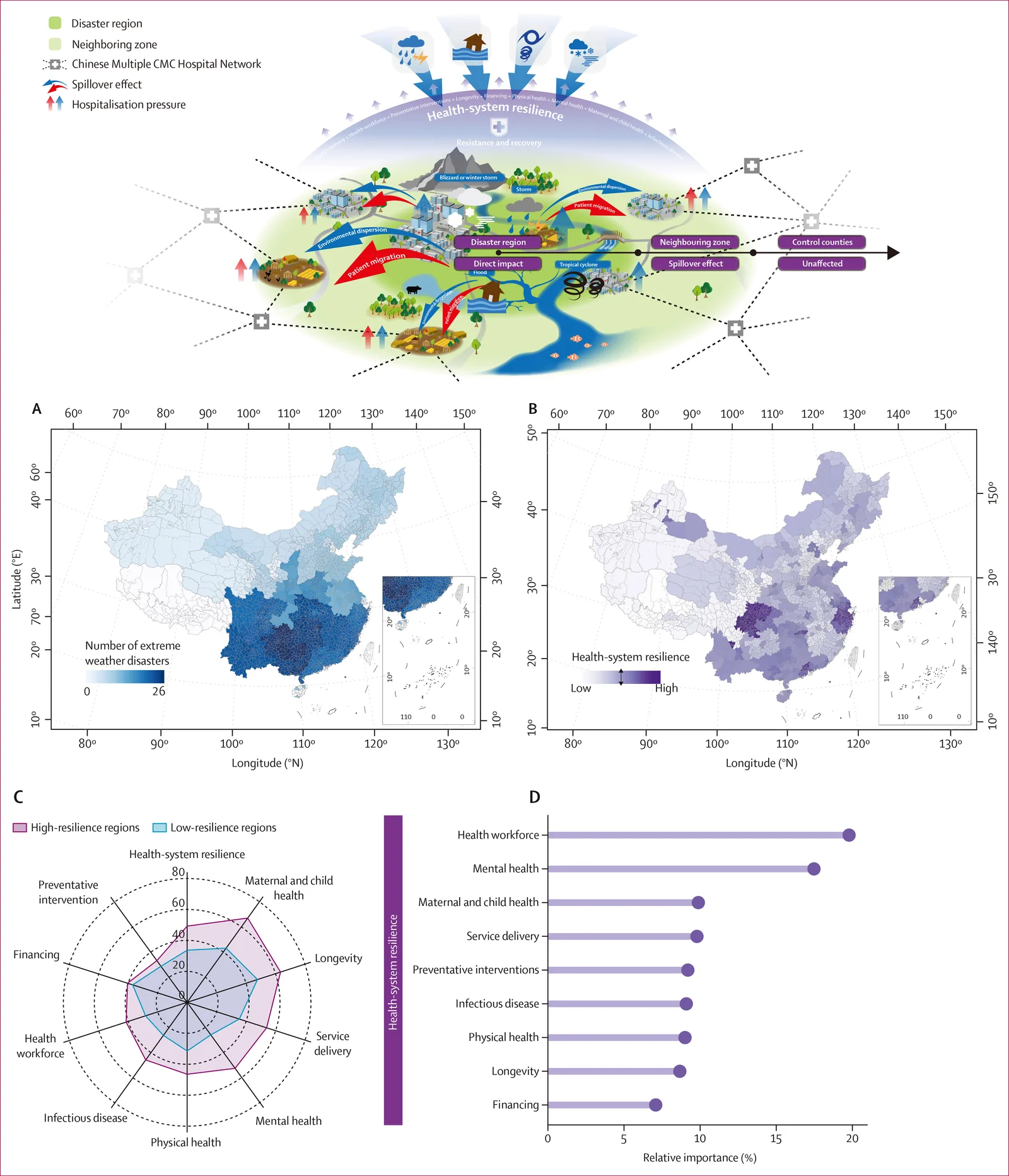
Climate hazard exposure characteristics and health-system resilience in China The schematic diagram above the panels provides a conceptual overview illustrating that multiple climate hazards (storms, floods, tropical cyclones, and blizzards or winter storms) are associated with substantial direct health impacts in disaster-exposed regions (dark green area) and indirect spillover health impacts in neighbouring regions (light green area), while health-system resilience might confer protective effects by mitigating hazard-related changes in hospitalisation rates. (A) Distribution of overall climate hazards (including storms, floods, tropical cyclones, and blizzard or winter storms) from 2016 to 2023. (B) Distribution of high-resilience and low-resilience regions in China, coloured by the overall health-system resilience marks assessed by the comprehensive evaluation framework. (C) Comparison between mean values of resilience pillars in low-resilience and high-resilience regions. (D) Relative importance of resilience metrics on overall resilience marks, quantified by the random forest approach. CMC=Chinese multiple counties.

The CMC Hospital Network (1670 hospitals) registered 41 747 482 hospitalisations, most of which were for cardiovascular diseases (6 245 250 [14·96%]), respiratory diseases (6 078 654 [14·56%]), genitourinary diseases (5 330 752 [12·77%]), and injuries (3 033 260 [7·27%]). Individuals 45 years or older constituted the most hospitalisations (25 957 923 [62·17%]), and admissions were primarily from the emergency department (30 352 545 [72·71%]; appendix p 41).

In general, the eastern regions had higher levels of health-system resilience than the underdeveloped western regions in China (figure 1B). High-resilience regions showed superior performance across all resilience metrics, especially for county-level health-care infrastructure and individual health indicators, including mental, physical, and maternal–child health (figure 1C, appendix pp 28–29). Moreover, we identified health workforce (relative importance derived from the random forest algorithms: 18·51%; see Methods) and residents’ mental health (15·67%) as the most crucial pillars in static resilience assessment (figure 1D). Other pillars, such as maternal or child health services (11·10%) and preventive interventions (10·24%) were less important. Health-care financing, such as government financial allocation (1·23%), general primary health-care expenditure (1·32%), insurance (3·19%), and price for medicine products (1·70%), showed low variation across regions because of government allocation and standardised policies (appendix p 30).

Climate hazards were associated with substantial changes in hospitalisation rates in disaster-exposed regions, with spillover patterns progressively attenuated across the neighbouring zones (figure 2, appendix pp 43–46). The spatial spillover extent was restricted to 200 km, as no significant increase in hospitalisation rate was observed in the N-3 zone after disasters (appendix pp 47–48).

**Figure 2:**
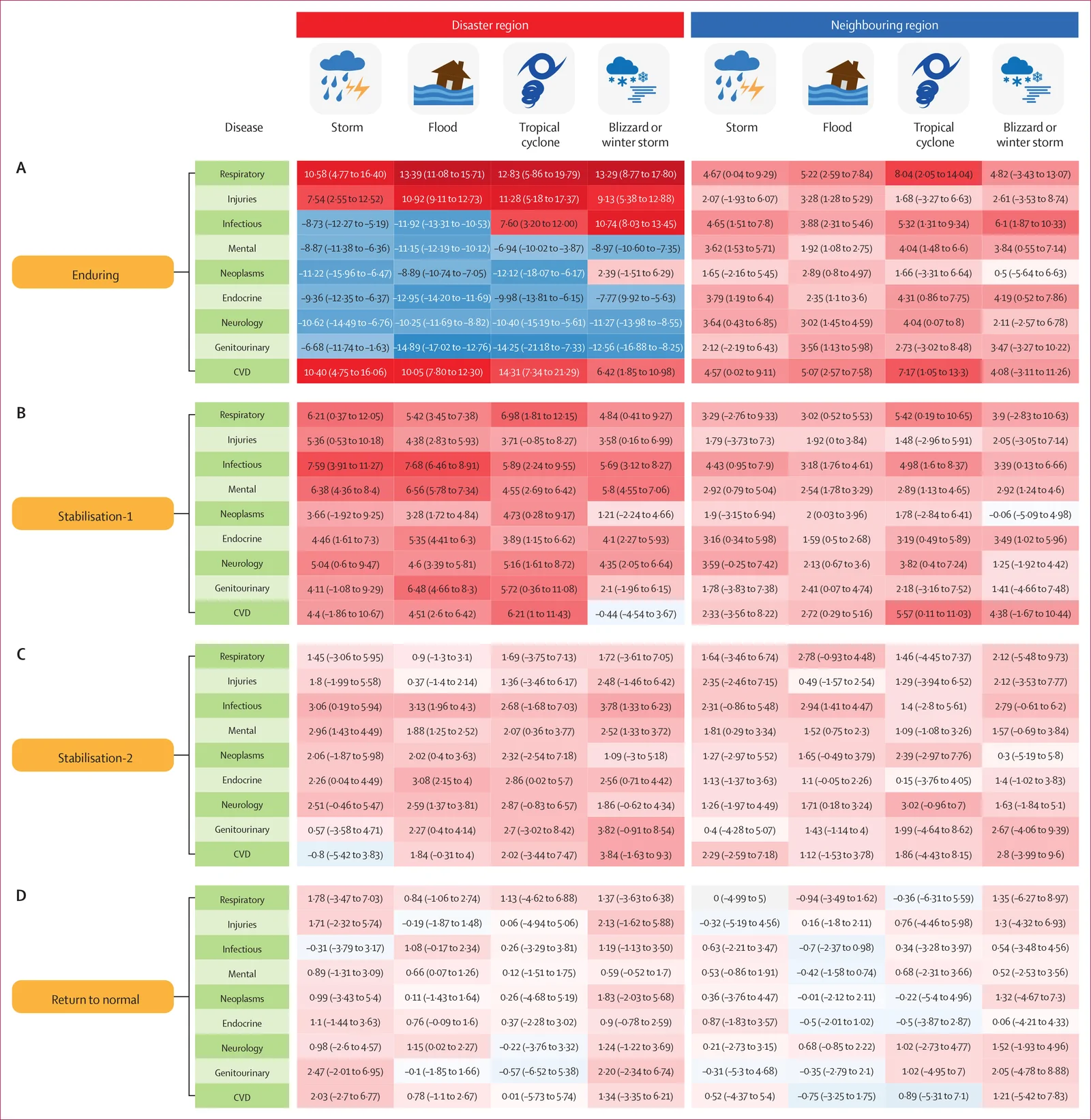
Relative changes of cause-specific hospitalisation rate induced by climate hazards in disaster-exposed and neighbouring counties (0–200 km) in China from 2016 to 2023 All observation windows from hazard-active period (A), enduring to post-hazard periods (B−D): stabilisation-1 (B), stabilisation-2 (C), and return to normal (D) are included. The enduring period covers the event-specific start to end days as documented by the EM-DAT disaster database (Jan 1, 2016 to Dec 31, 2023), whereas each observation window in post-hazard periods lasts for 2 weeks. The values represent the excess relative risks (ie, ERR=RR−1, with RR being the relative risk directly obtained from the propensity score matching–difference-in-differences analytical framework). CVD=cardiovascular disease. Red shading indicates increases in hospitalisation rates relative to matched control counties during that observation window, and blue shading indicates decreases; darker shading represents greater ERR magnitude.

Injury, respiratory diseases, and cardiovascular diseases showed immediate increases in hospitalisation rates relative to matched control counties during the disaster-active period (eg, during flood, the ERR in hospitalisation rates relative to matched control counties were 10·92% [95% CI 9·11 to 12·73] for injuries, 13·39% [11·08 to 15·71] for respiratory diseases, and 10·05% [7·80 to 12·30] for cardiovascular diseases; during tropical cyclones, cardiovascular hospitalisations increased by 14·31% [7·34 to 21·29]); in the disaster-exposed regions, the ERRs for disease-specific hospitalisations declined temporarily (eg, ERR of infectious diseases hospitalisations during the flood-active period: −11·92% [95% CI −13·31 to −10·53]; figure 2A). However, these changes peaked shortly after the hazard ended and the climate gradually returned to normalcy (figures 2B–2D). An estimated increase in hospitalisation rates was also observed in neighbouring regions, although the rates gradually returned to normalcy over time (eg, infectious disease admissions in neighbouring zones increased by 3·18% [1·76 to 4·61] during the 1–2 weeks post floods, and by −0·70% [−2·37 to 0·98] during the 5–6 weeks post floods).

Moreover, cardiovascular, mental, respiratory, and infectious diseases displayed more sensitivity to hazards than other studied disease categories in both the disaster-exposed areas (eg, in case of storms, the ERRs were 10·40% [95% CI 4·75–16·06] for cardiovascular diseases, 6·38% [4·36–8·40] for mental diseases, 10·58% [4·77–16·40] for respiratory diseases, and 7·59% [3·91–11·27] for infectious diseases; in case of floods, the rates were 10·05% [7·80–12·30] for cardiovascular diseases, 6·56% [5·78–7·34] for mental diseases, 13·39% [11·08–15·71] for respiratory diseases, and 7·68% [6·46– 8·91] for infectious diseases) and neighbouring regions (eg, in case of cyclones, the rates were 7·17% [1·05–13·30] for cardiovascular diseases, 4·04% [1·48–6·60] for mental diseases, 8·04% [2·05–14·04] for respiratory diseases, 5·32% [1·31–9·34] for infectious diseases and in case of blizzards, the rates were 3·84% [0·55–7·14] for mental diseases and 6·10% [1·87–10·33] for infectious diseases) (figure 2). In particular, mental disorders had a long-term effect after floods and persisted for 6 weeks.

We observed systematic differences in hazard-related hospitalisation patterns across counties stratified by health-system resilience levels. Specifically, high-resilience disaster-exposed regions showed smaller changes in hospitalisation rates, as indicated by the smaller reductions in the hospitalisation rates during the hazard-active period (eg, for flood-related infectious disease, the rate was −10·44% [95% CI −12·34 to −8·55] in the high-resilience regions *vs* −13·56% [−15·69 to −11·43] in the low-resilience regions; for storm-related respiratory hospitalisations, the rate was 7·21% [0·67–13·75] in the high-resilience regions *vs* 12·13% [5·20–19·05] in the low-resilience regions) and smaller hospitalisation peak pressure during post-hazard periods (eg, for cyclone-related neoplasms, the rates were 4·20% [−1·17 to 9·57] in the high-resilience regions *vs* 5·98% [0·14–10·83] in the low-resilience regions; figure 3A). Generally, compared with low-resilience regions, high-resilience regions showed 19·48–30·03% smaller hazard-related relative changes in hospitalisation rates during the hazard-active period and 27·07–31·08% smaller post-hazard relative changes thereafter (appendix p 31). Similar patterns were observed in neighbouring regions, in which high-resilience regions also showed smaller relative changes in hospitalisation rates (eg, in case of flood-related mental disorders, the rates were 1·70% [0·55–2·86] in high-resilience regions *vs* 2·06% [0·94–3·19] in low-resilience regions during the enduring period; the rates were 2·30% [1·34–3·26] *vs* 2·80% [1·80–3·80] during the stabilisation-1 period; figure 3B).

**Figure 3:**
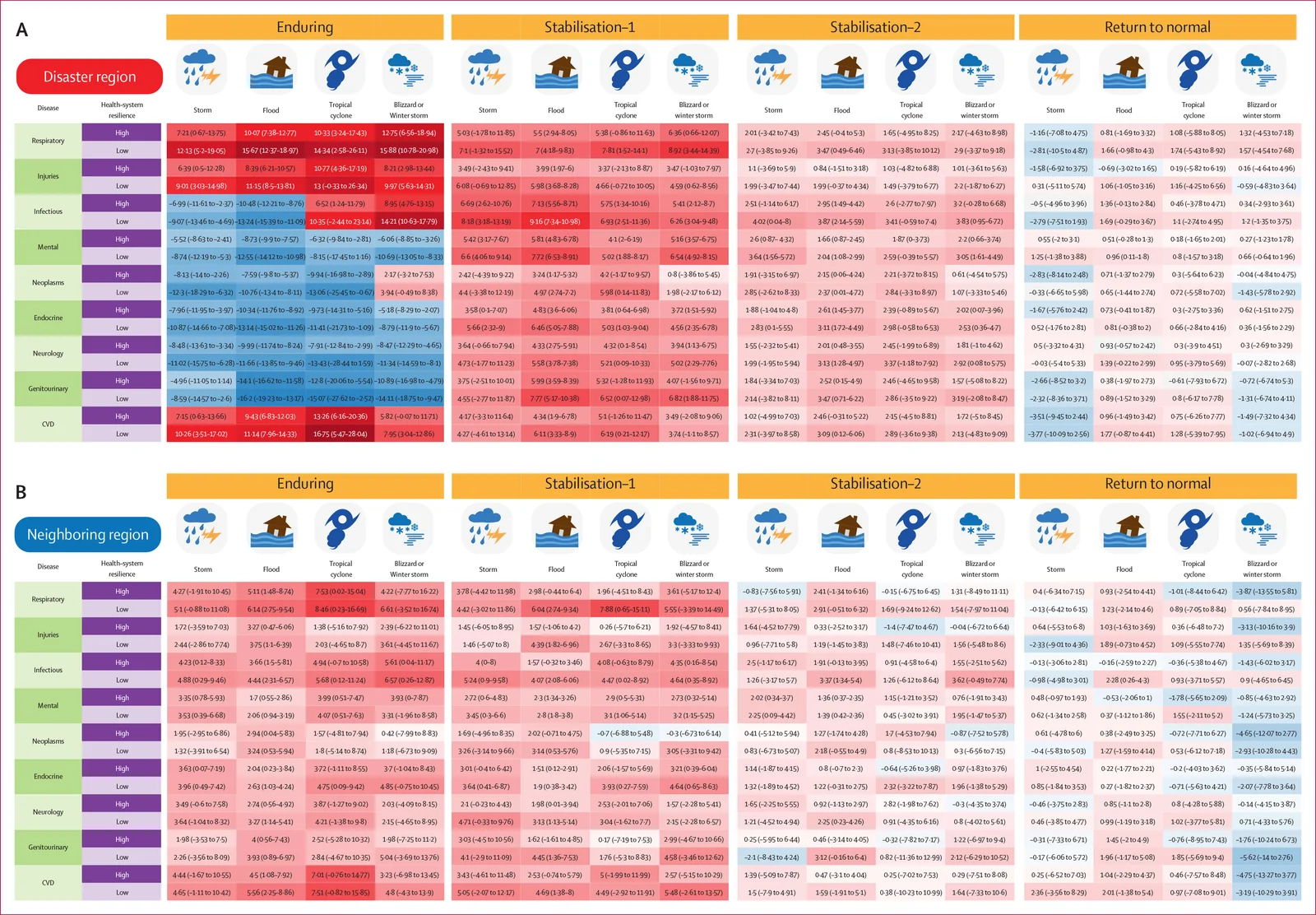
Relative changes of cause-specific hospitalisation rate stressed by climate hazards in disaster-exposed and neighbouring counties (0–200 km) in China from 2016 to 2023, stratified by resilience levels All observation windows from hazard-active period (A), enduring to post-hazard periods (B−D): stabilisation-1 (B), stabilisation-2 (C), and return to normal (D) are included. The enduring period covers the event-specific start to end days as documented by the EM-DAT disaster database (Jan 1, 2016 to Dec 31, 2023), whereas each observation window in lag periods lasts for 2 weeks. The values represent the excess relative risks (ie, ERR=RR−1, with RR being the relative risk directly obtained from the propensity score matching–difference-in-differences analytical framework). CVD=cardiovascular disease. Red shading indicates increases in hospitalisation rates relative to matched control counties during that observation window, and blue shading indicates decreases; darker shading represents greater ERR magnitude.

Climate hazards might intensify patient mobility across disasters and neighbouring regions, showing local and external hospitalisation rate changes in neighbouring zones through different but interconnected admission pathways. Notably, the patient flow from disaster-exposed regions drove the health-care burden in neighbouring zones across all admission pathways (eg, the rates for emergency department admissions after floods were 3·06% [0·24–5·87], those for outpatient admissions after storms were 8·02% [3·36–12·57], and those for interinstitutional transfers after tropical cyclones were 3·64% [1·40–5·88]; appendix p 32). Furthermore, external patient migration was greater in neighbouring high-resilience regions than in low-resilience regions (eg, the rates for patient transfer during floods were 9·63% [8·27–11·00] *vs* 5·39% [3·97–6·81]). In comparison, changes in hospitalisation rates among local residents were smaller in high-resilience neighbouring counties, indicating that health-system resilience might be associated with smaller local hospitalisation changes in neighbouring counties linked to spillover from disaster-exposed regions (eg, the rates for flood-related emergency department admissions were 3·63% [0·39–6·86] for high-resilience regions *vs* 5·99% [3·00–8·98] for low-resilience regions).

Overall, health workforce (relative importance on hazard-associated hospitalisation changes: 14·58% during the hazard-active period and 13·80% during the post-hazard period) and service delivery (14·10% during the hazard-active period and 14·17% during the post-hazard period) emerged as the two key contributors in mitigating disaster-related health impacts (figure 4A). Moreover, the increases in relative importance of financing (increasing by 3·20% for resistance capabilities and 3·29% for recovery capabilities) and longevity (3·46% for resistance and 2·92% for recovery) underscore the need for sufficient government investments in health-care infrastructure, insurance, and primary health care in addressing hazard challenges (appendix p 30).

**Figure 4:**
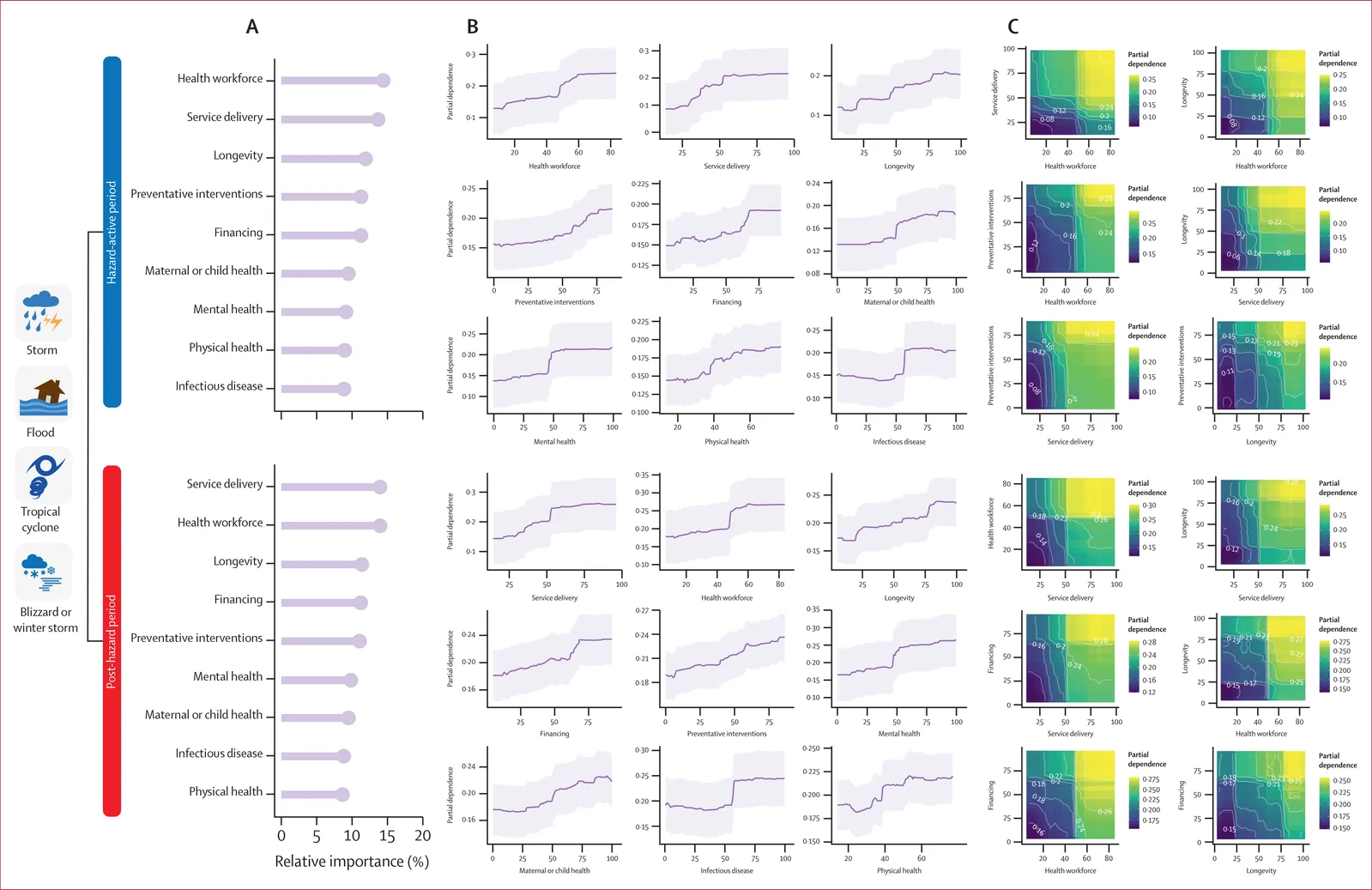
Data-driven policy implications of health-system resilience components for hazard-attributable hospitalisation risk reduction during hazard-active and post-hazard periods (A) Relative importance of resilience metrics on hospitalisation impacts during the hazard-active and post-hazard periods, quantified by the random forest approach. A resilience pillar with higher relative importance indicates that the pillar has higher explanatory power in reducing hospitalisation changes during hazards. (B) Marginal effects (health gains) of resilience components, indicated by the one-dimensional partial dependence plots. The horizontal axis represents the score of resilience metrics, and the vertical axis indicates the health gains (ie, reduction in hazard-related hospitalisation relative changes) when increasing the score of corresponding resilience metrics. To convert to percentage unit, the values should be multiplied by 100%. (C) Illustration of synergistic (cooperative) effects among the top three resilience pillars (identified by the relative importance, quantified by the random forest approach), indicated by the two-dimensional partial dependence plots. The contours indicate the potential public health gains (expressed as percentage reduction in hazard-related hospitalisation relative changes) when increasing the score of corresponding two resilience pillars. To convert to percentage unit, the values should be multiplied by 100%.

Preventive interventions at the county level were strongly correlated with individual-level health status, including mental health (0·79), physical health (0·86), longevity (0·92), and maternal and child health (0·92). This correlation indicates that regions implementing preventive interventions also show improved individual-level resilience components (appendix p 33). In contrast, the weak correlation between service delivery and control of infectious disease (0·16) during the disaster-active period highlight these two elements as potentially independent resilience pillars that could be targeted separately during policy interventions.

The apparent thresholds indicate the needs for context-specific resilience interventions (figure 4B). Low-resilience regions were suggested to prioritise health workforce, service delivery, and financing factors, whereas high-resilience regions might benefit from the continuous investment in preventive interventions and longevity. Regions implementing multiple resilience enhancement strategies might show patterns consistent with synergistic associations, with greater reductions in hospitalisation rates than expected from the sum of individual intervention (figure 4C). For instance, regions in the top quartile for both health workforce and service delivery showed an approximately 18% reduction in disaster-attributable hospitalisation rate increases during the active period, exceeding the combined individual contributions of these factors (6% and 8%). Similar synergistic effects were also observed in service delivery with workforce (16% *vs* 8% and 4%) or financing (14% *vs* 6% and 2%) during the lag periods.

A series of sensitivity analyses were performed to ensure the statistical reliability of our findings. First, both Monte Carlo randomisation approach and principal component analysis-based data-driven approach produced county resilience rankings similar to those from equal weighting approach; thus, the overall health-system resilience rankings were primarily influenced by the underlying indicator and pillar variations rather than by specific weighting choices (appendix p 34). Second, tertile-based resilience classification also showed consistent monotonic reductions, with progressive hazard-related hospitalisation risk reductions from low-resilience to moderate-resilience and high-resilience counties across all hazard types (appendix pp 49–51). This quantitative concordance supports the robustness of the findings for policy implications. Third, the repeated PSM–DID analysis with only county-level random effects indicated that our primary findings were robust to different specifications of the variance structure (appendix pp 52–54). Fourth, the DID estimates without previous PSM were quantitatively similar to our main results, suggesting no significant control county selection bias on estimates (appendix pp 55–56). Fifth, we verified that excluding the pandemic period did not materially alter hazard distributions across regions, resilience strata (appendix p 57), or the estimated DID effects (appendix pp 58–60). Finally, the concordance of marginal effects of resilience metrics given by both partial dependence plots and accumulated local effect approaches showed the reliability of our evidence-based policy recommendations (appendix pp 35–36).

## Discussion

In this study, we used a PSM–DID analytical framework to systematically assess the spatiotemporal health impacts of storms, floods, tropical cyclones, and blizzards or winter storms in China between 2016 and 2023. These hazards substantially affected disaster-exposed regions and indirectly affected neighbouring regions through environmental dispersion and patient migration. Health-care institutions collectively shouldered the crossregional hospitalisation burden during disasters. Hospitalisation rates for cardiovascular diseases, injuries, infectious diseases, respiratory diseases, and mental disorders showed greater relative changes during climate hazards than did those for other conditions. None the less, high-resilience regions were associated with smaller relative changes in hospitalisation rates and with crossregional redistribution of health-care demand across disaster-exposed and neighbouring regions across disaster-exposed and neighbouring regions. By quantifying the relative importance and marginal effects of resilience metrics on disaster-related health outcomes, we developed data-driven guidance for resilience development across diverse contexts.

We developed a comprehensive evaluation framework for health-system resilience that included both static resilience metrics and actual health-care use patterns under hazards. This dual perspective offers essential policy insights. Static resilience assessment is particularly advantageous for regions with minimum historical hazard exposure or insufficient surveillance data for retrospective analysis, providing a practical protocol for self-evaluation of regional health-system resilience,^37^^,^^38^ especially in underdeveloped regions. Analysing the actual health-care use patterns during hazards identified the health-system components most substantially influencing health outcomes, thereby yielding evidence-based, data-driven guidance for disaster risk management.^15^^,^^39^ Static resilience metrics represent structural capacity rather than guaranteed performance. Actual health-care use patterns during disasters provides complementary evidence of health-system resilience. Therefore, in this study, we deliberately integrated both perspectives to enable prospective planning and crossregional comparability using static metrics, and empirical performance analysis validated, which structural components translate into health benefits under real-world disaster conditions.

We comprehensively investigated the associations between nine types of disease-specific hospitalisation changes and four major categories of climate hazards both in disaster-exposed and neighbouring counties. Hospitalisation rates for cardiovascular diseases, injuries, respiratory diseases, infectious diseases, and mental disorders showed greater relative changes during climate disasters, corroborating previous findings from China and other countries.^8^^,^^40^^,^^41^ Because disaster-exposed counties were directly affected by the climate hazards, most cause-specific hospitalisations initially declined during the disaster-active period. Such declines primarily reflect reduced health-care use pattern (eg, infrastructure damage, workforce shortage, and supply chain interruptions) rather than reduced morbidity, thus representing unmet medical needs and service gaps.^7^^,^^15^ In contrast, neighbouring counties were indirectly affected through patient inflows or environmental dispersion, thereby increasing the hospitalisation rates during the disaster phases. Mental disorders and neurological disease also showed relatively long-term effects,^42^ persisting for approximately 6 weeks after floods in disaster-exposed counties and 4 weeks in neighbouring counties. Thus, sustained medical support policies that extend beyond the acute disaster-active phase are required.^43^ Additionally, age-stratified analysis revealed that older adults and children had disproportionately higher hospitalisation burdens after floods and tropical cyclones (appendix p 61), reflecting their heightened physiological vulnerability and restricted adaptation capability.^44^ These disparities underscore the need for age-targeted disaster preparedness interventions, such as pre-positioning medical resources in facilities that serve populations at high risk.^23^ Future work should also explicitly incorporate socioeconomic disadvantages, as people with low income might face compounded vulnerabilities in both exposure and access to care.

The spatiotemporal health-care burden distribution in disaster-exposed and neighbouring regions clarified the complex crossregional use redistribution pattern of health-care systems. During disasters, the surge in health-care demand triggered by climate extremes spontaneously redistributed to adjacent areas through patient migration and environmental dispersion. These dual spillover pathways might have alleviated hospitalisation pressure on health-care facilities in disaster-exposed regions while sufficiently using medical resources in neighbouring zones. Importantly, health-system resilience served as a crucial moderator of these two crossregional spillover pathways. Generally, high-resilience neighbouring counties show higher patient inflows from disaster-exposed counties, evidenced by the remarkable relative increases in visits to emergency admissions, inpatient admissions, and patient transfers across all disaster categories. This higher admission capacity might stem from increased medical resources, robust health-care infrastructure, expanded delivery services, and sufficient workforce in developed regions.^5^^,^^45^ Simultaneously, high-resilience counties also showed lower environmental spillover effects, with comparatively lower hospitalisation stress among local residents (appendix p 32). Such local health-system levels and active patient health-care-seeking behaviours collectively shape the complicated spatiotemporal health impacts in neighbouring regions during climate hazards. Therefore, policy makers should implement pre-disaster coordination protocols^46^ and develop targeted resource allocation frameworks^47^ to optimise the health-care system use efficiency among disaster-exposed and neighbouring counties.

Our findings suggest that targeted policy interventions should be context-appropriate and threshold-aware. Low-resilience regions can focus primarily on health-care financing, workforce capacity, and the accessibility of essential services. In contrast, high-resilience regions, owing to the potential presence of saturation points, can implement multisectoral resilience strategies that might earn synergistic benefits exceeding the sum of individual intervention effects. Partial dependence plot analyses revealed positive associations between multidimensional resilience portfolios and larger predicted health returns (figure 4B, C). Although these observational correlations could not establish causal synergies, this finding echoed the conceptional guidelines.^1^^,^^48^^,^^49^ Moreover, protecting clinically vulnerable populations, including mothers, children, and older adults, and promoting preventive interventions to elevate baseline health levels are of general importance across all regions.^50^ Our analytical framework conceptually aligned with WHO guidelines on disaster risk management and health-system strengthening;^51^ moreover, the framework also advanced the field by offering quantitative insights into the associations between health-system characteristics and hospitalisation outcomes during hazards across diverse regional contexts.

By synthesising the common trends of hospitalisation changes and spillover pathways, we propose an idealised spatiotemporal disaster-related health impact model that illustrates the potential role of health-care systems in crossregional health-care burden redistribution during disasters (figure 5). Specifically, during the active period, disaster-exposed counties showed initial declines in hospitalisation rates because of infrastructure damage, health workforce shortage, and medical service interruption^7^^,^^15^ (figure 5A). This observation aligns with previously reported negative hospitalisation trends in disaster-exposed contexts.^7^^,^^52^ Meanwhile, patient transfers from disaster-exposed regions to the less affected neighbouring counties,^53^ combined with hospitalisation changes, along with environmental dispersion, collectively contributed to the overall elevated hospitalisation pressure in these neighbouring areas (figure 5B, C). Once the hazard ended, hospitalisation rates in disaster-exposed regions showed post-hazard surges that peaked before returning to baseline levels in a gradual manner. Similarly, excess hospitalisation rates in neighbouring counties diminished progressively with time and distance from the disaster epicentre. Notably, regions with high-resilience health systems—whether in disaster-exposed areas or neighbouring area—showed reduced effects across all hazard categories, consistent with WHO’s call for building resilient health systems during climate change adaptation.^51^^,^^54^ In neighbouring zones specifically, high-resilience counties showed a dual pattern: they accommodated higher patient inflow from disaster-exposed regions (thus potentially reducing emergency health-care demand pressure in disaster-exposed regions) while simultaneously experiencing reduced vulnerability to environmental spillover effects.

**Figure 5:**
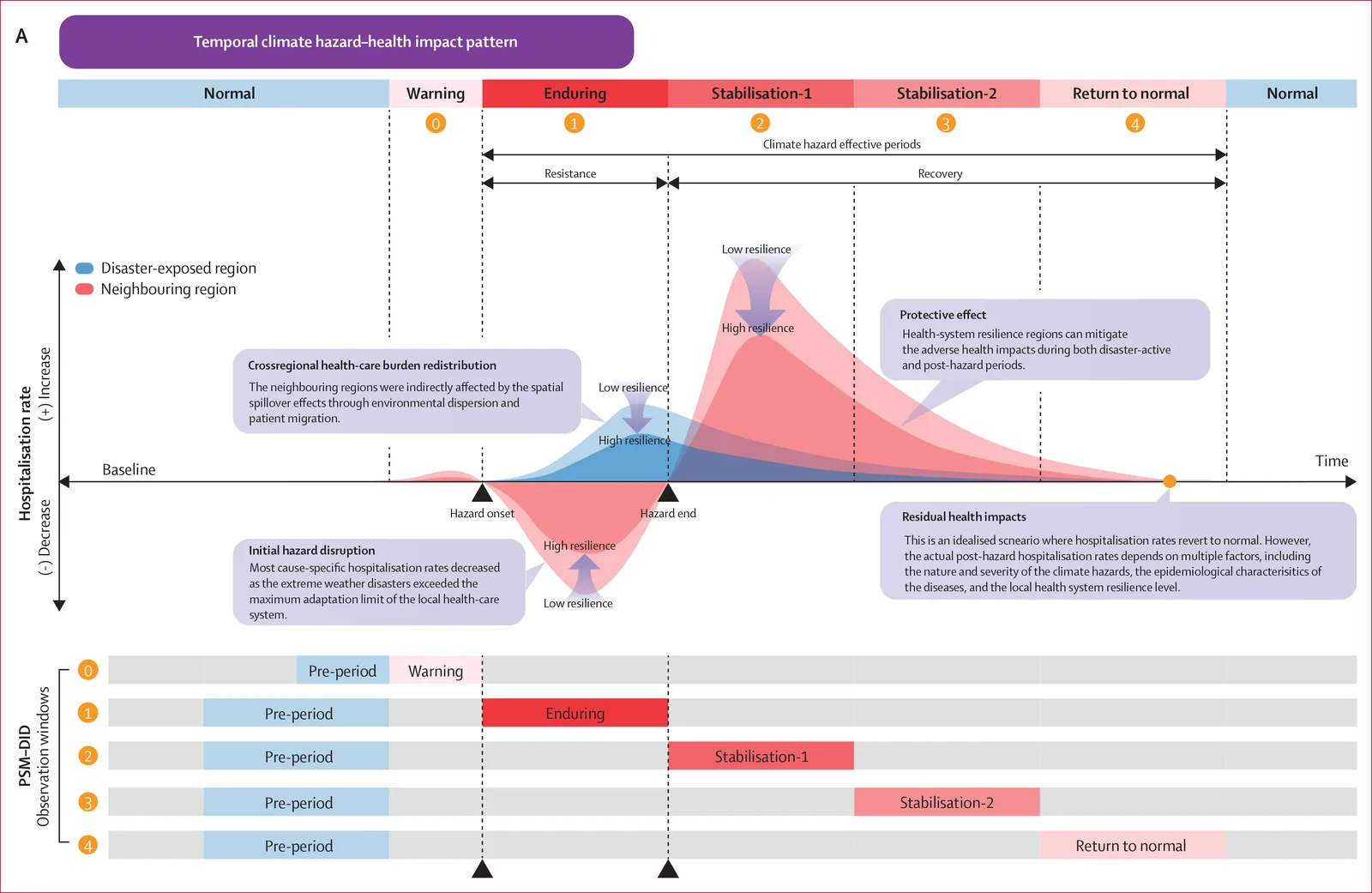

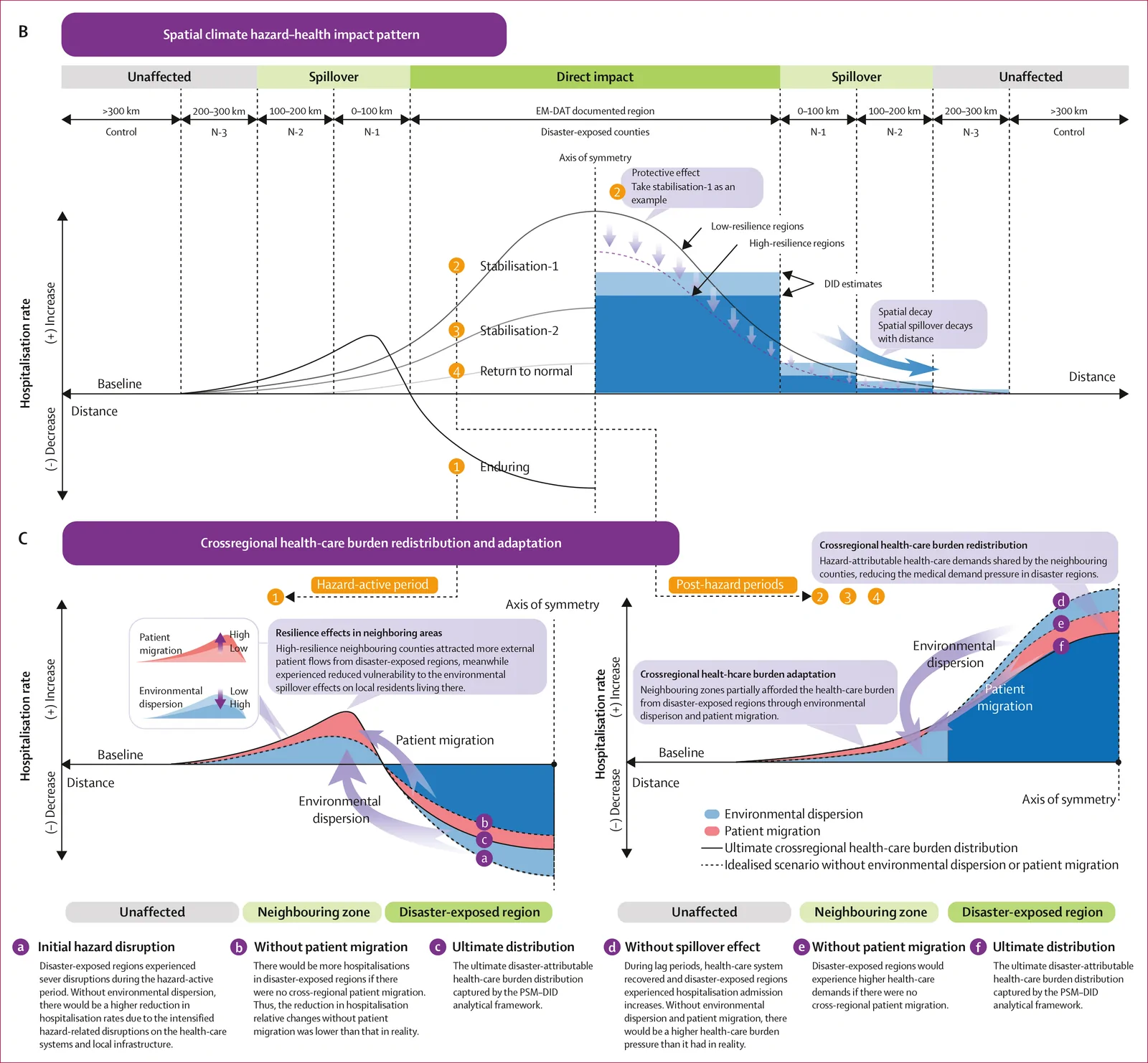
Idealised climate hazard–health impact model This figure illustrates (1) the spatiotemporal disaster–health impact patterns (ie, spatial: direct [red, disaster-exposed regions] and spillover [blue, non-exposed neighbouring regions] effects; temporal: hazard-active and post-hazard periods); (2) the modifying role of local health-system resilience level (ie, high-resilience regions experienced relatively smaller hospitalisation changes during hazards than low-resilience regions); and (3) the spillover pathways (ie, environmental dispersion and patient migration) among disaster-exposed and neighbouring regions. (A) Temporal disaster–health impact patterns in disaster-exposed and neighbouring counties across all disaster phases. (B) Spatial disaster–health impact patterns in disaster-exposed and neighbouring counties. (C) Crossregional health-care burden redistribution, considering the resilience-stratified hospitalisation patterns and spillover pathways of environmental dispersion and patient migration from disaster-exposed to neighbouring regions. In subpanel a, during hazard-active periods, negative hospitalisation changes indicate reduced health-care utilisation. In subfigure c, high-resilience neighbouring counties show relatively high hospitalisation increases from crossregional patient inflows while experiencing low environmental spillover effects. The subpanels (a) to (f) illustrate idealised hospitalisation changes across disaster-exposed and neighbouring regions following exposure to climate hazards under divergent scenarios. During the hazard-active period: (a) represents the idealised initial hospitalisation decrease in disaster-exposed region without spillover effects; (b) represents the health-care utilisation pattern across disaster-exposed and neighbouring regions considering environmental dispersion but no crossregonal patient flows; and (c) represents the ultimate health-care utilisation pattern across disaster-exposed and neighbouring regions considering both environmental dispersion and crossregional patient flows. During the post-hazard periods: (d) represents the idealised initial hospitalisation increase in disaster-exposed region without spillover effects; (e) indicates the health-care utilisation pattern across disaster-exposed and neighbouring regions considering environmental dispersion but no crossregonal patient flows; and (f) represents the ultimate health-care utilisation pattern across disaster-exposed and neighbouring regions considering both environmental dispersion and crossregional patient flows. EM-DAT=emergency events database. PSM–DID=propensity score matching–difference-in-differences.

Our findings might have broader applicability when adapted to diverse health-care system architectures. First, the relationship between resilience and hazard mitigation represents a conceptual principle supported across centralised systems,^55^ decentralised federal systems,^11^ and low-resource settings.^51^ Second, spatial spillover effects requiring crossregional coordination parallel the patterns observed in other contexts,^23^^,^^56^ although coordination mechanisms might differ by governance structure (eg, government-mandated referral protocols in China or, interstate mutual aid compacts in the USA). Third, although threshold-aware intervention optimisation applies universally, health-system metrics should be contextualised to meaningful local indicators (eg, public insurance coverage translate as the total licensed capacity in China but as Medicare or Medicaid coverage in the USA,^57^ or as out-of-pocket expenditure ratios in some developing countries). Therefore, although our quantitative thresholds are context-specific to China, the underlying principles can inform adaptation planning in other health-system architectures after appropriate contextualisation. In this regard, policy makers are suggested to assess structural alignment with China’s model to find out adaptation needs; translate universal principles into locally appropriate mechanisms; and codesign implementation strategies with local stakeholders to ensure feasibility and acceptability.^50^^,^^51^^,^^58^, ^59^, ^60^

This study has some limitations. First, because of rigorous matching, not every affected county was paired with four unaffected controls. Second, our reliance on hospital-based records might have underestimated hazard-related hospitalisation burden and spillover by missing mortality, outpatient or primary care visits, and unreported morbidity (especially mental health conditions and infectious diseases that less require hospitalisation). Third, our measurement of patient migration through non-local hospitalisation admissions captures formal intercounty transfers but likely misses informal patient movement (eg, families directly transporting patients and care-seeking at private facilities). Thus, the quantified spillover burden might underestimate the crossregional hospitalisation pressure, particularly in rural areas. Fourth, although we quantified spillover effects in neighbouring counties through increased local resident hospitalisations, our analysis did not isolate specific mechanisms driving these crossregional impacts. The observed increases could reflect environmental contamination drift, socioeconomic effects, psychological stress, and diagnostic surveillance changes after hazard events.^61^ Thus, although we used the term environmental dispersion for brevity, it also captures aggregated hazard–health consequences in neighbouring counties through multiple pathways. Fifth, although the PSM–DID framework helps to address bias related to disparities in health-care accessibility by incorporating multiple health-care-related and socioeconomic covariates, we relied on hospitalisation records in this study. This reliance might lead to an underestimation of hazard–health impacts, as milder cases and individuals with poor access to health services might not be completely captured. Future research would also benefit from incorporating more comprehensive data sources such as outpatient visits and community health surveys. Moreover, although our findings suggest that health-system resilience is associated with smaller hazard-related changes in hospitalisation rates, the observed mitigation effect might partly reflect unmeasured socioeconomic differences between high-resilience and low-resilience counties. Future research incorporating more comprehensive socioeconomic measures would help to isolate the independent effects of health-system resilience. Sixth, PSM–DID framework cannot fully eliminate potential bias from unobservable time-varying confounders (eg, local economic shocks and concurrent health-care policy changes) that might coincide with disaster events.^62^ Although event–study validations showed no significant prehazard divergent trends, we cannot rule out residual confounding from idiosyncratic time-varying shocks.^63^ Seventh, the exclusion of counties that had concurrent or closely successive hazards (intervals of less than 3 months) ensured analytical validity but might exclude severe compound hazard scenarios (eg, tropical cyclone-induced flooding). As compound events might produce disproportionately larger health impacts than isolated events, our estimates might under-represent the health-care burden attributable to climate hazards. Eighth, we could not perform sophisticated trajectory or intensity stratification analysis (through wind speed,^64^ precipitation,^65^ or property loss^7^) because of EM-DAT data constraints. High-intensity events likely produce vastly different health effects than low-intensity ones, which can confound our estimates, because observed heterogeneity could partly reflect unmeasured differences in hazard severity rather than the true mitigation effect associated with high-resilience counties. Besides, we could not incorporate dynamic, hazard-induced changes in health-system resilience assessment framework because of the absence of hazard intensity data and high-temporal resolution statistics of resilience metrics. Moreover, our DID estimates capture net changes in total hospitalisation volume, which includes both reductions in routine care and increases in disaster-attributable admissions. Because hospital records do not indicate whether a specific admission was causally triggered by the disaster, we cannot empirically separate these processes. Future research incorporating emergency department chief complaints or linked primary care data could enable precise decomposition of routine versus disaster-attributable health-care demand. Finally, the spillover effects might be overstated as the boundary of hazards such as storms, floods, and tropical cyclones are often diffuse and hard to delineate accurately; however, we crossvalidated hazard boundary in EM-DAT at the county level. Despite these limitations, the extended observation windows covering disaster-exposed and multiple neighbouring regions enabled us to robustly characterise the full spectrum of health effects and the health-system-oriented adaptation interventions towards climate hazards.

In summary, this study offers a comprehensive spatiotemporal analysis of nine types of cause-specific hospitalisations during storms, floods, tropical cyclones, and blizzards or winter storms in China from 2016 to 2023. These hazards were associated with substantial changes in hospitalisation rates in disaster-exposed regions and with additional spillover burden in neighbouring zones through environmental dispersion and patient migration. Health-care institutions collectively accommodated the crossregional health-care burden during disasters. Cardiovascular diseases, injuries, respiratory diseases, infectious diseases, and mental disorders were significantly associated with the occurrence of disasters. None the less, health-system resilience is essential in hazard mitigation and crossregional health-care burden redistribution. Finally, enhancing county-scale health workforce and service delivery and promoting individual-level mental, physical, and maternal or child health were identified as potentially important priorities for strengthening local health-system resilience towards climate hazards.

## Data sharing

Owing to patients’ privacy reasons and administrative restrictions, health data cannot be shared publicly but can be accessed on reasonable request to the corresponding author. Other datasets, including but not limited to, the climate hazard records from the EM-DAT international disaster database, the high-resolution (1 km) Köppen–Geiger climate type classification and global distribution data from the GloH2O database, and the statistical attributes used for developing health-system resilience metrics using the National Healthcare System Administration and the National Bureau of Statistics data are publicly available from the corresponding institutions. The codes for both main and supplementary analyses are shared at GitHub and Zenodo.

## Declaration of interests

We declare no competing interests.

## References

[1] Pörtner H-O, Roberts DC, Tignor M (2022). Climate change 2022: impacts, adaptation and vulnerability working group II contribution to the sixth assessment report of the intergovernmental panel on climate change.

[2] Holland JH (2006). Studying complex adaptive systems. J Syst Sci Complex.

[3] Bashan NF, Li W, Wang QR (2024). Dynamics of PM_2.5_ and network activity during extreme pollution events. npj Clim Atmos Sci.

[4] Livneh B, Bjarke NR, Modi PA (2024). Can precipitation intermittency predict flooding?. Sci Total Environ.

[5] Zhao P, Li J, Zhang M (2025). Unequal roles of cities in the intercity healthcare system. Nat Cities.

[6] Ebi KL, Vanos J, Baldwin JW (2021). Extreme weather and climate change: population health and health system implications. Annu Rev Public Health.

[7] Salas RN, Burke LG, Phelan J, Wellenius GA, Orav EJ, Jha AK (2024). Impact of extreme weather events on healthcare utilization and mortality in the United States. Nat Med.

[8] Yang Z, Huang W, McKenzie JE (2023). Mortality risks associated with floods in 761 communities worldwide: time series study. BMJ.

[9] Zeng M, Du J, Zhang W (2019). Spatial-temporal effects of PM_2.5_ on health burden: evidence from China. Int J Environ Res Public Health.

[10] (2024). Spillover effects of targeted malaria interventions benefit neighbouring areas. Nat Med.

[11] Zhong L, Lopez D, Pei S, Gao J (2024). Healthcare system resilience and adaptability to pandemic disruptions in the United States. Nat Med.

[12] Tee Lewis PG, Chiu WA, Nasser E (2023). Characterizing vulnerabilities to climate change across the United States. Environ Int.

[13] Legatum institute (2023). The 2023 Legatum Prosperity Index. https://index.prosperity.com/rankings.

[14] World Economic Forum (Jan 16, 2024). Quantifying the impact of climate change on human health. https://www.weforum.org/publications/quantifying-the-impact-of-climate-change-on-human-health/.

[15] Braithwaite J, Leask E, Smith CL (2024). Analysing health system capacity and preparedness for climate change. Nat Clim Change.

[16] Khatana SAM, Venkataramani AS, Nathan AS (2021). Association between county-level change in economic prosperity and change in cardiovascular mortality among middle-aged US adults. JAMA.

[17] Beck HE, McVicar TR, Vergopolan N (2023). High-resolution (1 km) Köppen-Geiger maps for 1901–2099 based on constrained CMIP6 projections. Sci Data.

[18] WHO (Feb 21, 2024). Health system resilience indicators: an integrated package for measuring and monitoring health system resilience in countries. https://www.who.int/publications/i/item/9789240088986.

[19] Integrated Research on Disaster Risk (March, 2014). Peril classification and hazard glossary (IRDR DATA Publication No. 1). https://council.science/wp-content/uploads/2019/12/Peril-Classification-and-Hazard-Glossary-1.pdf.

[20] Emergency Events Database (2024). Entry criteria: minimal requirements for a disaster to be entered in EM-DAT. https://doc.emdat.be/docs/protocols/entry-criteria/.

[21] Rosvold EL, Buhaug H (2021). GDIS, a global dataset of geocoded disaster locations. Sci Data.

[22] Emergency Events Database (2024). Specific biases: understanding particular data quality concerns in EM-DAT. https://doc.emdat.be/docs/known-issues-and-limitations/specific-biases/.

[23] Brodie M, Weltzien E, Altman D, Blendon RJ, Benson JM (2006). Experiences of Hurricane Katrina evacuees in Houston shelters: implications for future planning. Am J Public Health.

[24] ICD International statistical classification of diseases and related health problems 10^th^ revision. https://icd.who.int/browse10/2019/en.

[25] Bondarenko M, Priyatikanto R, Tejedor-Garavito N (2025). Estimates of 2015–2030 total number of people per grid square broken down by gender and age groupings at a resolution of 30 arc (approximately 100m at the equator). R2025A version v1. Global Demographic Data Project - Funded by The Bill and Melinda Gates Foundation (INV-045237). WorldPop - School of Geography and Environmental Science, University of Southampton. https://hub.worldpop.org/doi/10.5258/SOTON/WP00841.

[26] General Office of the State Council (2015). Notice of the General Office of the State Council on issuing the outline for the planning of the national medical and health service system (2015-2020): General Office of the State Council. https://www.gov.cn/zhengce/content/2015-03/30/content_9560.htm.

[27] Regional Studies Association (2025). The Hu Huanyong Line. https://www.regionalstudies.org/rsa-blog/hu-huanyong-line/.

[28] Wang T, Shi H, Liang Z (2025). Causal link between humid heatwaves and ischemic heart disease: assessing hospitalisations and economic burden across 955 Chinese counties. BMC Med.

[29] Anselin L (1995). Local indicators of spatial association—LISA. Geogr Anal.

[30] Sun L, Abraham S (2021). Estimating dynamic treatment effects in event studies with heterogeneous treatment effects. J Econom.

[31] Baker AC, Larcker DF, Wang CCY (2022). How much should we trust staggered difference-in-differences estimates?. J Financ Econ.

[32] Grömping U (2009). Variable importance assessment in regression: linear regression versus random forest. Am Stat.

[33] Hastie T, Tibshirani R, Friedman J (2009).

[34] Apley DW, Zhu J (2020). Visualizing the effects of predictor variables in black box supervised learning models. J R Stat Soc B: Stat Methodol.

[35] Daw JR, Hatfield LA (2018). Matching and regression to the mean in difference-in-differences analysis. Health Serv Res.

[36] Daw JR, Hatfield LA (2018). Matching in difference-in-differences: between a rock and a hard place. Health Serv Res.

[37] Choptiany JMH, Philips S, Graube BE (2017). SHARP: integrating a traditional survey with participatory self-evaluation and learning for climate change resilience assessment. Clim Dev.

[38] Ebi KL, Kovats RS, Menne B (2006). An approach for assessing human health vulnerability and public health interventions to adapt to climate change. Environ Health Perspect.

[39] Field CB, Barros V, Stocker TF (2012).

[40] World Economic Forum (June, 2024). Health impacts of climate change: evidence landscape and role of private sector. https://www3.weforum.org/docs/WEF_Health_Impacts_of_Climate_Change_2024.pdf.

[41] He C, Kim H, Hashizume M (2024). The overlooked health impacts of extreme rainfall exposure in 30 East Asian cities. Nat Sustain.

[42] Alderman K, Turner LR, Tong S (2012). Floods and human health: a systematic review. Environ Int.

[43] Cheema HA, Rehan ST, Shahid A, Head MG, Jawad MY, Shah J (2023). The mental health of children in flood-affected areas in Pakistan needs urgent attention. Lancet Psychiatry.

[44] Ryan B, Franklin RC, Burkle FM (2015). Identifying and describing the impact of cyclone, storm and flood related disasters on treatment management, care and exacerbations of non-communicable diseases and the implications for public health. PLoS Curr.

[45] Haldane V, De Foo C, Abdalla SM (2021). Health systems resilience in managing the COVID-19 pandemic: lessons from 28 countries. Nat Med.

[46] Rodríguez-Espíndola O, Albores P, Brewster C (2018). Disaster preparedness in humanitarian logistics: a collaborative approach for resource management in floods. Eur J Oper Res.

[47] Hick JL, Hanfling D, Cantrill SV (2012). Allocating scarce resources in disasters: emergency department principles. Ann Emerg Med.

[48] Adger WN (2003). Social capital, collective action, and adaptation to climate change. Econ Geogr.

[49] Cheng JJ, Berry P (2013). Health co-benefits and risks of public health adaptation strategies to climate change: a review of current literature. Int J Public Health.

[50] WHO (Nov 21, 2023). Protecting maternal, newborn and child health from the impacts of climate change: a call for action. https://www.who.int/publications/i/item/9789240085350.

[51] WHO (June 19, 2024). Building health system resilience to public health challenges: guidance for implementation in countries. https://www.who.int/publications/i/item/9789240094321.

[52] Parks RM, Anderson GB, Nethery RC, Navas-Acien A, Dominici F, Kioumourtzoglou MA (2021). Tropical cyclone exposure is associated with increased hospitalization rates in older adults. Nat Commun.

[53] Sheldon TL, Zhan C (2022). The impact of hurricanes and floods on domestic migration. J Environ Econ Manag.

[54] WHO (Oct 12, 2023). Climate change. https://www.who.int/news-room/fact-sheets/detail/climate-change-and-health.

[55] UK Health Alliance on Climate Change (June, 2025). Building a climate-resilient health system in the UK. https://s41874.pcdn.co/wp-content/uploads/Climate-Resilient-Health-System-Report-1.pdf.

[56] Berggren RE, Curiel TJ (2006). After the storm–health care infrastructure in post-Katrina New Orleans. N Engl J Med.

[57] US Department of Health and Human Services (2022). What’s the difference between Medicare and Medicaid?. https://www.hhs.gov/answers/medicare-and-medicaid/what-is-the-difference-between-medicare-medicaid/index.html.

[58] WHO (May 6, 2021). WHO guideline on health workforce development, attraction, recruitment and retention in rural and remote areas. https://www.who.int/publications/i/item/9789240024229.

[59] WHO (June 10, 2015). Operational framework for building climate resilient health systems. https://www.who.int/publications/i/item/operational-framework-for-building-climate-resilient-health-systems.

[60] WHO (July 30, 2019). Health emergency and disaster risk management framework. https://www.who.int/publications/i/item/health-emergency-and-disaster-risk-management-framework.

[61] Galea S, Nandi A, Vlahov D (2005). The epidemiology of post-traumatic stress disorder after disasters. Epidemiol Rev.

[62] Goodman-Bacon A (2021). Difference-in-differences with variation in treatment timing. J Econom.

[63] Freyaldenhoven S, Hansen C, Shapiro JM (2019). Pre-event trends in the panel event-study design. Am Econ Rev.

[64] Young R, Hsiang S (2024). Mortality caused by tropical cyclones in the United States. Nature.

[65] He C, Breitner-Busch S, Huber V (2024). Rainfall events and daily mortality across 645 global locations: two stage time series analysis. BMJ.

