# Supplementary material for: Direct and spillover hospitalisation patterns during climate hazards across regions of different health-system resilience levels in China: a nationwide retrospective analysis
Source: Lancet Planet Health. 2026 Sep 1;10(8):None. doi: 10.1016/j.lanplh.2026.101512 (PMC13627082; doi:10.1016/j.lanplh.2026.101512)
Supplement: Supplementary file 1 [file mmc1.pdf]

THE LANCET
Planetary Health

Supplementary appendix

This appendix formed part of the original submission and has been peer reviewed.
We post it as supplied by the authors.

Supplement to: Wang T, Shi H, Shi Y, et al. Direct and spillover hospitalisation patterns during climate hazards across regions of different health-system resilience levels in China: a nationwide retrospective analysis. *Lancet Planet Health* 2026. https://doi.org/10.1016/j.lanplh.2026.101512

Supplementary Information

Direct and spillover hospitalization impacts of climate hazards across health system resilience levels in China: a nationwide retrospective analysis

Teng Wang[1,2,3,*], Hanxu Shi[1,*], Yujie Shi[1], Peng Wang[3], Weiwen Li[3], Jin Lv[4], Wenliang Yao[5], Zhisheng Liang[1], Mintao Su[1], Siwen Li[1], Bin Wang[6], Murray Ramanathan[7], Shaym Biswal[8], Steve Hung-Lam Yim[9,10,11], Ming Xu[1], Wenjia Cai[12], Wei Huang[13], Tiantian Li[14], Cunrui Huang[15], Haidong Kan[16], Minghui Ren[1,17], Gordon G. Liu[18,#], Chung Yee Kwok[2,#], Zhenyu Zhang[1,18,19,#]

- Table of Contents -

■ **Texts**

Text 1 | The health system resilience metrics 4
Text 2 | Climate hazard inclusion criteria, cross-validation, and observation window setting justification 7
Text 3 | CMC Hospital Network and daily cause-specific hospitalization records 8
Text 4 | County classification 9
Text 5 | Spillover pathways 10
Text 6 | Statistical analysis 10
Text 7 | Hospitalization risk disparities between resilience strata 13
Text 8 | Data-driven adaptation policy implications 15

■ **Figures**

Fig. 1 | CMC Hospital Network and population density distribution in China 18
Fig. 2 | CMC Hospital Network covered and uncovered counties in subdivided regions in China 19
Fig. 3 | High resolution (1 km) local climate type in China using Köppen-Geiger classification. 20
Fig. 4 | Standardized mean differences of covariates before and after propensity score matching (PSM). 21
Fig. 5 | Histograms of propensity score before and after PSM. 22
Fig. 6 | Parallel trend tests for climate-sensitive diseases under floods. 23
Fig. 7 | Parallel trend tests for climate-sensitive diseases under storms. 24
Fig. 8 | Parallel trend tests for climate-sensitive diseases under tropical cyclones. 25
Fig. 9 | Parallel trend tests for climate-sensitive diseases under blizzard/winter storms. 26
Fig. 10 | Climate hazards in China from 2016 to 2023. 27
Fig. 11 | Comparison of health system resilience metrics between low and high-resilience regions, indicated by the standardized mean differences (SMD). 28

Fig. 12 | Relative importance of health system resilience metrics on overall resilience score and hospitalization risk disparities during and after climate hazards. 30
Fig. 13 | Hospitalization risk disparities between high- and low-resilience regions during hazard-active and post-hazard periods. 31
Fig. 14 | Stratification analysis on the spillover pathways in 0 – 200 km neighboring counties. 32
Fig. 15 | Heatmap of correlation strength between health system resilience metrics during the hazard-active and post-hazard periods. 33
Fig. 16 | Sensitivity analysis of health system resilience assessment using Monte Carlo randomization method. 34
Fig. 17 | Sensitivity analysis using accumulated local effect (ALE) plots of health system resilience metrics on hazard-active period metric toward Climate hazards. 35
Fig. 18 | Sensitivity analysis using accumulated local effect (ALE) plots of health system resilience metrics on post-hazard period metric toward Climate hazards. 36
Fig. 19 | Hazard cross-validation between the EM-DAT and the GDIS databases. 37
Fig. 20 | CMC Hospital Network. 38

■ **Tables**

Table 1 | Health system resilience metrics: pillars and indicators. 39
Table 2 | Patient characteristics registered by the Chinese Multiple Counties Hospital Network in 2016-2023. 41
Table 3 | County-scale climate hazard exposure characteristics 42
Table 4 | Relative changes of cause-specific hospitalization rates in N-I (0-100 km) regions. 43
Table 5 | Relative changes of cause-specific hospitalization rates in N-II (100-200 km) regions. 45
Table 6 | Relative changes of cause-specific hospitalization rates in N-III (200-300 km) regions. 47
Table 7 | Sensitivity analysis of tertile-based resilience classifications 49
Table 8 | Sensitivity analysis using county-level random effects. 52
Table 9 | Sensitivity analysis of DID estimates without PSM 55
Table 10 | Representativeness of county-hazard exposures in the pandemic-excluded study period 57
Table 11 | Sensitivity analysis excluding the pandemic period. 58
Table 12 | Age-stratification analysis following climate hazards in China during 2016-2023 61
Table 13 | Relative changes of cause-specific hospitalization rates in disaster-exposed regions at warning period. 62
Table 14 | Relative changes of cause-specific hospitalization rates in neighboring regions (0-200 km) at warning period. 63

Table 15 | Relative changes of cause-specific hospitalization rates in neighboring regions (0-100 km) in western China. 64
Table 16 | Relative changes of cause-specific hospitalization rates in neighboring regions (100-200 km) in western China. 66
Table 17 | Relative changes of cause-specific hospitalization rates in neighboring regions (200-300 km) in western China. 68
Table 18 | Relative changes of cause-specific hospitalization rates in neighboring regions (0-100 km) in estern China. 70
Table 19 | Relative changes of cause-specific hospitalization rates in neighboring regions (100-200 km) in eastern China. 72
Table 20 | Relative changes of cause-specific hospitalization rates in neighboring regions (200-300 km) in eastern China. 74
Table 21 | Summary of matched and unmatched counties using propensity score matching (PSM) for climate-related hazard analyses. 76

■ **Reference** 77

Text 1 | The health system resilience metrics

1.1 Conceptual framework and indicator selection

Referring to the WHO health system resilience indicator guidelines[1] and extensive literature review, we developed a comprehensive evaluation framework for health system resilience. In total, this framework encompasses 39 indicators grouped into nine pillars: i) service delivery, ii) health workforce, iii) preventative interventions, iv) longevity, v) financing, vi) physical health, vii) mental health, viii) maternal/child health and ix) infectious disease (**Supplementary Table 1**). These indicators were aggregated during the entire study period (2016-2023).

Four principal criteria guided the indicator selection process. First, theoretical alignment with WHO's conceptual framework of health system resilience[2-4], which delineates three core capacities: adaptive capacity (the ability to adjust strategies and operations in response to changing conditions), absorptive capacity (the ability to maintain essential functions during acute shocks), and transformative capacity (the ability to fundamentally reorganize structures and processes when existing approaches prove inadequate). Second, comprehensive resilience review (**Supplementary Table 1**) and empirical grounding in literature demonstrating associations between health system characteristics and health outcomes during climate-related hazards[5-12]. Third, data availability[13,14] and quality considerations, specifically ensuring complete spatial coverage across all Chinese counties and temporal consistency throughout the study period (2016-2023). Fourth, relevance to climate hazard vulnerability, assessed through expert consultation and theoretical consideration of pathways linking health system resilience to hazard-related health mitigation and protection.

Notably, to ensure meaningful comparison across counties with varying sizes and populations, all indicators were converted to per capita or area-averaged values[15]. We used fundamental indicators rather than composite indices to avoid redundancy and double counting of base indicators.

1.2 Principal component analysis (PCA) and negative indicator identification

A methodological challenge in aggregating diverse health system indicators is that certain metrics may exhibit inverse relationships with overall resilience. For example, higher medicine price indices may reflect reduced affordability and access, emergency department visit rates may proxy for preventable morbidity burden, and foodborne disease incidence may indicate public health surveillance gaps. Direct summation of such indicators without adjustment would introduce bias into composite resilience scores.

To address this objectively, we employed principal component analysis (PCA) to identify indicators with negative contributions to overall health system resilience. This approach leverages the covariance structure of the 39 indicators rather than relying on subjective judgment. Based on comprehensive literature review (**Supplementary Table 1**), most indicators were expected to positively correlate with resilience. We therefore used the sign of the first principal component (PC1) loading as an empirical discriminator: indicators with negative PC1 loadings (i.e., those that co-vary inversely with the dominant positive pattern) were identified as inversely related to resilience

(**Supplementary Table 1**). Prior to normalization and aggregation, indicators with negative PCA1 loadings were transformed by multiplying their values by -1 to align their contributions with the broader positive resilience assessment framework.

1.3 Normalization and aggregation

Following adjustment for indicator directionality, we applied the distance-to-frontier normalization approach[16] to rescale all indicators onto a common [0, 1] scale. This approach sets benchmarks based on observed data distributions rather than theoretical maxima, enhancing comparability across diverse contexts. Mathematically, for each indicator I, the normalized value is:

$$\tilde{I} = \frac{I - I_{min}}{I_{max} - I_{min}} \tag{S1}$$

where I_{max} and I_{min} correspond to the 90th and 10th percentiles, respectively, to mitigate the influence of extreme outliers while preserving substantive inter-county variation.

After normalization, we aggregated indicators within each pillar using a widely accepted equal-weighting approach[17,18]. For each pillar, we assigned a total mark of 100 therefore the pillar score was calculated as:

$$\mathcal{P} = 100 \cdot \frac{1}{n}\sum_{i=1}^{n} \tilde{I}_i \tag{S2}$$

where n is the number of indicators in each pillar. Subsequently, the overall health system resilience score for each county was calculated by equally weighting the nine pillars:

$$\mathcal{R} = \frac{1}{m}\sum_{i=1}^{m} \mathcal{P}_i \tag{S3}$$

with $m = 9$ representing the total number of pillars. Counties were then ranked according to their overall resilience scores, with those in the top half classified as "high-resilience" and those in the bottom half as "low-resilience" counties.

1.4 Rationale for equal weighting

Equal weighting across indicators and pillars was adopted for several methodological reasons. First, it minimizes subjective bias when theoretical or empirical grounds for differential weighting are uncertain[19,20]. Health system resilience is inherently multidimensional[1], and prioritizing specific pillars requires context-dependent value judgments that may not generalize across diverse county settings[21]. Second, equal weighting aligns with international best practices for composite index construction, such as the Human Development Index[21] (HDI) and OECD guidelines[22]. Third, our sensitivity analyses using Monte Carlo randomization approach and PCA-based data-

driven approach (see **Text 1.5** below) demonstrate that resilience rankings were highly robust to alternative weighting schemes, suggesting that inter-county variation in underlying indicator values rather than specific weights drives the primary variance in composite resilience scores.

1.5 Sensitivity analysis:

(1) Monte Carlo randomization of weights

It is noteworthy that, in the aggregation process, each indicator and pillar was assigned equal weight to promote objectivity and minimize subjective influence[18], consistent with prevailing practices in the construction of composite indices[15,17,18]. Nevertheless, we recognized that the common equal-weight assignment represents only one of many plausible approaches from different grounds. To investigate the impact of different weighting schemes and to ensure the robustness and justification of our resilience assessment framework, we examined the sensitivity of resilience rankings to alternative weighting matrix[18] generated by the Monte Carlo randomization simulations (**Supplementary Fig. 16**). Results demonstrated a strong correlation between resilience rankings derived from the equal-weight approach and those from randomized weights, indicating that resilience rankings were primarily influenced by underlying indicator and pillar variations rather than by specific weighting choices. This sensitivity analysis supported the robustness and validity of our comprehensive resilience assessment framework even under diverse weighting scenarios.

(2) PCA-based data-driven weighting

In addition, we examined whether data-driven weighting based on PCA loadings would alter county resilience rankings or substantive conclusions. We conducted PCA on all 39 indicators and examined the distribution of PC1 (first principal component) loadings. Given the multidimensional nature of health system resilience, loadings were relatively dispersed (range: -0.208 to 0.305, **Supplementary Table 1**) with no single indicator dominating PC1, consistent with composite index theory[19,23]. We retained indicators with absolute loadings greater than the median absolute loading ($|loading| > 0.135$), yielding 19 core indicators that represent above-median contributors to the primary variance component. We then calculated county resilience scores using PC1 loading values as weights, normalizing weights to sum to unity within each pillar. Results demonstrate clear ranking concordance between equal weighting and PCA-weighted resilience ranking (**Supplementary Fig. 16**), indicating that data-driven weighting produced substantively similar county resilience classification.This set of sensitivity analysis indicates that our findings are robust to the choice between equal weighting and data-driven weighting schemes.

(3) Alternative resilience classification schemes

In our primary analysis, counties were dichotomized into high-resilience (top 50%) and low-resilience (bottom 50%) groups based on the median resilience score. While this binary classification ensures adequate statistical power and provides clear policy thresholds, it may mask resilience effects gradients. To address this limitation, we conducted complementary sensitivity analyses using tertile county classification: low-

resilience (bottom third), moderate-resilience (middle third), and high-resilience (top third). Results showed consistent monotonic relationships, with progressive decreases in hazard-hospitalization risks from low-resilience to moderate- and high-resilience counties across all hazard types (**Supplementary Table 7**). Such quantitative concordance across our primary and supplementary sensitivity analysis supports the validity and generalizability of our findings for policy applications.

Text 2 | Climate hazard inclusion criteria, cross-validation, and observation window setting justification

The EM-DAT compiled hazard information from a wide range of sources, including United Nations agencies, non-government organizations, reinsurance companies, research institutes, and press agencies[24]. For a natural hazard to be included in the EM-DAT, at least one of the following entry criteria must be met[25]: 1) A minimum of ten fatalities (including dead and missing persons); 2) At least a hundred individuals affected (including injured or homeless); or 3) A call for international assistance request or an emergency declaration. Additionally, the EM-DAT documents historically significant hazards described as “the worst disaster in a county or region” or as events causing considerable damage.

Notably, currently there exists no universally accepted inclusion criteria or completely accurate disaster databases[26]. Despite this, EM-DAT remains the only comprehensive, openly accessible disaster database with global and long-term coverage, cataloging nearly all types of natural hazards since the 1960s based on the 2014 IRDR Peril Classification and Hazard Glossary[27]. This comprehensive scope, together with our focus on extreme climate hazards at the county level, justified the selection of EM-DAT for this study. Although other disaster databases, such as the Geocoded Disaster[28] (GDIS), the Dartmouth Flood Observatory[29] (DFO), and the Global Flood Database[30] (GFD), may offer higher spatial resolution, they were limited by shorter temporal coverage (with DFO ending in 2021 and both GDIS and GFD ending in 2018 for China) or by single-hazard coverage (DFO and GFD focused exclusively on floods, and GDIS did not record blizzard/winter storm events).

To ensure the validity and completeness of hazard records, we cross-validated EM-DAT against the GDIS database to check for inconsistencies in timing, geographic scope, or hazard quality (**Supplementary Fig. 19**). Besides, the Centre Research on the Epidemiology of Disasters (CRED) claimed that they undertakes annual review and cross-validation to maintain the reliability of EM-DAT records[31]. All these measures underpin the robustness and reliability of our hazard-health impact analysis.

To capture the full picture of disaster impacts, we set a series of sequential observation windows spanning the whole hazard-effective periods: warning, enduring, and post-hazard slots. For each event, assume the hazard lasted for T_e days with its onset as day 1, the observation windows for the pre- and post-period comparison in the DID framework were defined as follows (**Fig. 5a**):

(1) Warning: pre- vs. post-period as day [-13, -7] vs. [-6, 0];

(2) Enduring: [-6-T_E, -7] vs. [1, T_E];

(3) Stabilization-I: [-20, -7] vs. [T_E+1, T_E+14];

(4) Stabilization-II: [-20, -7] vs. [T_E+15, T_E+28]; and

(5) Return to normal: [-20, -7] vs. [T_E+29, T_E+42].

Notably, a warning period was set one week prior to the disaster to examine the potential disruption preceding disaster initiation, and to avoid analyzing the shifted hospitalization rates, if any, in the DID pre-period (**Supplementary Table 13 & 14**). The enduring period represented the immediate health impact of disasters, while the subsequent two-week windows of stabilization-I, stabilization-II, and return to normal captured the lag effects. Our observation windows stopped at six weeks after the disaster ended, as our study focused on the short-term impacts of climate hazards, and there was no statistically significant residual impact typically observed for most diseases. The total length of our observation windows for lag effect is comparable with existing literature[32]. Moreover, we excluded the counties that experienced more than one disaster simultaneously. Also, counties with disaster intervals less than three months were omitted due to the disruption of the lag effect of previous hazard on the pre-period of subsequent hazard. Our pre-post window configurations and county inclusion/exclusion criteria were similar to previous studies[32].

Moreover, we applied uniform 2-week observation windows across all hazard types to ensure analytical consistency and cross-hazard comparability. Our DID analyses demonstrated consistent temporal patterns across all hazard types (**Fig. 2**), with peak hospitalization pressure occurring within weeks 1-2 and progressive decline to baseline by weeks 5-6 after hazard cessation. This consistency supports the validity of our standardized temporal settings.

Text 3 | CMC Hospital Network and daily cause-specific hospitalization records

The daily cause-specific hospitalization data were obtained from the National Inpatient Medical Record Dataset documented by the CMC Hospital Network, covering most of the administrative regions in China. Since 2016, more than 3,000 hospitals have been involved. In this study, we selected 1,607 hospitals whose annual hospitalization admissions more than 1,000 (**Supplementary Figs. 1 & 20**). Patients' information, including but not limited to their demographic (sex, age), clinical diagnoses and payments (diagnostic-related group (DRG), payment sources such as out-of-pocket, insurance, government poverty support), hospitalization sources (from emergency departments (EDs), outpatient or transferred from other healthcare institutions) and home address were documented.

Population coverage and representativeness: The CMC Hospital Network captures approximately 10% of China's total population, estimated from: (i) the proportion of CMC hospitalizations relative to national hospitalization admissions reported by the National Healthcare Service Administration, and (ii) the ratio of CMC hospitals to all hospitals in China during 2016-2023. Despite this seemingly modest proportion, the sample is nationally representative for three reasons: First, CMC hospitals serve catchment areas covering most of China's densely populated counties, spanning diverse climate zones, geographic regions, and socioeconomic contexts that mirror national population distribution (**Supplementary Fig. 1**). Second, the network

includes diverse hospital types (tertiary, secondary, private, and primary care facilities), capturing a broad spectrum of healthcare utilization patterns. Third, the age-sex distribution and disease patterns in CMC data align closely with national statistics from the China Health Statistics Yearbook[33].

Construction of population denominators: To calculate hospitalization rates, we estimated the geographic catchment area of each CMC hospital using ArcGIS Pro. Specifically, we calibrated the service radius (r = 16 km) such that the total population covered by circular catchment areas (with overlapping areas counted once) approximates 10% of China's population. We then applied this service radius to high-resolution population density data[34] to estimate the population at risk within each hospital's catchment area. For counties with multiple CMC hospitals, catchment populations were aggregated while avoiding double-counting overlapping zones. This approach ensures that hospitalization rates reflect actual healthcare service areas rather than arbitrary administrative boundaries.

ICD-10 codes: In accordance with the 10th revision codes[35] of international classification, CVD (I00-I99), respiratory disease (J00-J99), hazard-related injuries (S00-S99; T00-T98; V00-V99; X00-X99; W00-W99), mental disorders (F00-F99), infectious disease (A00-A99; B00-B99), neurological disease (G00-G99), neoplasms (C00-C97; D00-D48), endocrine disease (E00-E35, E50-E90), genitourinary disease (N00-N99; O00-O99), and other disease (D50-D89; E40-46; H00-H95; K00-K93; L00-L99; M00-M99; R00-R99; Y00-Y98; P00-P96; Q00-Q99; U00-U85; Z00-Z99) were defined (**Supplementary Table 2**).

Text 4 | County classification

We systematically classified the counties into three distinct groups, based on their geographic proximity to disaster-exposed areas: disaster-exposed counties, neighboring counties, and control counties. Specifically, disaster-exposed counties were those directly impacted by the climate hazards. Neighboring counties were the neighbors surrounding the disaster-exposed counties, potentially affected by the indirect spillover impact. To further capture spatial heterogeneity, neighboring counties were sub-categorized as N-I, N-II, and N-III counties with spanning distances of 0-100 km, 100-200 km, and 200-300 km, respectively. These spatial thresholds were determined by:

(1) the mean geographical distance of hospitals in the CMC Hospital Network (62.57 km, **Supplementary Fig. 20**);

(2) the Chinese Government's National Medical and Healthcare Service System Plan[36], where county-level comprehensive hospitals should offer healthcare services within a 50 km radius (i.e., 100 km hospital-hospital distance); and

(3) the geographic threshold of health impacts (see DID estimates in overall, dense east, and sparse west China geographic regions in **Supplementary Table 4-6, 15-20**). Results showed that hazard-hospitalization impacts in both dense east and sparse west regions become no significant within the 200-300 km N-III counties (Note: except tropical cyclones, which concentrated in costal eastern region only). These

supplementary analyses support the spillover threshold distance and the geographic generalizability of our findings.

Control counties, defined as those situated more than 300 km away from the disaster-exposed counties, exhibiting no statistically significant excess hospitalization rates attributed to climate hazards (**Supplementary Table 6, 17 & 20**), served as candidate controls within the PSM-DID analytical framework. To ensure comparability, each disaster-exposed or neighboring county was matched with up to four control counties (**Supplementary Table 21**), accounting for geographic, demographic, socioeconomic, environmental, and health system characteristics.

Text 5 | Spillover pathways

We identified two principal spillover pathways contributing to the excess hospitalizations in neighboring regions: environmental dispersion and external patient migration. Specifically, the environmental dispersion refers to the propagation of adverse environmental changes from disaster-exposed regions into adjacent counties, directly impacting the health of local residents. Since we categorized patients by their home address, the environmental dispersion was quantified by assessing the hospitalization rate increases among residents of neighboring counties. In contrast, the patient migration refers to the inflow of patients originating from disaster-exposed counties who seek medical care in neighboring counties. Thus, the patient migration was quantified by the hospitalization rate changes of non-local residents. We incorporated these two patient sources into a unified PSM-DID analytical framework as they both represent distinct yet interconnected admission pathways through which climate hazards elevated hospitalization rates in neighboring zones.

To further elucidate the role of health system resilience in modulating these spillover pathways, we performed stratified analyses by admission source, including emergency department (ED) visits, outpatient appointments, and inter-institutional transfers. This stratification enabled a nuanced examination of the healthcare system's adaptive response and resource allocation during climate hazards, with important policy implications for health workforce planning and medical resource distribution.

Text 6 | Statistical analysis

We employed the PSM-DID framework to identify the causal relationships between climate hazards and changes in cause-specific hospitalization rates. This approach enabled us to estimate the impact of climate hazards while accounting for both observable confounders and unobservable time-invariant heterogeneity across counties.

We employed a rigorous multi-step matching protocol to identify the most comparable hazard-unaffected control counties for each hazard-affected (disaster-exposed and neighboring) county in the DID analytical framework. First, we excluded the spatially autocorrelated control counties by examining the global and local Moran's I with respect to their local cause-specific hospitalization rates:

$$\text{Global: } I_M = \frac{N_c \sum_{i=1}^{N_c} \sum_{j=1}^{N_c} w_{ij}(n_i - \bar{n})(n_j - \bar{n})}{W \sum_{i=1}^{N_c} (n_i - \bar{n})^2} \quad \text{(S4)}$$

$$\text{Local: } I_{M,i} = \frac{N_c (n_i - \bar{n}) \sum_{j=1}^{N_c} w_{ij}(n_j - \bar{n})}{W \sum_{i=1}^{N_c} (n_i - \bar{n})^2} \quad \text{(S5)}$$

where n_i represents the number of cause-specific hospitalizations at county i; $\bar{n}$ denotes the average across the nation; w_{ij} represents the element of the spatial weight matrix, determined as the reciprocal of the geophysical distance between county i and j $(i \neq j)$; and W is the summation of w_{ij}, explicitly calculated by $W = \sum_{i=1}^{N_c} \sum_{j=1}^{N_c} w_{ij}$. This procedure ensured the validity of our DID estimates by mitigating the confounding influence of spatially correlated health outcomes. Then, candidate control counties were further restricted to those sharing the same climate type[37] (Köppen-Geiger classification, **Supplementary Fig. 3**) and health system resilience levels, ensuring alignment in baseline climate condition and healthcare system (e.g., infrastructure, accessibility, expenditure, etc.) within the DID framework.

Following this pre-selection, we employed a rigorous PSM approach (1:4 nearest-neighbor matching) to identify the most comparable control counties by achieving covariates balance across multiple domains, including demographic (population density, proportion of the elderly, education attainment, gender ratio), socioeconomic (gross regional product (GRP) per capita, GRP growth rate), environmental (green coverage, urbanization), and health system (service delivery, expenditure) variables. Importantly, we excluded meteorological variables (population-weighted[38] temperature and precipitation) and air quality indicators ($PM_{2.5}$, PM_{10}, CO, NO_2, SO_2, and O_3) from the matching process, as these factors constitute potential mediators of the relationship between climate hazards and health outcomes[39]. Including mediators as matching covariates may lead to overadjustment bias[40], attenuating the estimated total impact of the climate hazard by conditioning on post-hazard variables. After matching procedures, we presented the standardized mean difference (**Supplementary Fig. 4**) and the histograms of propensity score (**Supplementary Fig. 5**) before and after PSM to demonstrate the effectiveness of matching.

Then we validated the parallel trends assumption through event-study approach[41,42] (**Supplementary Figs. 6-9**). This analysis confirmed that pre-disaster trends in hospitalization rates did not differ significantly between hazard-affected and control counties, establishing the necessary foundation for reliable DID inference. Note that we chose the event-study approach over traditional descriptive plotting because of its clear statistical advantages in assessing parallel trends. Traditional plots cannot generate confidence intervals for group differences at each time point, making it difficult to discern whether any observed deviations are statistically significant or simply due to random variation. In contrast, the event-study method estimates regression-based coefficients for each observation time relative to the reference day, providing a dynamic profile of hazard impacts. This approach enables the

construction of confidence intervals and formal statistical testing of the parallel trends assumption by evaluating whether pre-hazard coefficients are statistically indistinguishable from zero. Therefore, the event-study framework goes beyond visual inspection, offering a rigorous and transparent assessment of both pre-hazard equivalence and post-hazard impacts.

After PSM and parallel trends examination, the DID analysis was conducted using a generalized linear model with county and time fixed effects:

$$\log(Y_{it}) = \beta_0 + \beta_1 T_t + \beta_2 C_i + \beta_3 T_t C_i + \alpha_t + \lambda_i + \varepsilon_{it} \quad \text{(S6)}$$

where Y_{it} represents the cause-specific hospitalization rate in county i at time t. C_i is the binary indicator of disaster exposure (treatment) (1 for hazard-affected counties; 0 for unaffected control counties). T_t is the binary indicator of observation period (1 for post-hazard period; 0 for pre-hazard period in the observation windows). The interaction term $T_t C_i$ captures the causal effect of hazards on the log-scale. Under this log-link specification, the DID estimator β_3 represents the log-relative risk attributable to hazard exposure. Specifically, this is because under the log-link model, the expected hospitalization rate for hazard-affected counties in the post-hazard period is $Y(T=1, C=1) = \exp(\beta_0 + \beta_1 + \beta_2 + \beta_3)$, while the counterfactual hospitalization rate (what the hazard-affected counties would have experienced absent the hazard, assuming parallel trends) is $\exp(\beta_0 + \beta_1 + \beta_2)$. The ratio of actual to counterfactual gives $RR = \exp(\beta_3)$, representing the hazard impact on hospitlaization rates. The corresponding excess relative risk (ERR) is therefore given as $ERR = RR - 1 = \exp(\beta_3) - 1$.

For resilience-stratified estimates, we conducted separate DID analyses within high-resilience and low-resilience county strata. For each stratum, the DID model yielded:

$$RR_{j,h,w,r} = \exp(\beta_{3,j,h,w,r}) \quad \text{(S6a)}$$

$$ERR_{j,h,w,r} = \exp\left(\beta_{3,j,h,w,r}\right) - 1 \quad \text{(S6b)}$$

where the subscripts j, h, w, and r denote the disease type, hazard type, observation window, and resilience level, respectively. The stratum-specific estimates represent the average treatment effect across all hazard-affected counties within each resilience stratum, reflecting population-averaged hazard impacts. This stratified estimation approach is analogous to the stratified designs employed in previous disaster-health studies (e.g., stratification by social vulnerability index tertiles within hierarchical Bayesian frameworks[43]).

This PSM-DID approach effectively controls for both time-invariant county characteristics through fixed effects (λ_i) and temporal trends common to all counties

(α_t), allowing us to isolate the hazard-attributable health impacts from broader temporal shifts and persistent county-level characteristics. We repeated PSM-DID analyses across all observation periods, including warning, immediate impact (enduring period), and lag periods (stabilization-I, stabilization-II and return to normal) in both disaster-exposed and neighboring counties under all categories of climate hazards.

Note that we pool all events of the same hazard type into a single DID model. A county's treatment status varies over events: it may be treated for events where it experienced the hazard and a control for events where matched counties experienced the hazard.

We performed a set of sensitivity analyses to ensure the robustness and reliability of our primary findings. First, we employed the Monte Carlo approach (randomizing the weight matrix[15] 1,000 times) and PCA-based data-driven approach (**Supplementary Text 1.5**) to evaluate the sensitivity of our resilience assessment framework to alternative weighting schemes. This analysis assessed whether our resilience assessment framework was sensitive to the choice of weighting schemes or the intrinsic patterns in the variations of resilience indicators and pillars[18]. Second, we conducted complementary tertile-based resilience classifications (low, moderate, and high) to examine the resilience gradient effects and validate the robustness of our policy recommendations. Third, we examined the robustness of health impact estimates to model specification by re-estimating the DID models with county-level random effects only. This comparison allowed us to gauge the sensitivity of our results to different assumptions regarding the variance structure and to minimize potential convergence or precision issues associated with complex random effects. Fourth, we conducted sensitivity analyses using DID without prior PSM to examine the potential regression-to-the-mean effects that might arise from county selection controls with atypically similar baseline values[44,45]. Fifth, we repeated the PSM-DID analysis excluding the pandemic period. Finally, we generated ALE plots (**Supplementary Figs. 17 & 18**) to cross-validate policy insights derived from the PDPs, thereby strengthening the interpretability and reliability of our adaptation recommendations.

Text 7 | Hospitalization risk disparities between resilience strata

The Intergovernmental Panel on Climate Change (IPCC) conceptualized resilience as a system's capacity to cope with and recover from hazardous events or disruptions while maintaining essential functions through responsive reorganization[46]. We followed this concept and operationalized the hospitalization risk disparities between resilience strata during and after disasters as metrics reflecting the potential role of health system resilience. Specifically, we defined two operational metrics – the hazard active period metric (Re) and the post-hazard period metric (Rc) – capturing the percentage reduction in hazard-attributable hospitalization relative changes in high-resilience regions relative to low-resilience regions.

Taking the health impacts in low-resilience regions as reference, the hazard-active period metric (Re) of high-resilience counties is quantified by the degree of

hospitalization change reduction (i.e., reflected by the excess relative risk, $ERR = RR - 1$, with RR being the relative risk calculated by the PSM-DID analytical framework) during disasters:

$$Re_{j,h} = \left(1 - \frac{ERR_{high,j,h}}{ERR_{low,j,h}}\right) \times 100\% = \frac{ERR_{low,j,h} - ERR_{high,j,h}}{ERR_{low,j,h}} \times 100\% \quad \text{(S7)}$$

where subscripts j and h denotes the disease and hazard types, respectively. Given the disease-specific incidence rates[47] (I_j), the overall hazard-active period metric for high-resilience region is therefore given by:

$$\widehat{Re}_h = \frac{\sum_{j=1}^{N_D} Re_{j,h} I_j ERR_{low,j,h}}{\sum_{j=1}^{N_D} I_j ERR_{low,j,h}} \quad \text{(S8)}$$

where N_D is the total number of diseases. $I_j ERR_{low,j,h}$ represents the j disease hospitalization rate changes in low-resilience regions under h type disasters.

Similarly, we measured the post-hazard period metric (Rc) as the percentage reduction of accumulated excess relative risk in high-resilience regions during the lag periods:

$$Rc_{j,h} = \left(1 - \frac{\sum_{k=1}^{N_w} ERR_{high,j,h,k} D_{high,h,k}}{\sum_{k=1}^{N_w} ERR_{low,j,h,k} D_{low,h,k}}\right) \times 100\% \quad \text{(S9)}$$

where the subscript k represents the observation window; N_w is the total number of observation windows in the lag period; and D is the length of specific observation window. Since we set equal-length observation window for stabilization-I, stabilization-II and return to normal periods, **Eq. S9** was simplified as:

$$\begin{aligned} Rc_{j,h} &= \left(1 - \frac{\sum_{k=1}^{N_w} ERR_{high,j,h,k}}{\sum_{k=1}^{N_w} ERR_{low,j,h,k}}\right) \times 100\% \\ &= \frac{\sum_{k=1}^{N_w} ERR_{low,j,h,k} - \sum_{k=1}^{N_w} ERR_{high,j,h,k}}{\sum_{k=1}^{N_w} ERR_{low,j,h,k}} \times 100\% \end{aligned} \quad \text{(S10)}$$

Given the disease-specific incidence rates, we thereby computed the overall post-hazard period metric for high-resilience regions:

$$\widehat{Rc}_h = \frac{\sum_{j=1}^{N_D} Re_{j,h} I_j R_{low,j,h,k}}{\sum_{j=1}^{N_D} I_j R_{low,j,h,k}} \quad \text{(S11)}$$

where $R_{low,j,h,k} = \sum_{k=1}^{N_w} ERR_{low,j,h,k}$ is the cumulated excess relative risk for j disease hospitalizations triggered by the h type hazard.

Notably, it is important to note the conceptual distinction between our static resilience metrics (**Eq. S1-S3**) and the hazard-active/post-hazard period metrics (**Eq. S7-S11**). Static metrics quantify baseline health system capacity prior to hazard exposure, representing inherent structural and functional preparedness. The hazard-active and post-hazard period metrics, in contrast, measure observable hospitalization patterns during and after hazard events, capturing the degree to which regions with different resilience levels exhibit differential hospitalization changes under climate stress. Our analytical framework does not assume static metrics deterministically predict hospitalization outcomes; rather, we empirically test this relationship through stratified study design while controlling for observable confounders.

Interpretation of between-strata differences: Given our stratified PSM-DID design, β_3 estimated within high-resilience counties ($\beta_{3,\mathrm{high-resilience}}$) and low-resilience counties ($\beta_{3,\mathrm{low-resilience}}$) represent the causal effect of hazards on hospitalization rates for each resilience level, conditional on observed covariates being balanced. The hazard-active and post-hazard period metrics (**Eq. S7-S11**) isolate the differential hospitalization changes attributable to differences in composite resilience capacity rather than any single resilience component. This interpretation is valid because: (i) both resilience strata experienced identical hazard exposure; (ii) both strata were balanced on baseline confounders through PSM; (iii) the systematic difference between strata is resilience level itself; and (iv) resilience components (infrastructure, workforce, financing, etc.) are constituent elements of the stratification variable (resilience), not confounders to be controlled.

This approach aligns with WHO's operational definition of health system resilience[2], which encompasses structural, economic, and social determinants as integral components of a system's capacity to prepare for, respond to, and recover from health crises. Our research question "Do health systems with higher composite resilience were associated with smaller hospitalization relative changes during climate hazards?" is addressed by comparing hospitalization outcomes across resilience strata under matched baseline conditions.

Text 8 | Data-driven adaptation policy implications

Regarding the adaptation policies, while the conceptual importance of health system resilience in hazard mitigation is widely recognized[3], critical questions regarding the efficiency of health-oriented investments in public health benefits and whether investments in these areas yield diminishing health returns beyond certain thresholds remain largely underexplored, limiting the actionable interventions in healthcare systems. To address this knowledge gap, we first quantified the county-specific hospitalization risk disparities under all types of hazards (**Eq. S8 & S11**). Specifically, while the ERR values presented in **Fig. 3** represent the average effect for all counties

within the same resilience stratum (derived from PSM-DID analysis), the hazard-active and post-hazard period metrics used in the random forest analysis were computed using county-specific ERR values for each hazard-affected county at different time periods. These county-specific measures capture the observed hospitalization patterns of each individual county during and after each hazard exposure, providing the necessary variation in these metrics for meaningful random forest analysis. By correlating these county-specific health consequences (indicated by hazard-active and post-hazard period metrics) with corresponding resilience metric attributes, we were able to build the random forest model and construct the partial dependence plots (PDPs) for policy implications.

We then conducted two complementary random forest (RF) analyses to comprehensively characterize resilience determinants and their health impacts. First, to identify the key determinants of overall static resilience score, we employed RF regression models with the overall static resilience score (**Eq. S1-S3**) as the outcome variable and individual resilience indicators or resilience pillars as predictors (**Fig. 1d & Supplementary Fig. 12a**). This analysis quantified which fundamental indicators/pillars are the primary determinants of overall static resilience across counties, providing critical guidance for resilience assessment and intervention prioritization, particularly in data-limited settings where comprehensive health surveillance data may be unavailable for back analysis.

Second, we utilized the RF approach to determine the relative importance[48] (i.e., percentage increase in mean square error, MSE) of resilience metrics on overall hospitalization reduction (i.e., hazard-active and post-hazard period metrics) during hazards. Here the RF algorithm was selected for its robust handling of complex, non-linear associations and inherent resistance to outliers. Model hyperparameters, including the number of trees, the number of variables randomly sampled at each split, and the minimum node size were optimized through K-fold cross-validation[49]. This approach maximized predictive accuracy while minimizing the risk of overfitting, thereby ensuring that our RF model captured genuine patterns linking resilience metrics and health outcomes rather than statistical noise.

Subsequently, we constructed partial dependence plots[49] (PDPs) to visualize and interpret the marginal association of resilience metrics with hazard-active and post-hazard period metrics under disasters. These PDPs had essential policy implications as they quantified the potential effectiveness (public health gains, expressed by the percentage reduction in cause-specific hospitalization changes, i.e., hazard-active and post-hazard period metrics) of healthcare system-oriented investments during climate hazards. Additionally, two-dimensional PDPs revealed potential synergistic effects among multiple resilience components, indicating that the combined implementation of strategies can yield health benefits greater than the sum of their individual effects. Moreover, PDPs offered intuitive judgments of potential thresholds or tipping points where additional investments in specific health system components yielded diminishing public health returns, thereby identifying the most effective enhancement pathways for adaptation intervention.

Finally, to validate our findings, we complemented the PDPs with accumulated local effects[50] (ALE) plots (**Supplementary Figs. 17 & 18**), which provided a more robust representation of public health benefits in the presence of correlated health system components. This cross-validation approach strengthened the robustness of our policy recommendations and provided a solid empirical basis for prioritizing health system investments in hazard adaptation planning.

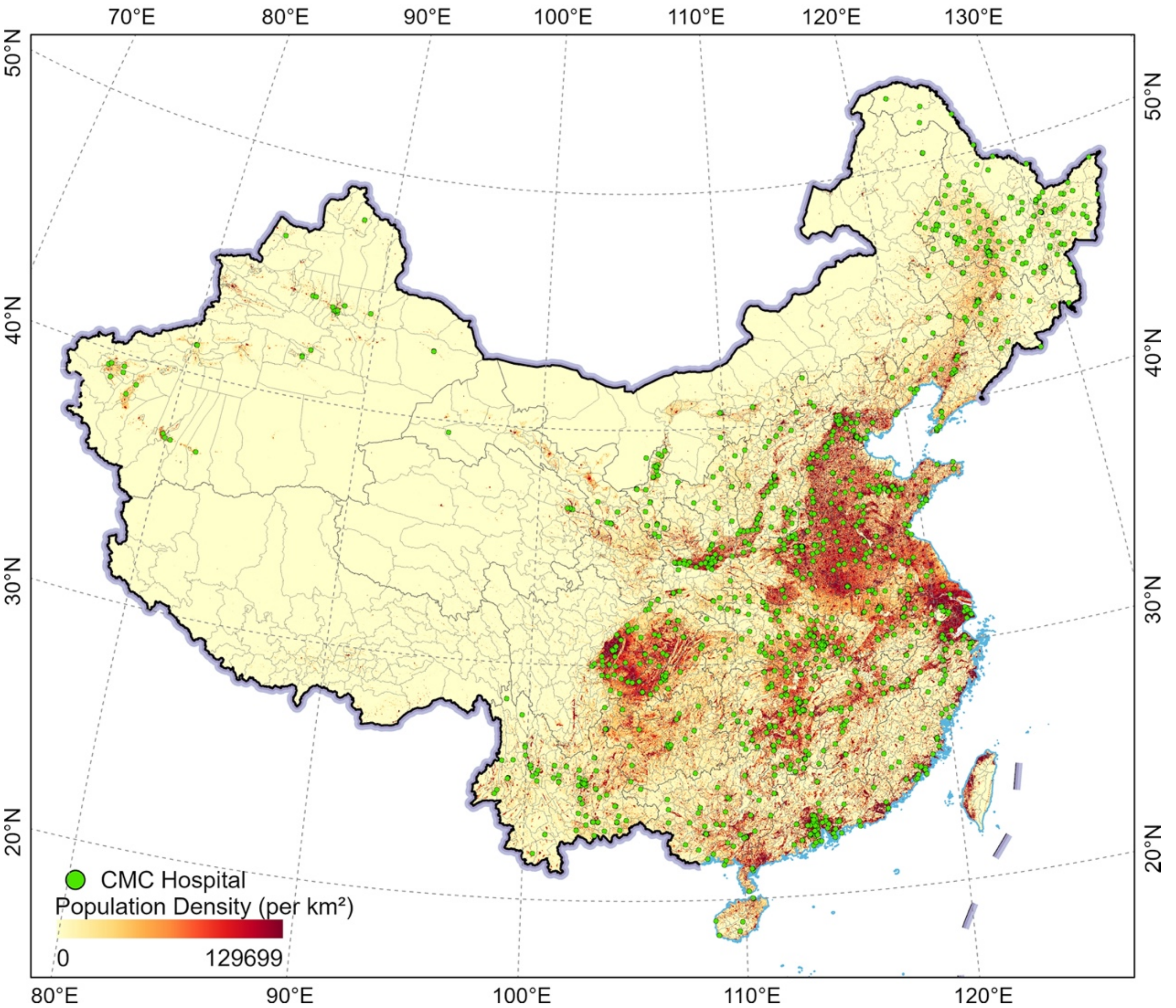

Fig. 1 | CMC Hospital Network and population density distribution in China.
Note: The population density data in 2016, used for demonstration, is obtained from the WorldPop[34].

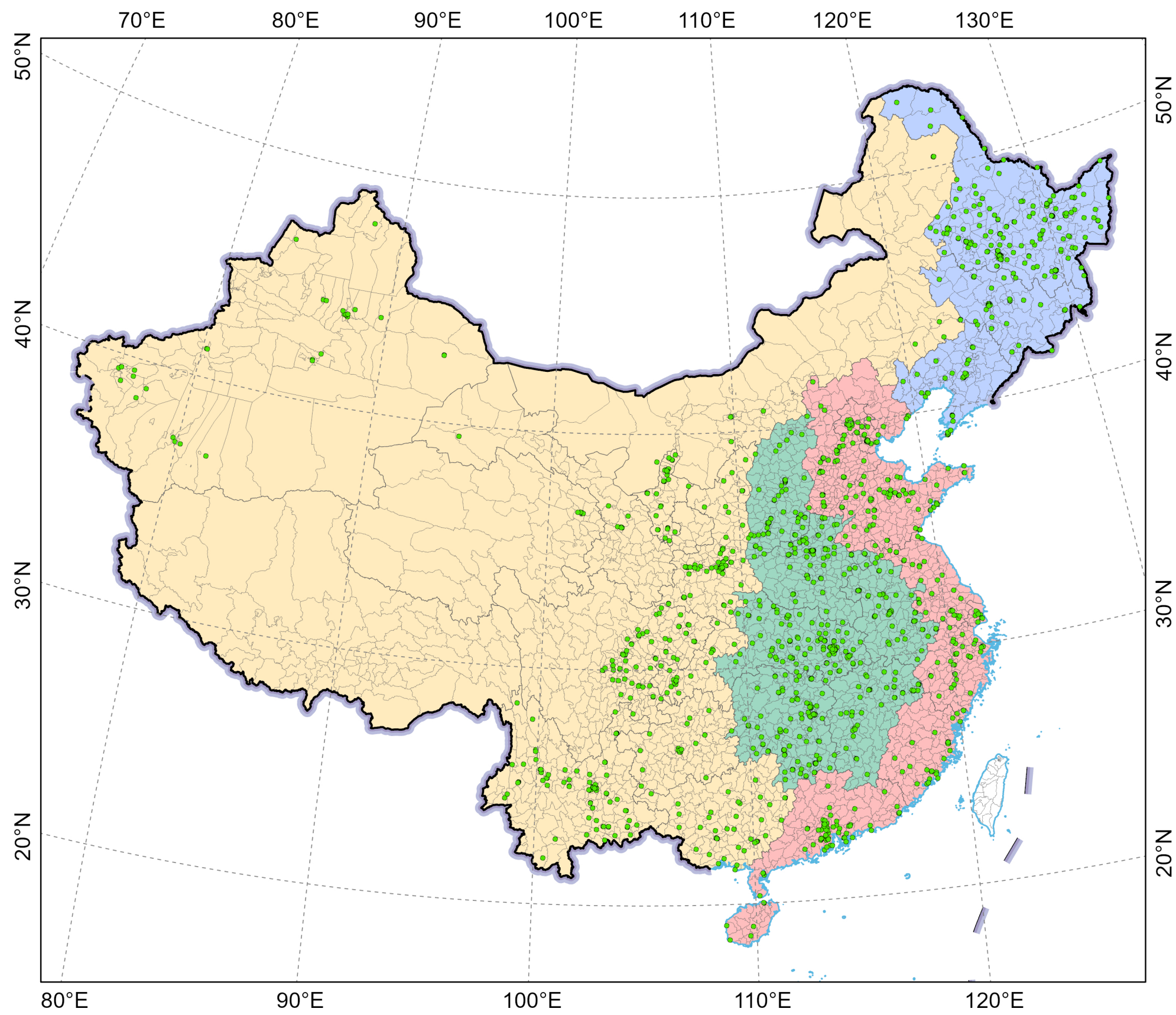

Fig. 2 | CMC Hospital Network covered and uncovered counties in subdivided regions in China. Note: the subregions, including central, eastern, northeastern, and western, are classified by the National Bureau of Statistics[51] based on regional socioeconomic development and geographic environmental conditions.

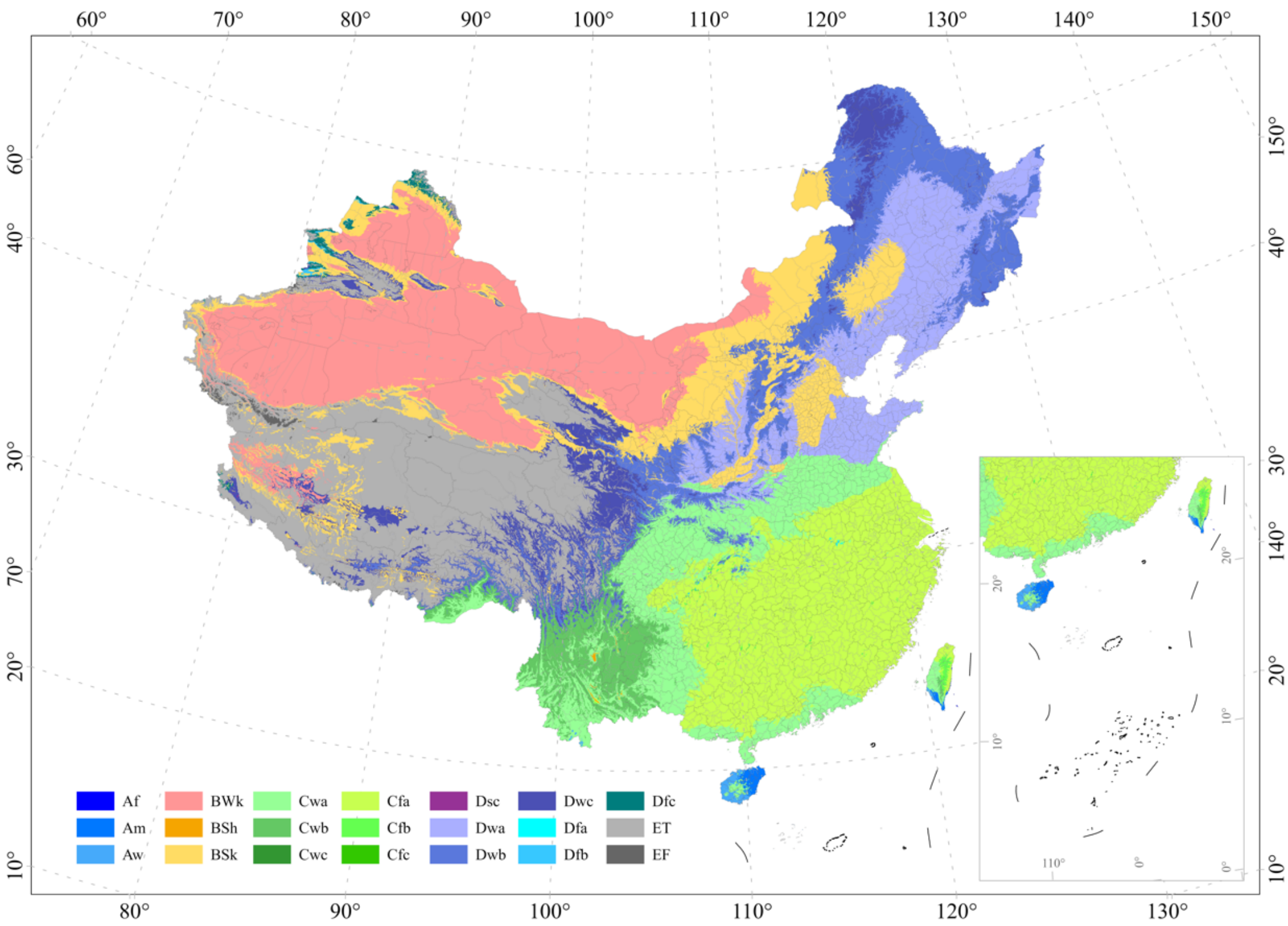

Fig. 3 | High resolution (1 km) local climate type in China using Köppen-Geiger classification. Af – tropical, rainforest; Am – tropical, monsoon; Aw – Tropical, savannah; BWk – Arid, desert, cold; BSh – Arid, steppe, hot; BSk – Arid, steppe, cold; Cwa – temperate, dry winter, hot summer; Cwb – temperate, dry winter, warm summer; Cwc – temperate, dry winter, cold summer; Cfa – temperate, no dry season, hot summer; Cfb – temperate, no dry season, warm summer; Cfc – temperate, no dry season, cold summer; Dsc – cold, dry summer, cold summer; Dwa – cold, dry winter, hot summer; Dwb – cold, dry winter, warm summer; Dwc – cold, dry winter, cold summer; Dfa – cold, no dry season, hot summer; Dfb – cold, no dry season, warm summer; Dfc – cold, no dry season, cold summer; ET – polar, tundra; EF – polar, forest. Data was obtained from the GloH2O databse[37].

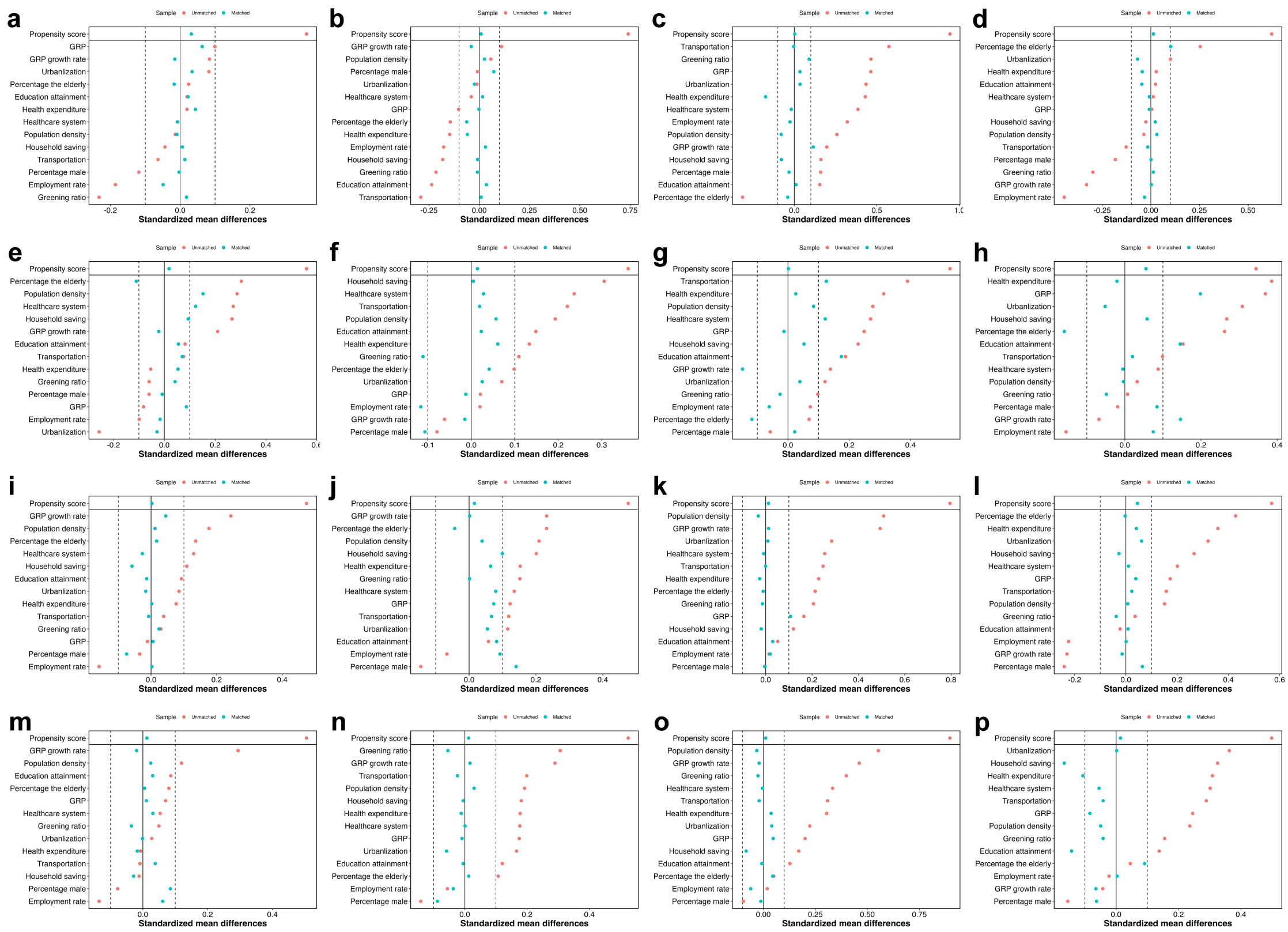

Fig. 4 | Standardized mean differences of covariates before and after propensity score matching (PSM). Disaster-exposed region: **a,** storms **b,** floods **c,** tropical cyclones **d,** blizzard/winter storms; N-I region (0-100 km neighboring regions): **e,** storms **f,** floods **g,** tropical cyclones **h,** blizzard/winter storms; N-II region (100-200 km neighboring regions): **i,** storms **j,** floods **k,** tropical cyclones **l,** blizzard/winter storms; N-III region (200-300 km neighboring regions): **m,** storms **n,** floods **o,** tropical cyclones **p,** blizzard/winter storms. The red and green dots represent the standard mean differences before and after PSM, respectively. The vertical dash lines represent the 10% interval around 0.

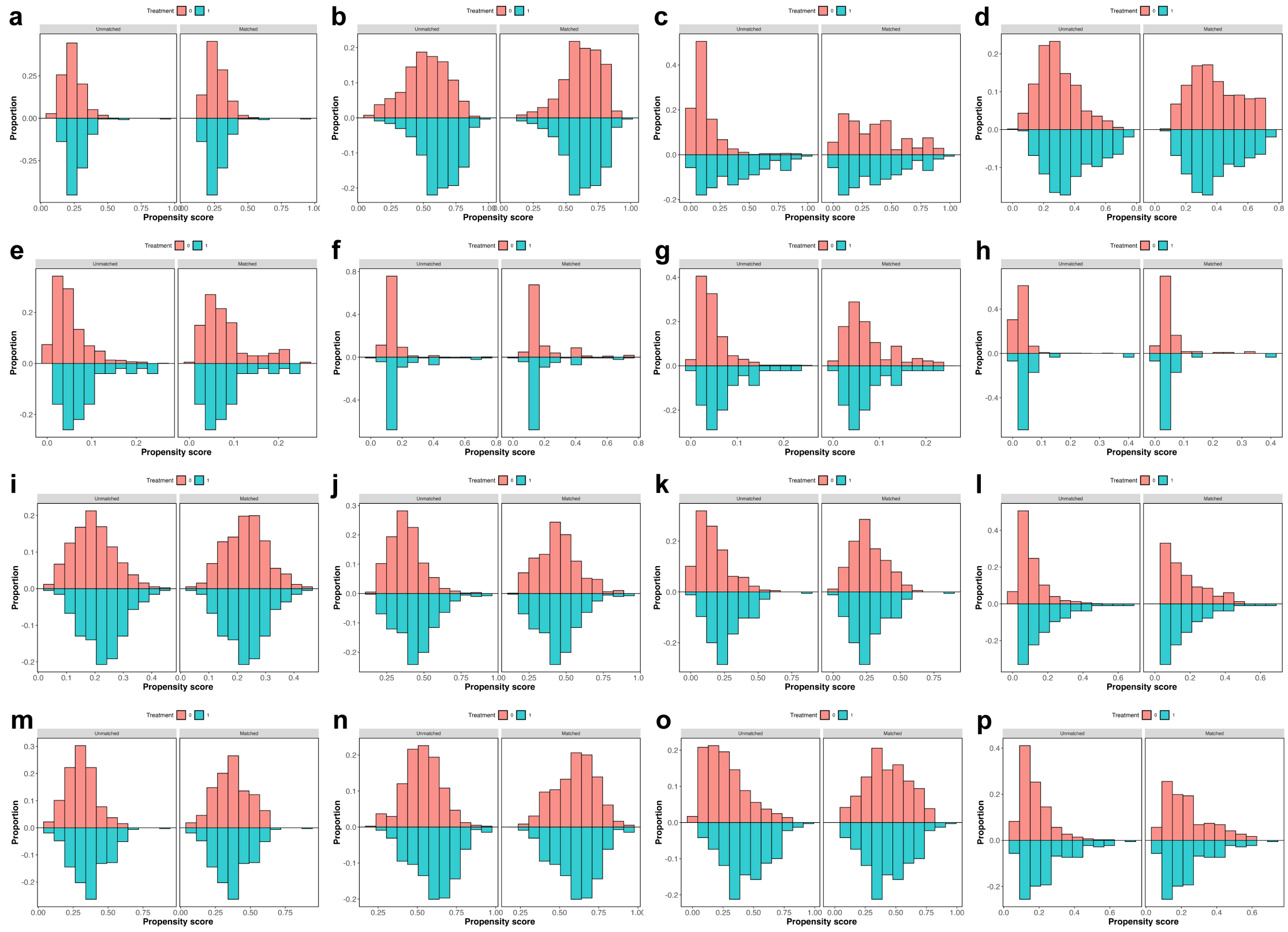

Fig. 5 | Histograms of propensity score before and after PSM. Disaster-exposed region: **a,** storms **b,** floods **c,** tropical cyclones **d,** blizzard/winter storms; N-I region (0-100 km neighboring regions): **e,** storms **f,** floods **g,** tropical cyclones **h,** blizzard/winter storms; N-II region (100-200 km neighboring regions): **i,** storms **j,** floods **k,** tropical cyclones **l,** blizzard/winter storms; N-III region (200-300 km neighboring regions): **m,** storms **n,** floods **o,** tropical cyclones **p,** blizzard/winter storms. Note: the left-hand side subfigure represents the propensity score distribution before PSM, while the right-hand side figure represents the propensity score distribution after PSM.

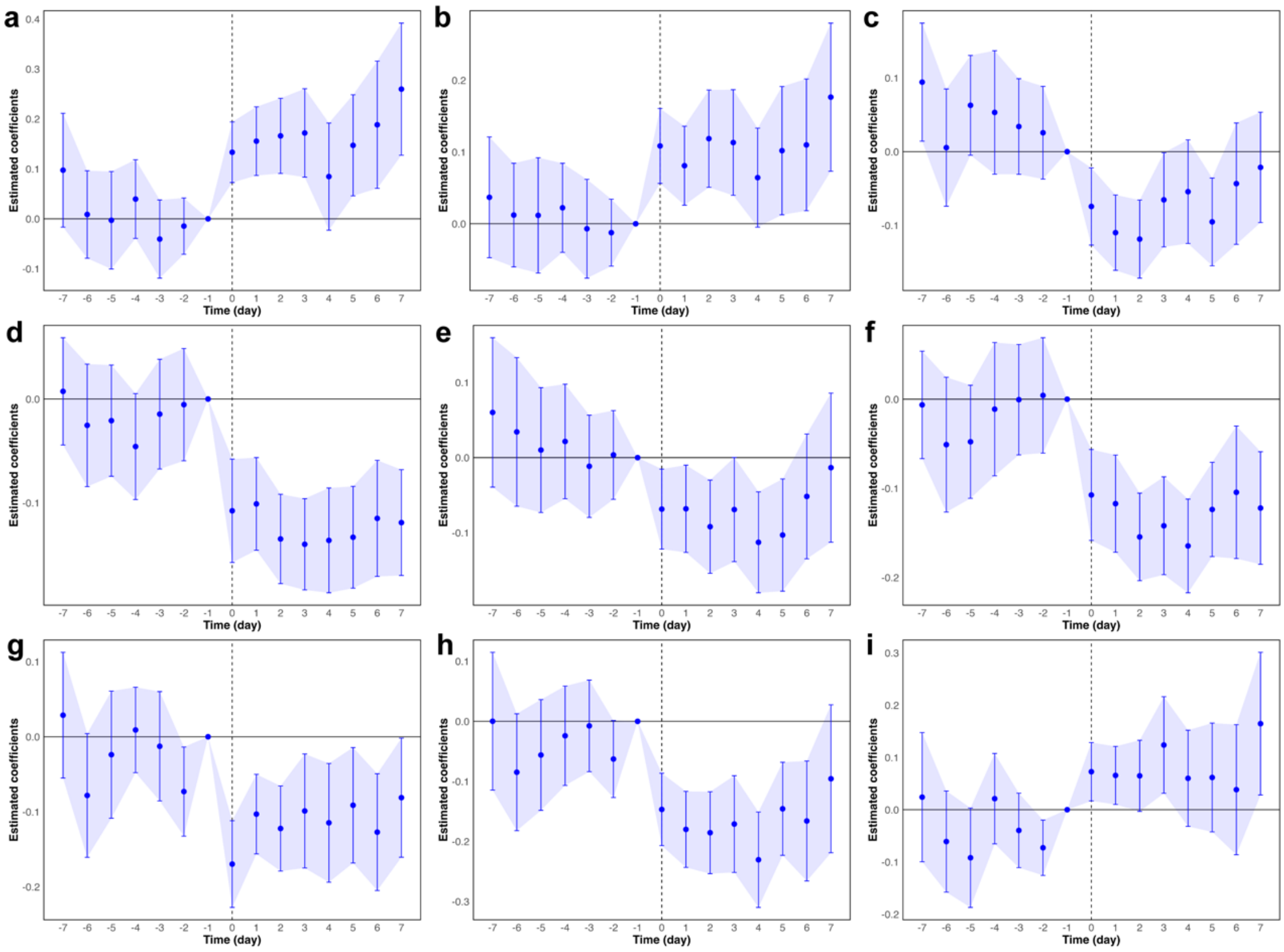

Fig. 6 | Parallel trend tests for climate-sensitive diseases under floods. a, respiratory **b,** injuries **c,** infectious **d,** mental **e,** neoplasms **f,** endocrine **g,** neurology **h,** genitourinary and **i,** cardiovascular disease. Day 0 indicates the hazard onset. The negative days represent (part of) the pre-hazard days in the difference-in-differences (DID) analysis. The one-week warning period is excluded. The vertical axis represents the estimated DID coefficients for each day relative to the reference (day -1). The coefficients for pre-hazard days (negative values) provide a visual and statistical test of the parallel trends assumption: if the treatment (hazard-affected) and the control (non-affected) groups exhibit statistically indistinguishable trends prior to the hazard onset, this supports the reliability of the DID design. Across all panels, the pre-hazard coefficients were generally centered around zero and not statistically significant, indicating no systematic differences in cause-specific hospitalization rates between the groups before the hazard. This evidence supports the parallel trends assumption and suggests that the captured post-hazard changes in hospitalization rates can be attributed to the climate hazard rather than to pre-existing differences between the groups.

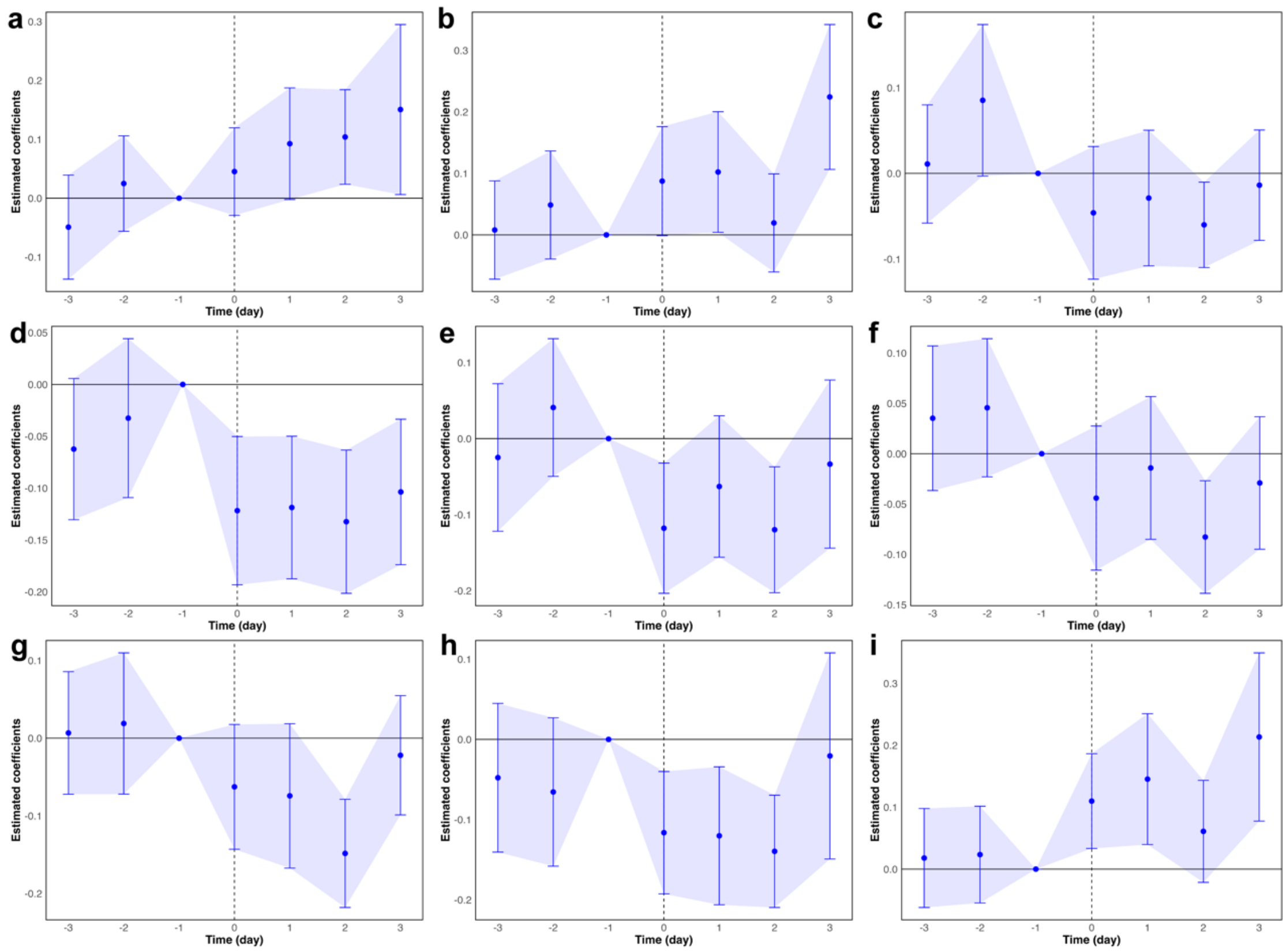

Fig. 7 | Parallel trend tests for climate-sensitive diseases under storms. a, respiratory **b,** injuries **c,** infectious **d,** mental **e,** neoplasms **f,** endocrine **g,** neurology **h,** genitourinary and **i,** cardiovascular disease. Day 0 indicates the hazard onset. The negative days represent (part of) the pre-hazard days in the difference-in-differences (DID) analysis. The one-week warning period is excluded. The vertical axis represents the estimated DID coefficients for each day relative to the reference (day -1).

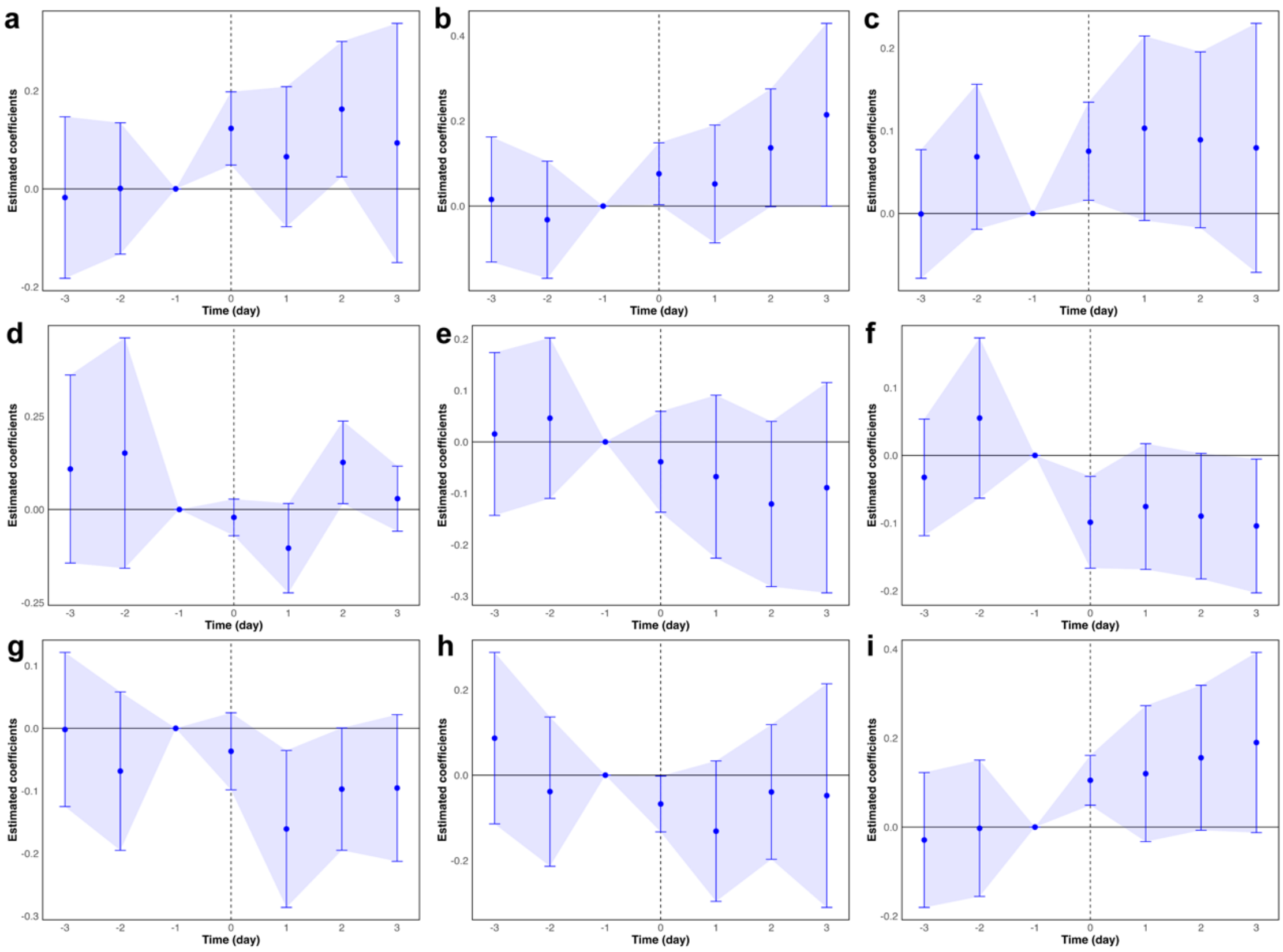

Fig. 8 | Parallel trend tests for climate-sensitive diseases under tropical cyclones. **a,** respiratory **b,** injuries **c,** infectious **d,** mental **e,** neoplasms **f,** endocrine **g,** neurology **h,** genitourinary and **i,** cardiovascular disease. Day 0 indicates the hazard onset. The negative days represent (part of) the pre-hazard days in the difference-in-differences (DID) analysis. The one-week warning period is excluded. The vertical axis represents the estimated DID coefficients for each day relative to the reference (day -1).

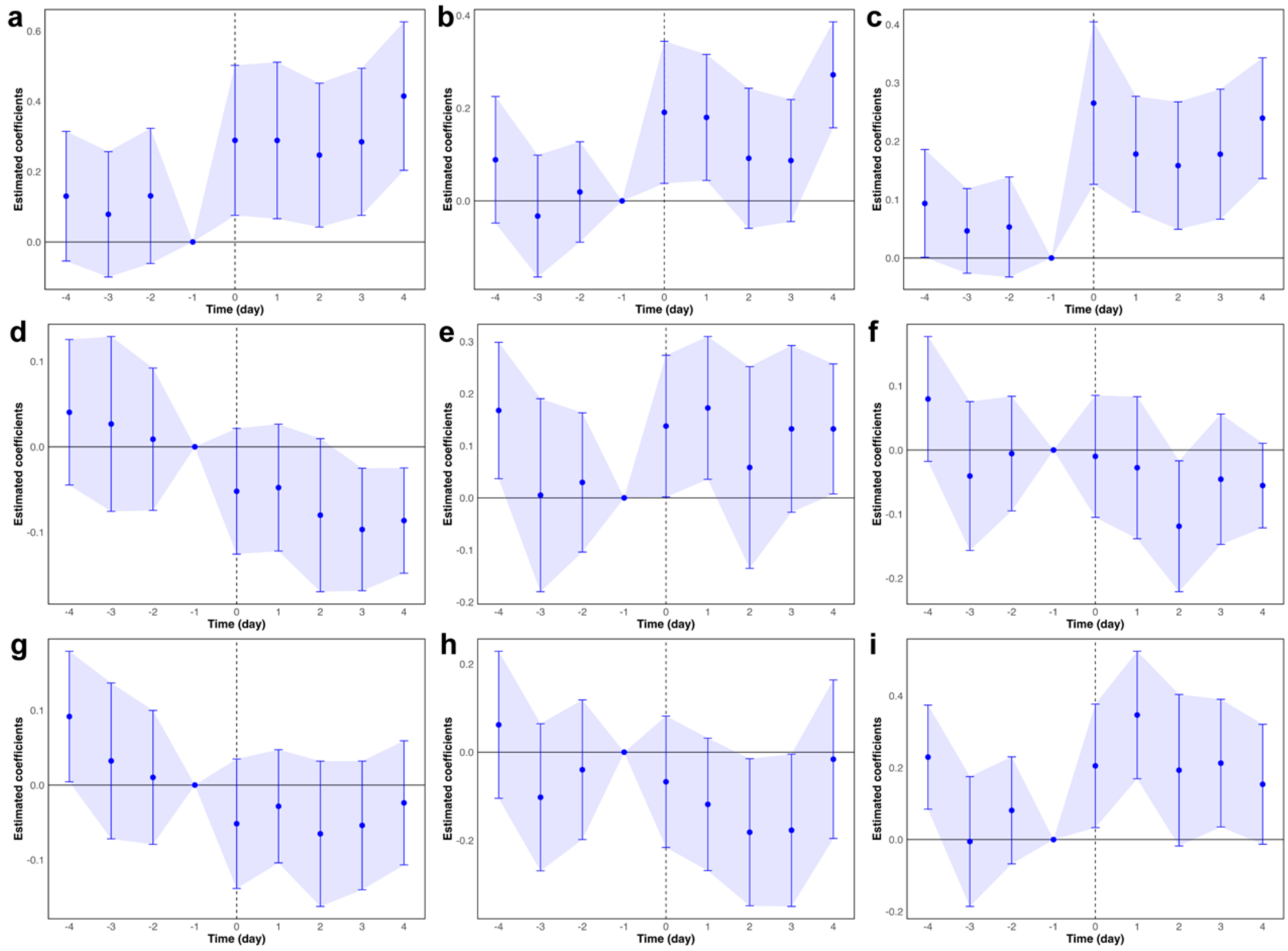

Fig. 9 | Parallel trend tests for climate-sensitive diseases under blizzard/winter storms. a, respiratory **b,** injuries **c,** infectious **d,** mental **e,** neoplasms **f,** endocrine **g,** neurology **h,** genitourinary and **i,** cardiovascular disease. Day 0 indicates the hazard onset. The negative days represent (part of) the pre-hazard days in the difference-in-differences (DID) analysis. The one-week warning period is excluded. The vertical axis represents the estimated DID coefficients for each day relative to the reference (day -1).

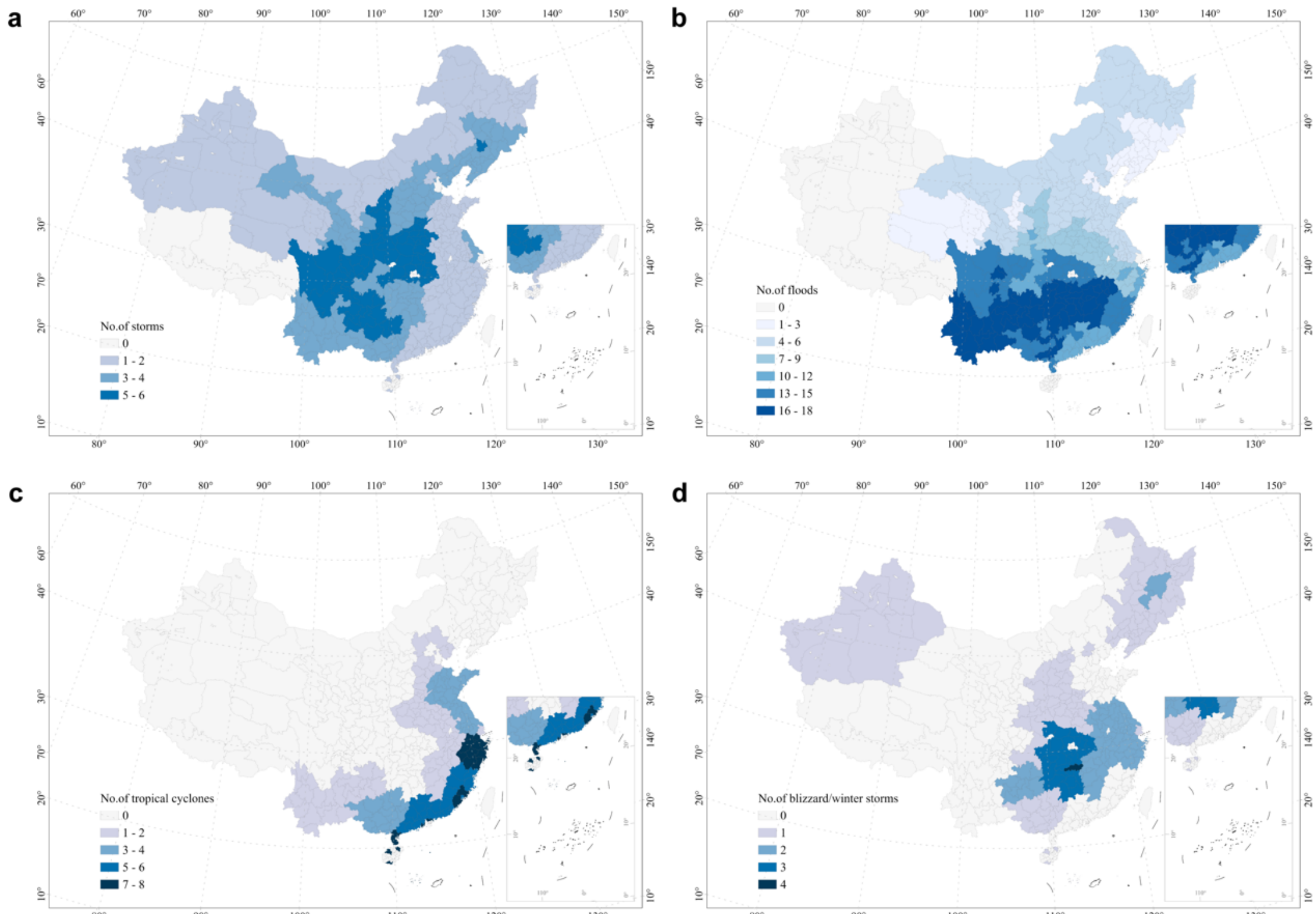

Fig. 10 | Climate hazards in China from 2016 to 2023. a, storms **b,** floods **c,** tropical cyclones **d,** blizzard/winter storms.

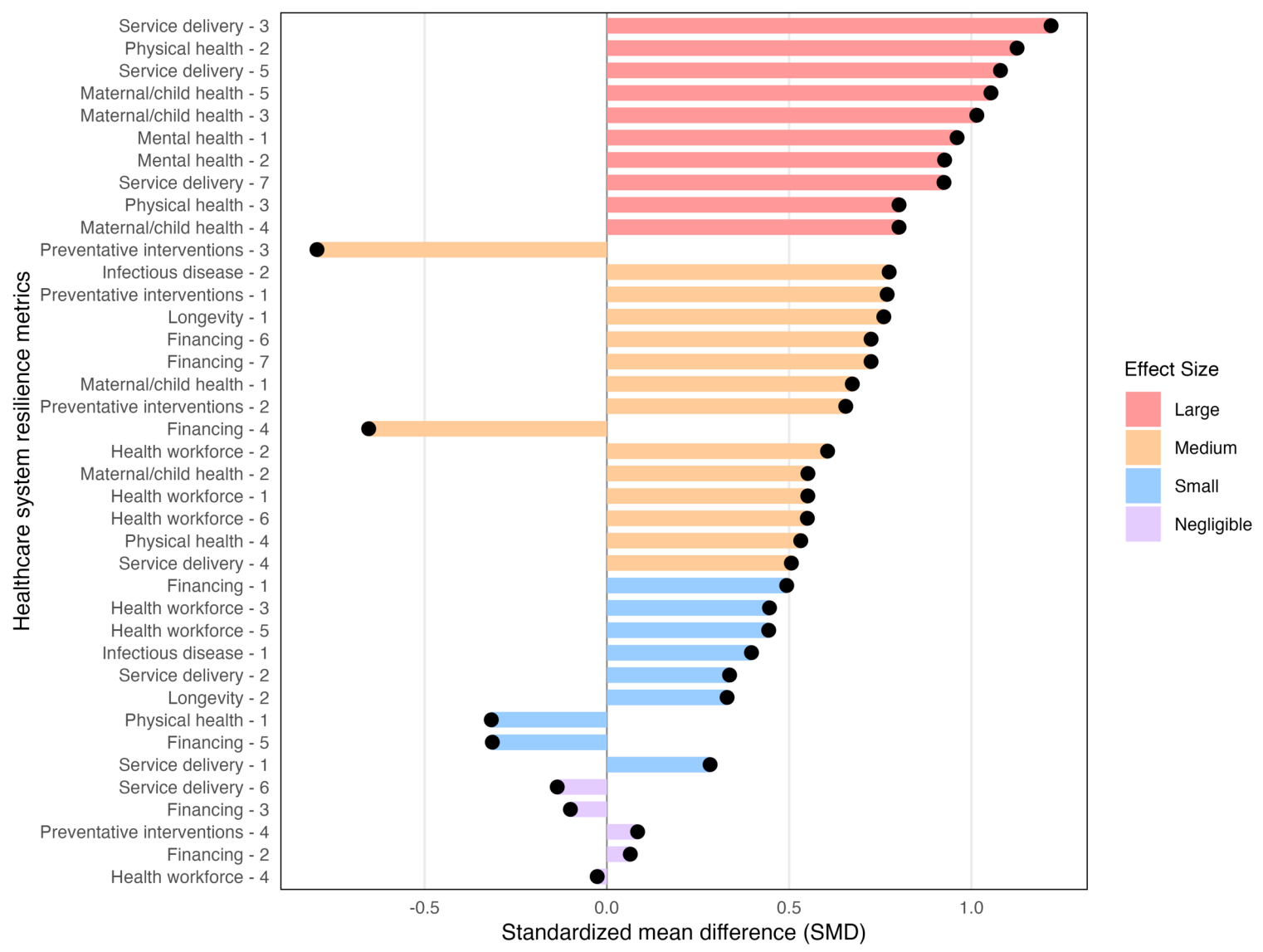

Fig. 11 | Comparison of health system resilience metrics between low and high-resilience regions, indicated by the standardized mean differences (SMD). Service delivery: 1 - Number of Number of hospitals (per 1,000 persons); 2 - Number of inpatient beds; 3 - Average bed occupancy ratio; 4 - Average number of health workers per inpatient bed; 5 - Outpatient department, primary health care and emergency; 6 - department service utilization rate; 7 - Average length of stay (ALOS) at hospital or health facility; 8 - Average number of consultations per capita per year; Health workforce: 1 - Number of Number of hospitals (per 1,000 persons); 2 - Number of community health workers per 1,000 population; 3 - Average number of staff, by occupation in primary care facilities and hospitals; 4 - Rural-to-urban distribution ratio of health workers; 5 - Percentage of health workers that received training and qualifications from an accredited educational or training institution or body; 6 - Percentage of births attended by skilled personnel (doctors, nurses, midwives); Preventative interventions: 1 - Consultations on health education technology and policy recommendations; 2 - Health education activities; 3 - Fluoride (water type) prevention and control; 4 - Iodine deficiency disease prevention and treatment; Longevity: 1 - Life expectancy; 2 - Mortality rate (per 10,000 persons per year); Financing: 1 - Government financial allocation for healthcare; 2 - Healthcare expenditure per 10,000 persons; 3 - General government expenditure on primary health care (PHC) as a share of general government health expenditure; 4 - Price index for medicine and health products; 5 - Price index for medical service; 6 - Healthcare insurance cover rate; 7 - Participation rate in pension insurance; Physical health: 1 - ED visit rate (per 100 hospitals); 2 - Physician workload; 3 - Food safety and foodborne disease reports (per year); 4 - General practice outpatient and emergency visits rate; Mental health: 1 - Mental disorder hospitalization incidence rate; 2 - Psychiatric outpatient and emergency visits rate; Maternal/child health: 1 - Maternal healthcare system management rate (%); 2 - Maternal healthcare antenatal examination rate (%); 3 - Maternal healthcare postpartum visit rate (%); 4 - Neonatal visit rate due to vaccination received (%); 5 - Healthcare management rate for children under 7-year-old (%); Infectious disease: 1 - Number of beds designated for

isolation of infectious diseases at the healthcare facility - incidence rate (per 100,000 persons per year); 2 - Number of beds designated for isolation of infectious diseases at the healthcare facility - mortality rate (per 100,000 persons per year).

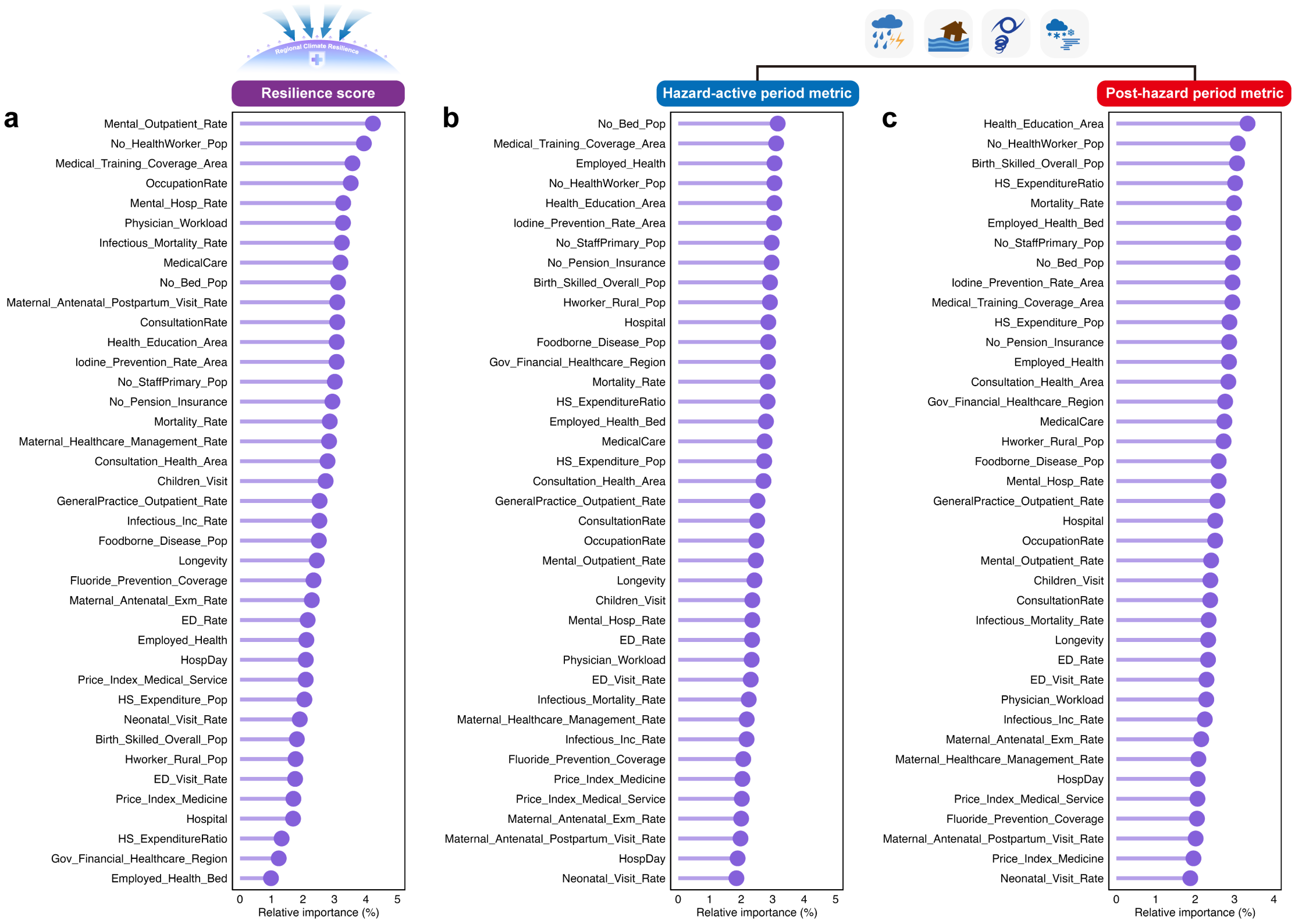

Fig. 12 | Relative importance of health system resilience metrics on overall resilience score and hospitalization risk disparities during and after climate hazards. a, overall resilience score **b,** hazard-active period metric **c,** post-hazard period metric.

Disease	Hazard-active period metric (%)				Post-hazard period metric (%)			
	Storm	Flood	Tropical cyclone	Blizzard/ Winter storm	Storm	Flood	Tropical cyclone	Blizzard/ Winter storm
Overall	30.03	19.48	26.06	28.50	31.08	29.30	27.80	27.07
Respiratory	40.56	35.74	27.96	19.71	15.88	27.78	36.04	26.44
Injuries	29.08	24.75	17.15	17.65	64.08	54.15	37.21	25.16
Infectious	22.93	20.85	37.00	37.02	7.55	22.28	22.99	20.73
Mental	36.84	30.44	22.45	43.31	25.41	25.56	26.87	25.56
Neoplasms	33.90	29.46	23.89	44.92	78.32	23.65	29.66	15.43
Endocrine	26.77	21.31	14.72	41.07	57.94	21.29	25.03	14.63
Neurology	23.05	14.32	41.10	25.31	14.95	28.02	25.81	23.13
Genitourinary	42.26	12.96	15.06	22.82	32.95	26.71	29.57	43.45
CVD	30.31	15.35	20.84	26.79	40.21	29.26	22.78	23.30

Fig. 13 | Hospitalization risk disparities between high- and low-resilience regions during hazard-active and post-hazard periods. The percentage values indicate, taking the low-resilience regions as reference, the extent to which high-resilience regions exhibited reduced hospitalization changes during climate hazards, based on the health data from the CMC Hospital Network and disaster records documented by the EM-DAT. Specifically, the hazard-active period metric represents the percentage reduction in excess hospitalization risk during the hazard-active period, whereas the post-hazard period metric represents the proportion of of excess hospitalization risk reduced in high-resilience regions relative to low-resilience regions during the post-hazard periods.

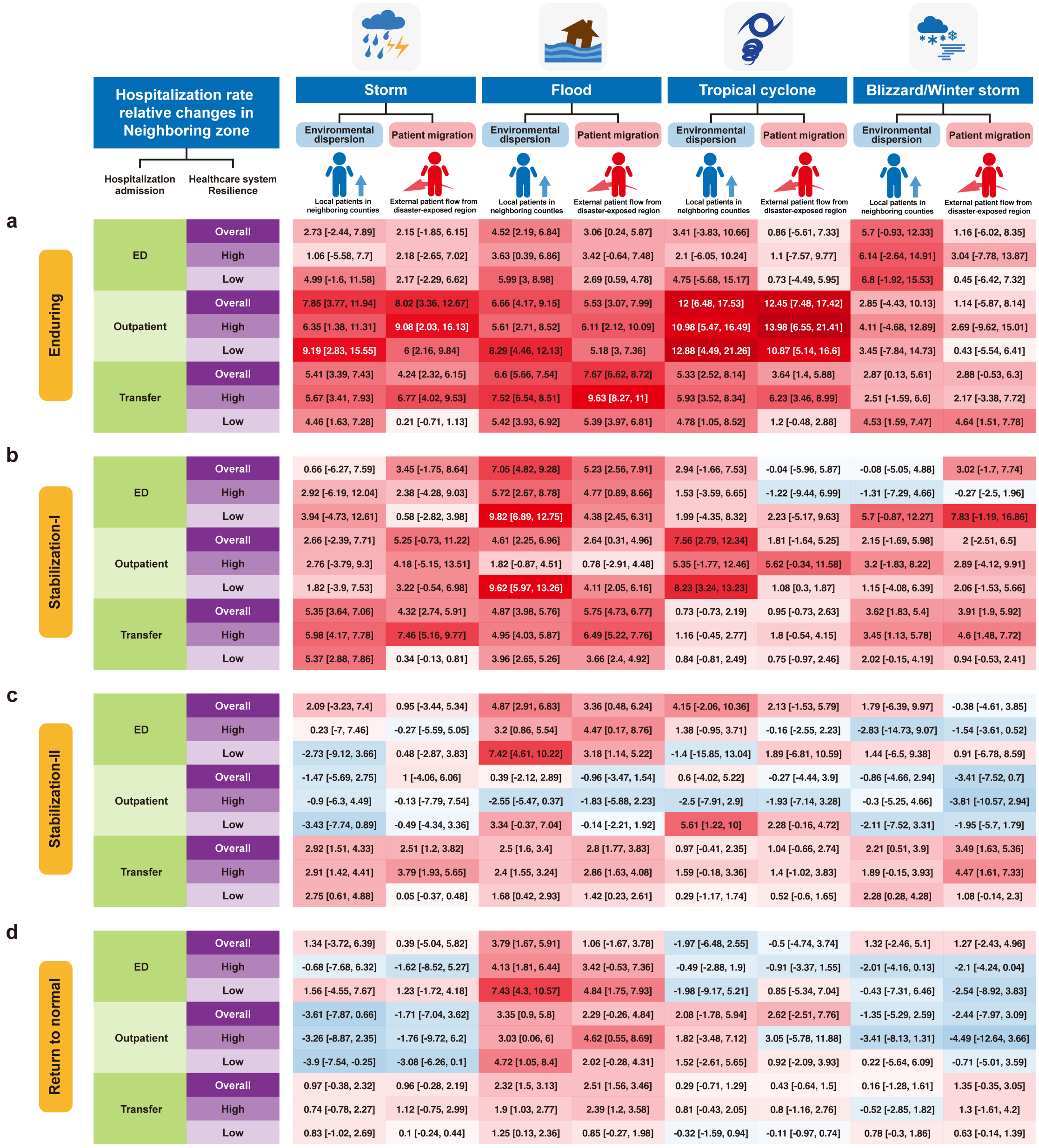

Fig. 14 | Stratification analysis on the spillover pathways in 0 – 200 km neighboring counties. The environmental dispersion was estimated by the relative changes of hospitalization rates attributable to the local residents living in neighboring counties, whereas the patient migration was quantified by the relative changes of hospitalization rates attributable to the non-local patients from the disaster-exposed counties. **a,** enduring **b,** stabilization-I **c,** stabilization-II **d,** return to normal. The values represent the excess relative risks (i.e., $ERR = RR - 1$, with RR being the relative risk directly obtained from the PSM-DID analytical framework). Note that the hospitalization admissions (from ED, outpatient visits, and transfer from other institutions) were all registered in neighboring counties. Abbreviation: ED – emergency department.

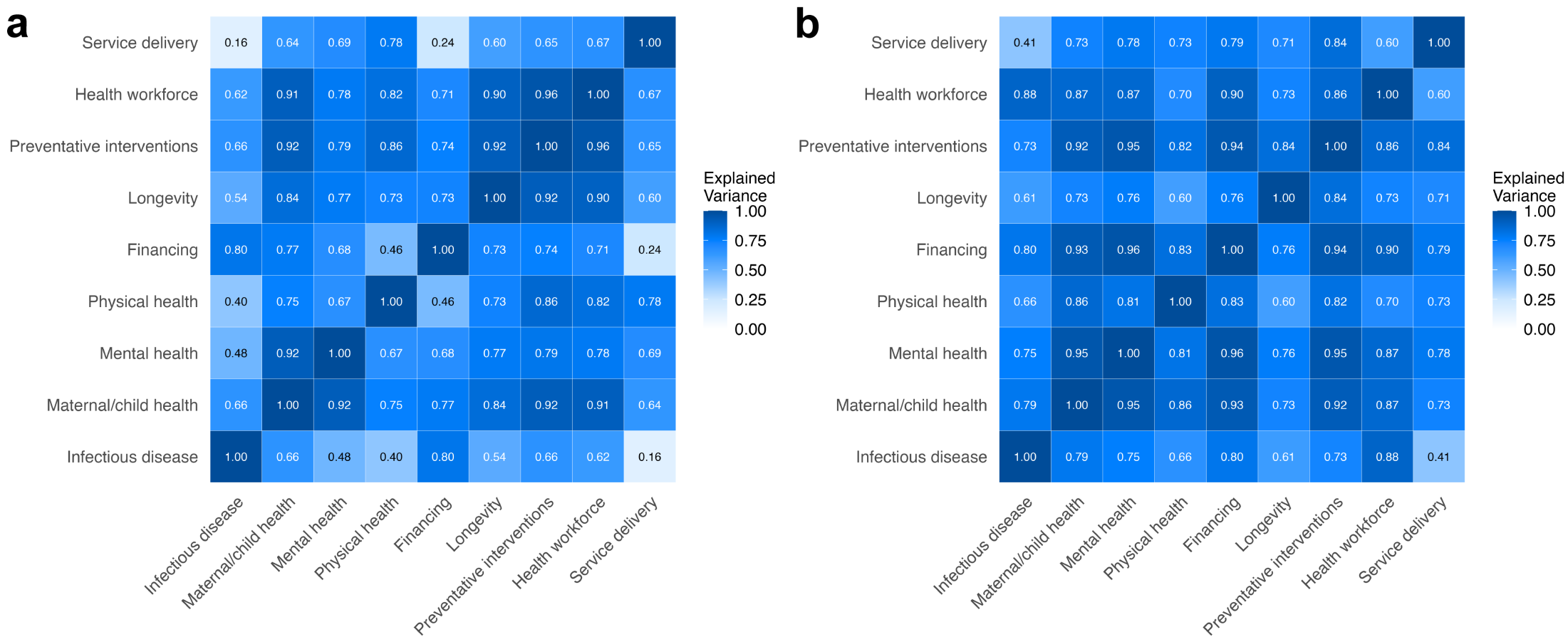

Fig. 15 | Heatmap of correlation strength between health system resilience metrics during the hazard-active and post-hazard periods. a, enduring period **b,** post-hazard periods (stabilization-I, stabilization-II, and return to normal).

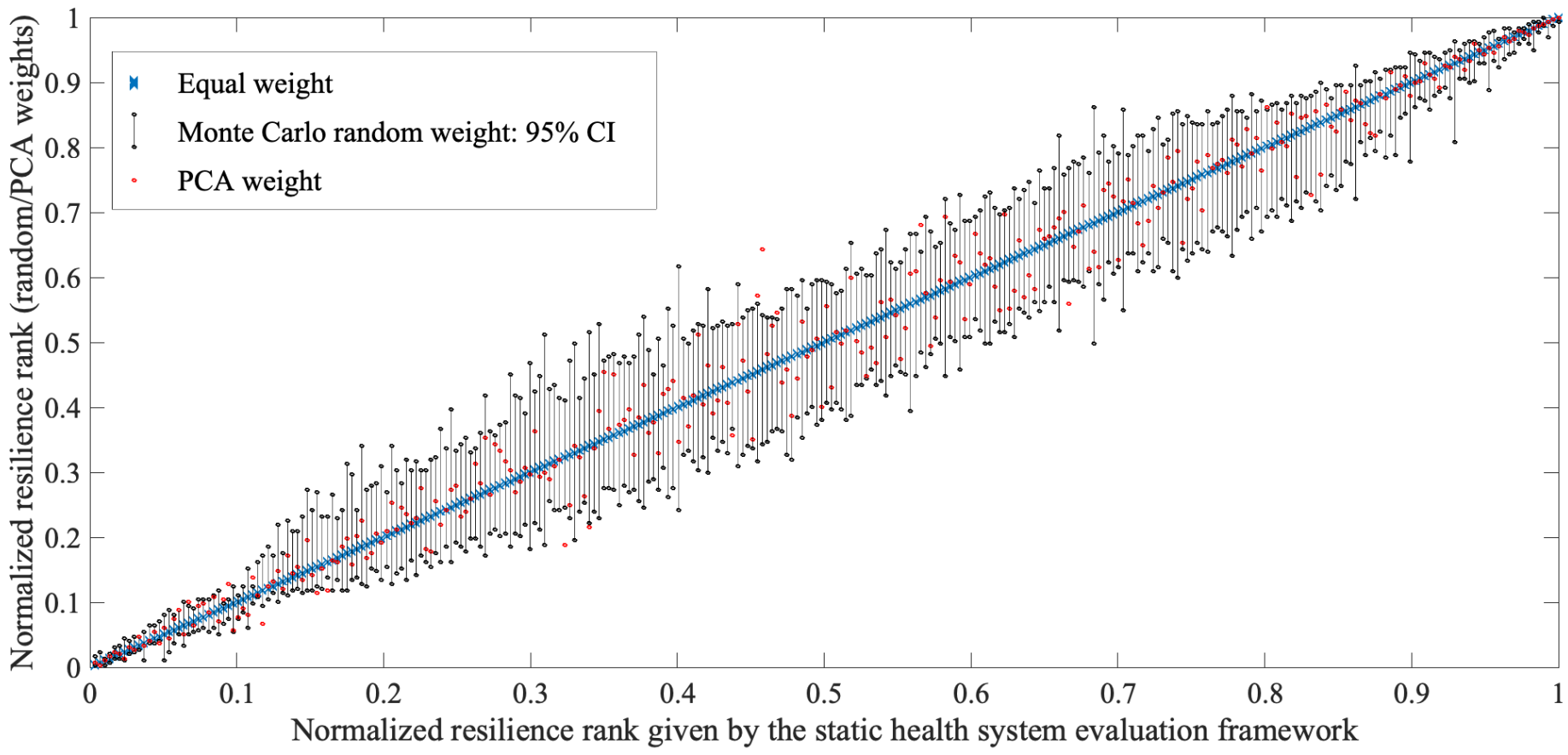

Fig. 16 | Sensitivity analysis of health system resilience assessment using Monte Carlo randomization method and PCA data-driven approach. The blue cross points represent the resilience rank given by equal-weighting approach, while the 95% confidence interval indicates the resilience rank given by alternative weighting schemes simulated by the Monte Carlo method. The red dots represent the normalized resilience rank given by PCA data-driven weights. The strong correlation indicated that the overall health system resilience was primarily determined by the variations of resilience indicators and pillars, rather than the specific weighting schemes. These sensitivity analysis demonstrated the robustness and reliability of our comprehensive health system evaluation framework under different weighting scenarios.

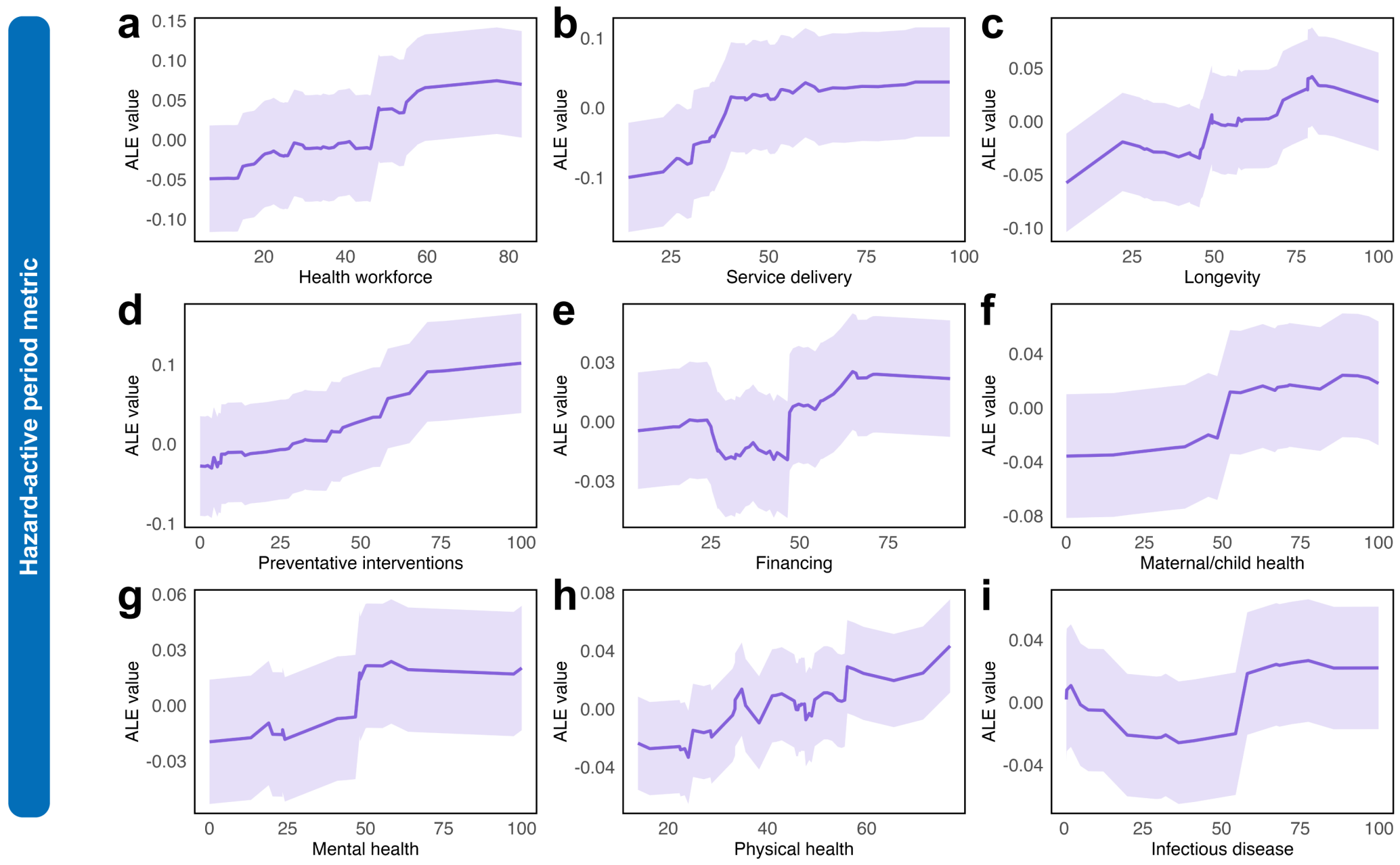

Fig. 17 | Sensitivity analysis using accumulated local effect (ALE) plots of health system resilience metrics on hazard-active period metric toward Climate hazards.

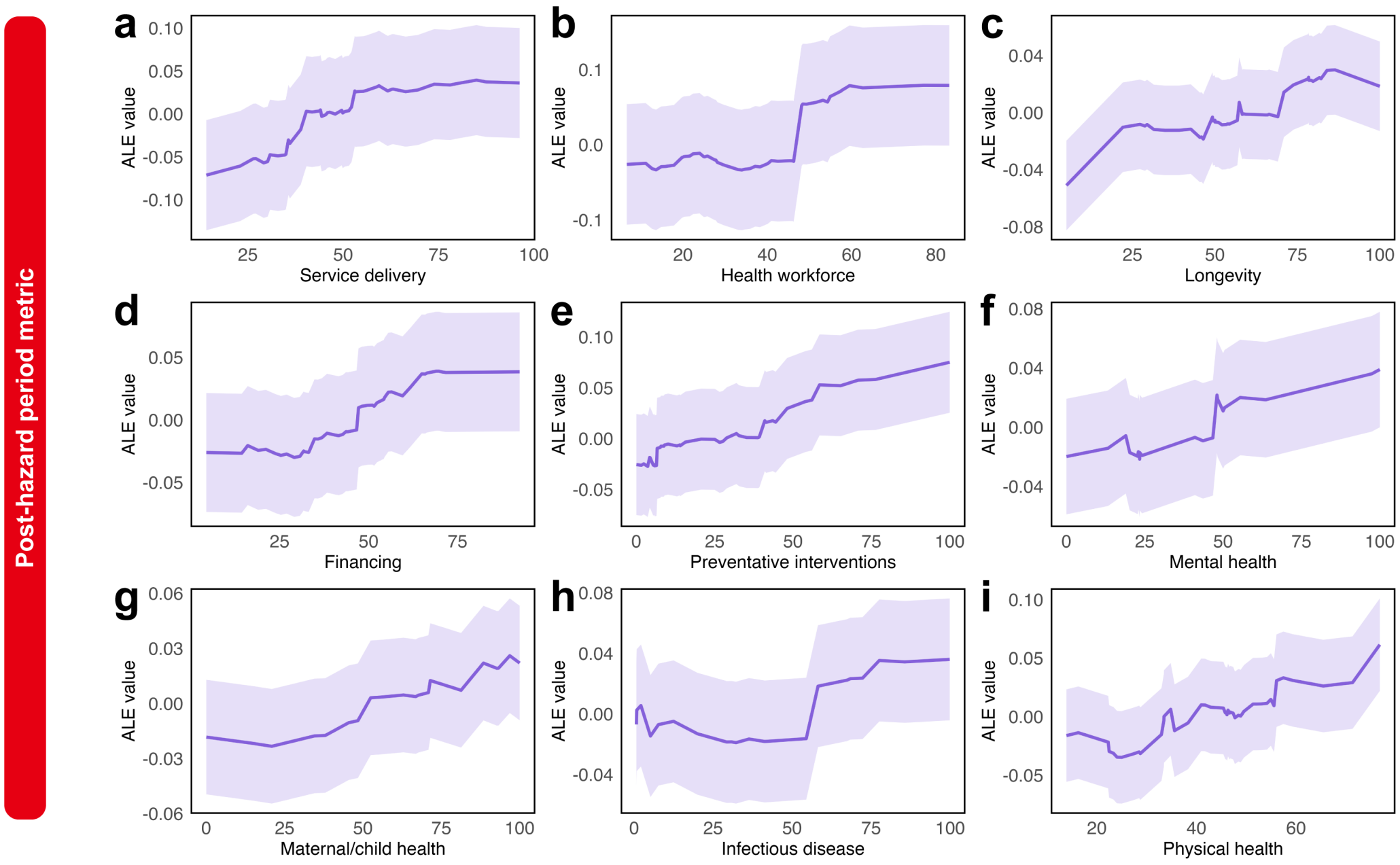

Fig. 18 | Sensitivity analysis using accumulated local effect (ALE) plots of health system resilience metrics on post-hazard period metric toward Climate hazards.

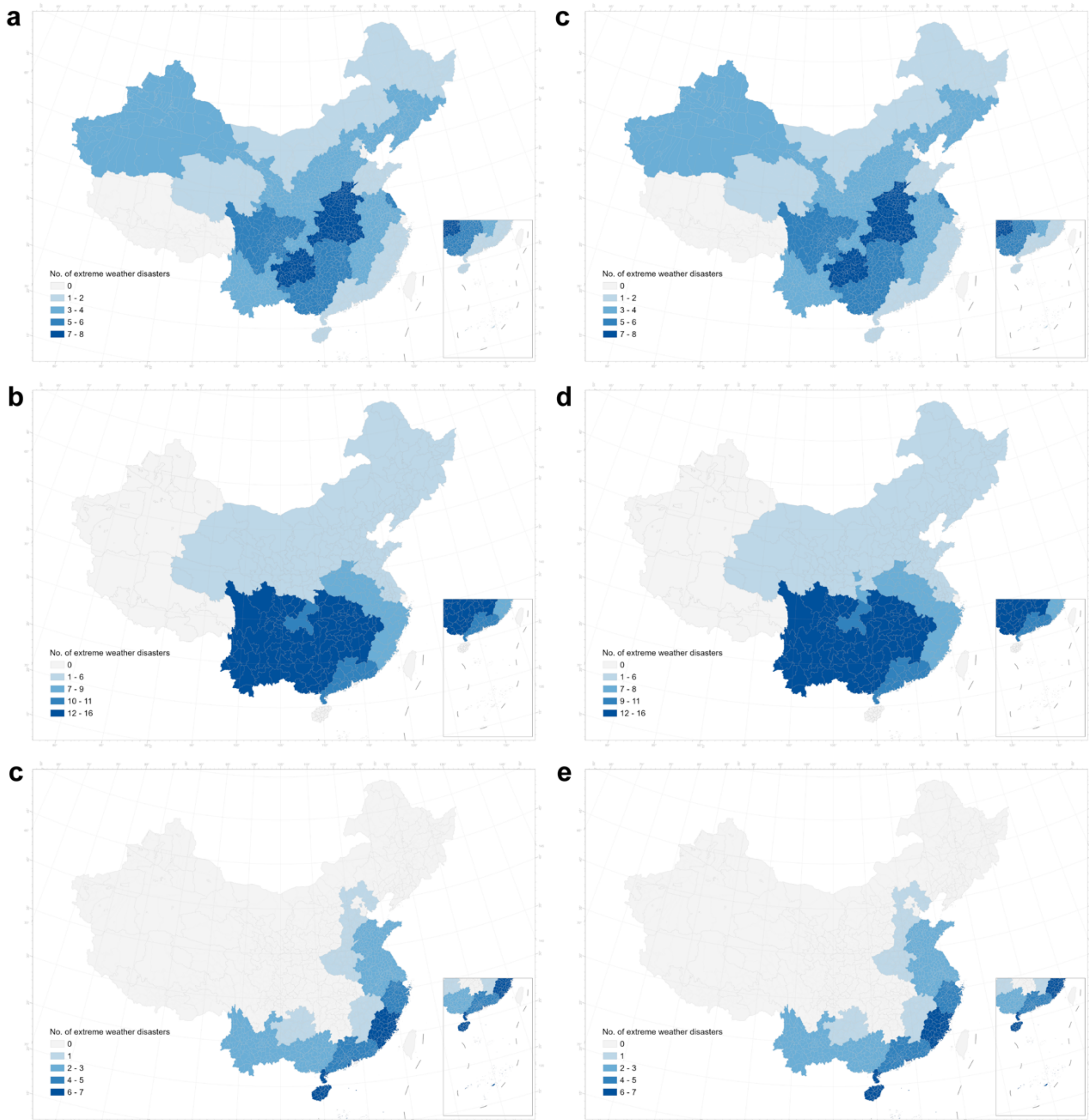

Fig. 19 | Hazard cross-validation between the EM-DAT and the GDIS databases. Data obtained from 1) the EM-DAT global disaster database[24]: **a,** storms **b,** floods **c,** tropical cyclones; and 2) the GDIS global disaster databases[28]: **d,** storms **e,** floods **f,** tropical cyclones. Note: we cross-validated hazard records with GDIS from 2016 to 2018, as GDIS records ended in 2018. The comparison of blizzard/winter storms were omitted as they were only recorded by the EM-DAT.

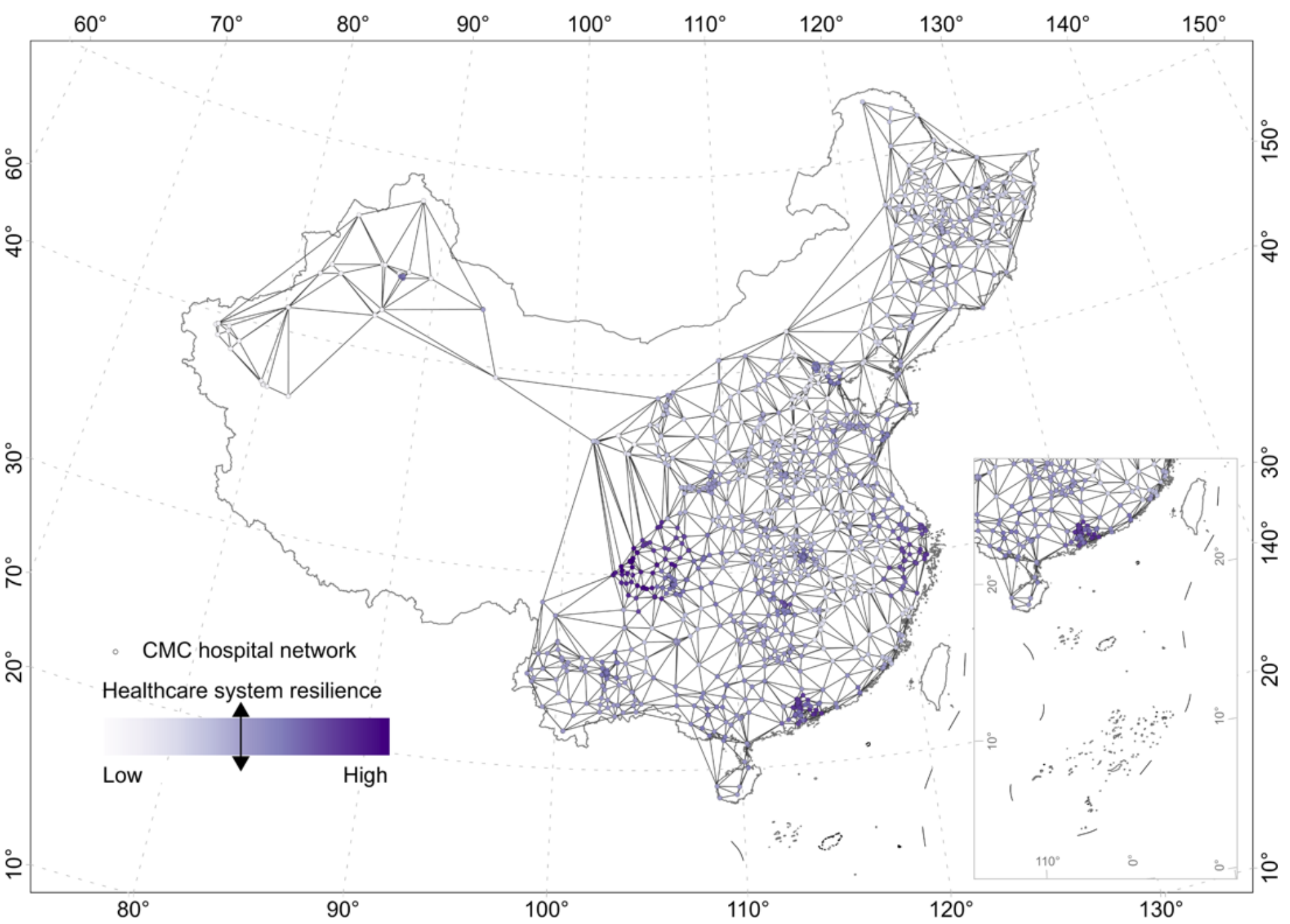

Fig. 20 | CMC Hospital Network. The colour indicates the local health system resilience marks.

Table 1 | Health system resilience metrics: pillars and indicators.

Pillar	Indicator	PC1	Database	References
Service delivery	Number of hospitals (per 1,000 persons)	0.215	NBS	15,52,53
	Number of inpatient beds	0.129	NHSA	1,54,55
	Average bed occupancy ratio	0.113	NHSA	1
	Average number of health workers per inpatient bed	0.232	NHSA	1,56,57
	Outpatient department, primary health care and emergency department service utilization rate	0.185	NHSA	1,58,59
	Average length of stay (ALOS) at hospital or health facility	0.005	NHSA	1
	Average number of consultations per capita per year	0.186	NHSA	1,59
Health workforce	Number of health workers per 10,000 population by occupation	0.159	NBS	1,60,61
	Number of community health workers per 1,000 population	0.279	NHSA	1,58,59
	Average number of staff, by occupation in primary care facilities and hospitals	0.165	NHSA	1,58,59
	Rural-to-urban distribution ratio of health workers	-0.208	NHSA	1,62,63
	Percentage of health workers that received training and qualifications from an accredited educational or training institution or body	-0.024	NHSA	1,64
	Percentage of births attended by skilled personnel (doctors, nurses, midwives)	0.095	NHSA	1,65
Preventative interventions	Consultations on health education technology and policy recommendations	0.038	NHSA	66,67
	Health education activities	0.037	NHSA	68
	Fluoride (water type) prevention and control	-0.061	NHSA	66-68
	Iodine deficiency disease prevention and treatment	-0.093	NHSA	66-68
Longevity	Life expectancy	0.220	NHSA	15,69
	Mortality rate (per 10,000 persons per year)	0.020	NHSA	15,69,70
Financing	Government financial allocation for healthcare	0.260	NHSA	52,71,72
	Healthcare expenditure per 10,000 persons	0.040	NHSA	1,73,74
	General government expenditure on primary health care (PHC) as a share of general government health expenditure	-0.088	NHSA	1,73,74
	Price index for medicine and health products	-0.121	NHSA	68
	Price index for medical service	-0.055	NHSA	68
	Healthcare insurance cover rate	0.300	NHSA	15,75
	Participation rate in pension insurance	0.305	NHSA	75,76
Physical health	ED visit rate (per 100 hospitals)	-0.162	NHSA	66,70
	Physician workload	0.212	NHSA	77

	Food safety and foodborne disease reports (per year)	-0.054	NHSA	78-81
	General practice outpatient and emergency visits rate	0.071	NHSA	66,70
Mental health	Mental disorder hospitalization incidence rate	0.060	NHSA	15,70
	Psychiatric outpatient and emergency visits rate	0.208	NHSA	70
Maternal/child health	Maternal healthcare system management rate (%)	0.187	NHSA	15,70
	Maternal healthcare antenatal examination rate (%)	0.136	NHSA	15,70
	Maternal healthcare postpartum visit rate (%)	0.154	NHSA	70
	Neonatal visit rate due to vaccination received (%)	0.140	NHSA	77
	Healthcare management rate for children under 7-year-old (%)	0.216	NHSA	82
Infectious disease	Number of beds designated for isolation of infectious diseases at the healthcare facility - incidence rate (per 100,000 persons per year)	-0.080	NHSA	15,70,77
	Number of beds designated for isolation of infectious diseases at the healthcare facility - mortality rate (per 100,000 persons per year)	-0.029	NHSA	15,70,77

Abbreviation: NBS, National Bureau of Statistics[13]; NHSA, National Healthcare Security Administration[33]; ED, emergency department.

Table 2 | Patient characteristics registered by the Chinese Multiple Counties Hospital Network in 2016-2023.

Characteristics	Number (%)
Total hospitalizations	41,747,482
Respiratory diseases	6,078,654 (14.56)
Injuries	3,033,260 (7.27)
Infectious diseases	1,399,566 (3.35)
Mental disorders	392,924 (0.94)
Neoplasms	3,168,945 (7.59)
Endocrine diseases	883,017 (2.12)
Neurological diseases	1,488,429 (3.57)
Genitourinary diseases	5,330,752 (12.77)
Cardiovascular diseases	6,245,250 (14.96)
Other diseases	13,726,685 (32.88)
Sex	
Male	19,634,042 (47.10)
Female	22,060,115 (52.90)
Age group (years)	
Less 18	6,068,391 (14.54)
18-44	9,721,168 (23.29)
45-64	13,592,431 (32.56)
Over 65	12,365,492 (29.61)
Sources before hospitalization	
Emergency department	30,352,545 (72.71)
Outpatient	9,506,697 (22.77)
Other healthcare institutions	1,888,240 (4.52)

Abbreviations: CVDs - cardiovascular diseases; ED - emergency department.

Table 3 | County-scale climate hazard exposure characteristics

Climate hazard	Directly affected	Indirectly affected (Spillover)		
	Disaster-exposed	N-I	N-II	N-III
Storm	521 (4.35)	81 (4.14)	388 (3.79)	662 (3.84)
Flood	1,959 (7.00)	258 (7.02)	957 (6.50)	1,716 (6.50)
Tropical cyclone	324 (2.30)	70 (2.63)	319 (2.61)	601 (2.45)
Blizzard/Winter storm	437 (4.85)	27 (5.22)	123 (5.41)	215 (5.22)

Note: the number before and within the brackets respectively represents the total number of county-counted climate hazards (2016-2023) and the mean duration days.

Table 4 | Relative changes of cause-specific hospitalization rates in N-I (0-100 km) regions.

Enduring period

Disease	Resilience	Storm	Flood	Tropical cyclone	Blizzard/Winter storm
Respiratory	Overall	5.5 [-3.26, 14.27]	6.32 [1.97, 10.66]	8.74 [-4.68, 22.15]	7.52 [-6.51, 21.56]
Injuries	Overall	2.32 [-6.73, 11.37]	4.07 [0.58, 7.56]	3.62 [-7.26, 14.5]	4.26 [-7.31, 15.82]
Infectious	Overall	5.27 [-0.76, 11.29]	4.49 [1.69, 7.29]	5.71 [-3.81, 15.24]	6.92 [-3.03, 16.86]
Mental	Overall	4.16 [0.04, 8.28]	2.44 [0.8, 4.07]	4.55 [-1.2, 10.29]	4.75 [-1.87, 11.37]
Neoplasms	Overall	2.07 [-5.16, 9.31]	3.99 [0.34, 7.64]	2.36 [-8.66, 13.38]	2.37 [-8.01, 12.75]
Endocrine	Overall	4.03 [-0.74, 8.81]	2.75 [0.02, 5.48]	5.31 [-3.17, 13.78]	4.71 [-3.19, 12.62]
Neurology	Overall	3.85 [-2.69, 10.38]	3.91 [1.1, 6.72]	4.32 [-4.38, 13.02]	2.23 [-5.9, 10.36]
Genitourinary	Overall	3.26 [-5.18, 11.7]	4.69 [0.51, 8.87]	3.88 [-9.28, 17.04]	4.92 [-6.54, 16.38]
CVD	Overall	6.07 [-3.09, 15.24]	7.13 [2.95, 11.3]	9 [-3.95, 21.95]	5.31 [-6.99, 17.61]

Stabilization-I period

Disease	Resilience	Storm	Flood	Tropical cyclone	Blizzard/Winter storm
Respiratory	Overall	5.26 [-4.45, 14.97]	5.84 [1.56, 10.13]	6.81 [-2.62, 16.24]	4.47 [-5.54, 14.49]
Injuries	Overall	1.93 [-5.98, 9.84]	3.51 [0.25, 6.77]	7.75 [-0.47, 15.97]	2.93 [-5.11, 10.97]
Infectious	Overall	4.89 [0.9, 8.89]	4.23 [1.94, 6.52]	6.51 [-0.17, 13.2]	4.97 [-0.63, 10.57]
Mental	Overall	3.65 [1.13, 6.17]	2.96 [1.71, 4.22]	3.46 [0.44, 6.48]	4.37 [0.79, 7.95]
Neoplasms	Overall	2.72 [-4.61, 10.04]	4.49 [1.07, 7.92]	1.51 [-8.19, 11.21]	1.64 [-5.53, 8.81]
Endocrine	Overall	3.55 [0.05, 7.05]	2.06 [0.16, 3.97]	4.27 [-0.95, 9.5]	4.23 [-2.15, 10.61]
Neurology	Overall	3.55 [-1.64, 8.74]	3.38 [0.91, 5.84]	4.05 [-1.68, 9.78]	2.37 [-2.7, 7.45]
Genitourinary	Overall	3.05 [-5.33, 11.44]	4.31 [0.24, 8.39]	4.44 [-7.14, 16.01]	4.14 [-6.29, 14.56]
CVD	Overall	4.84 [-4.75, 14.43]	4.22 [0.05, 8.38]	8.2 [-1.93, 18.33]	3.85 [-5.2, 12.9]

Stabilization-II period

Disease	Resilience	Storm	Flood	Tropical cyclone	Blizzard/Winter storm
Respiratory	Overall	3.93 [-4.67, 12.52]	2.82 [-1.45, 7.08]	1.66 [-8.37, 11.7]	1.55 [-8.29, 11.39]
Injuries	Overall	2.61 [-3.93, 9.15]	0.22 [-3.02, 3.46]	1.28 [-7.34, 9.9]	2.22 [-4.88, 9.32]
Infectious	Overall	3.46 [-1.39, 8.31]	3.32 [1.1, 5.54]	2.86 [-3.83, 9.54]	2.89 [-2.5, 8.28]
Mental	Overall	2.47 [0.17, 4.78]	2.3 [1.11, 3.5]	2.05 [-0.73, 4.83]	1.82 [-2.56, 6.19]
Neoplasms	Overall	2.11 [-4.52, 8.74]	2.16 [-1.31, 5.62	2.55 [-6.49, 11.59]	0.69 [-7.28, 8.65]
Endocrine	Overall	1.52 [-2.22, 5.25]	1.43 [-0.45, 3.32]	0.72 [-4.03, 5.47]	1.82 [-2.91, 6.54]
Neurology	Overall	2.16 [-2.27, 6.59]	2.42 [0, 4.83]	1.83 [-4.08, 7.75]	1.28 [-3.94, 6.51]
Genitourinary	Overall	3 [-4.47, 10.47]	3.23 [-0.92, 7.39]	1.72 [-9.9, 13.34]	2.56 [-6.57, 11.7]
CVD	Overall	0.71 [-7.85, 9.28]	1.76 [-2.52, 6.04]	1.94 [-8.37, 12.25]	3.28 [-5.3, 11.86]

Return to normal period

Disease	Resilience	Storm	Flood	Tropical cyclone	Blizzard/Winter storm
Respiratory	Overall	0.34 [-8.62, 9.3]	0.53 [-3.69, 4.75]	0.23 [-9.98, 10.44]	-0.34 [-10.46, 9.78]
Injuries	Overall	-1.11 [-8.93, 6.72]	0.14 [-3.15, 3.43]	0.13 [-8.38, 8.63]	-0.06 [-7.41, 7.29]
Infectious	Overall	1.24 [-2.74, 5.23]	1.11 [-1.74, 3.96]	0.27 [-6.34, 6.88]	-0.03 [-5.44, 5.39]
Mental	Overall	0.82 [-1.88, 3.52]	0.08 [-1.82, 1.97]	-0.98 [-5.38, 3.42]	0.53 [-2.9, 3.96]
Neoplasms	Overall	-0.6 [-7.02, 5.83]	-0.87 [-4.98, 3.24]	0.09 [-9.12, 9.31]	0.56 [-8.8, 9.91]
Endocrine	Overall	0.74 [-2, 3.48]	1.43 [-0.47, 3.33]	0.1 [-4.97, 5.18]	1.03 [-5.23, 7.29]
Neurology	Overall	0.91 [-2.96, 4.78]	-0.19 [-2.94, 2.56]	-0.4 [-6.05, 5.24]	0.57 [-4.97, 6.11]
Genitourinary	Overall	0.58 [-7.01, 8.18]	0.96 [-3.59, 5.51]	0.12 [-11.68, 11.93]	-1.06 [-9.85, 7.72]
CVD	Overall	1.72 [-6.9, 10.35]	0.88 [-3.43, 5.2]	0.23 [-10.48, 10.93]	-1.33 [-9.46, 6.79]

CVD - cardiovascular disease

Table 5 | Relative changes of cause-specific hospitalization rates in N-II (100-200 km) regions.

Enduring period

Disease	Resilience	Storm	Flood	Tropical cyclone	Blizzard/Winter storm
Respiratory	Overall	4.44 [-0.72, 9.59]	5.49 [2.52, 8.46]	7.8 [1.33, 14.28]	5.67 [-3.56, 14.9]
Injuries	Overall	1.96 [-2.35, 6.27]	3.56 [1.28, 5.85]	1.24 [-4.2, 6.68]	3.28 [-3.45, 10]
Infectious	Overall	4.45 [0.87, 8.03]	4.1 [2.31, 5.88]	5.07 [0.71, 9.42]	6.22 [1.73, 10.72]
Mental	Overall	3.33 [1, 5.67]	1.81 [0.88, 2.74]	3.97 [1.14, 6.81]	3.81 [0.12, 7.49]
Neoplasms	Overall	3.25 [-1.08, 7.57]	3.09 [0.72, 5.45]	1.5 [-3.97, 6.97]	1.07 [-5.53, 7.68]
Endocrine	Overall	3.72 [0.75, 6.68]	2.26 [0.93, 3.59]	4.04 [0.32, 7.75]	4.12 [0.06, 8.19]
Neurology	Overall	3.56 [0, 7.11]	2.97 [1.16, 4.79]	3.97 [-0.38, 8.33]	1.99 [-3.37, 7.35]
Genitourinary	Overall	1.79 [-3.01, 6.6]	4 [1.29, 6.72]	2.43 [-3.8, 8.66]	4.24 [-3.12, 11.6]
CVD	Overall	4.15 [-0.89, 9.18]	4.87 [2.05, 7.69]	6.73 [0.03, 13.43]	4.66 [-3.47, 12.79]

Stabilization-I period

Disease	Resilience	Storm	Flood	Tropical cyclone	Blizzard/Winter storm
Respiratory	Overall	2.92 [-3.72, 9.57]	4.11 [1.29, 6.93]	6.46 [0.71, 12.21]	3.72 [-3.42, 10.87]
Injuries	Overall	1.82 [-4.24, 7.88]	3.3 [1.14, 5.45]	2.09 [-2.78, 6.97]	2.77 [-2.67, 8.22]
Infectious	Overall	4.26 [0.5, 8.02]	3.72 [2.11, 5.34]	4.92 [1.3, 8.54]	3.85 [0.54, 7.17]
Mental	Overall	2.64 [0.36, 4.93]	2.5 [1.67, 3.33]	2.96 [1.27, 4.65]	2.89 [1.13, 4.65]
Neoplasms	Overall	1.47 [-4.11, 7.05]	2.82 [0.62, 5.03]	1.85 [-3.11, 6.8]	0.89 [-4.55, 6.33]
Endocrine	Overall	3.09 [0.05, 6.13]	1.97 [0.74, 3.19]	3.61 [0.68, 6.54]	3.31 [0.89, 5.73]
Neurology	Overall	3.2 [-0.9, 7.29]	2.38 [0.71, 4.05]	3.81 [0.1, 7.51]	1.8 [-1.77, 5.36]
Genitourinary	Overall	1.28 [-4.88, 7.43]	3.65 [1.05, 6.24]	1.87 [-3.79, 7.53]	3.26 [-3.14, 9.66]
CVD	Overall	2.06 [-4.39, 8.5]	3.57 [0.84, 6.29]	5.99 [0.02, 11.95]	3.19 [-3.32, 9.7]

Stabilization-II period

Disease	Resilience	Storm	Flood	Tropical cyclone	Blizzard/Winter storm
Respiratory	Overall	2.07 [-3.59, 7.73]	3.21 [0.16, 6.25]	0.61 [-6.09, 7.3]	-0.32 [-8.48, 7.85]
Injuries	Overall	2.39 [-2.97, 7.75]	0.66 [-1.65, 2.97]	0.57 [-5.41, 6.55]	0.44 [-5.62, 6.5]
Infectious	Overall	1.91 [-1.4, 5.23]	3.5 [1.77, 5.23]	1.29 [-3.33, 5.91]	2.35 [-1.12, 5.82]
Mental	Overall	1.82 [0.23, 3.41]	1.62 [0.78, 2.46]	0.73 [-1.37, 2.83]	1.94 [-0.53, 4.41]
Neoplasms	Overall	1.57 [-3.14, 6.28]	2.73 [0.33, 5.13]	1.71 [-4.49, 7.92]	0.99 [-5.03, 7.02]
Endocrine	Overall	0.93 [-1.69, 3.55]	1.24 [-0.05, 2.52]	0.48 [-4.13, 5.09]	1.38 [-1.14, 3.91]
Neurology	Overall	1.52 [-1.97, 5.02]	1.99 [0.25, 3.72]	1.6 [-2.65, 5.85]	1.36 [-2.55, 5.28]
Genitourinary	Overall	1.38 [-3.8, 6.55]	2.98 [0.12, 5.84]	1.37 [-6.22, 8.96]	1.79 [-5.35, 8.92]
CVD	Overall	2.6 [-2.79, 7.99]	2.19 [-0.77, 5.16]	0.94 [-6.34, 8.22]	-0.17 [-7.51, 7.16]

Return to normal period

Disease	Resilience	Storm	Flood	Tropical cyclone	Blizzard/Winter storm
Respiratory	Overall	0.81 [-4.55, 6.16]	0.1 [-2.81, 3.02]	-0.9 [-7.4, 5.59]	2.03 [-6.16, 10.21]
Injuries	Overall	1.37 [-3.86, 6.59]	0.47 [-1.75, 2.7]	0.34 [-5.39, 6.07]	1.08 [-5.04, 7.21]
Infectious	Overall	1.08 [-1.72, 3.88]	-1.13 [-3.04, 0.77]	0.31 [-3.43, 4.05]	0.99 [-3.53, 5.5]
Mental	Overall	0.54 [-0.78, 1.86]	-0.29 [-1.6, 1.02]	0.24 [-2.79, 3.27]	1.58 [-1.97, 5.13]
Neoplasms	Overall	1.35 [-3.06, 5.76]	-1.83 [-4.1, 0.44]	-0.57 [-6.25, 5.11]	-0.12 [-6.61, 6.36]
Endocrine	Overall	1.1 [-1.77, 3.96]	0.57 [-1.25, 2.39]	-0.26 [-3.99, 3.47]	1.22 [-3.65, 6.08]
Neurology	Overall	0.44 [-2.59, 3.47]	-0.03 [-1.73, 1.66]	1.29 [-2.67, 5.25]	2.03 [-1.8, 5.85]
Genitourinary	Overall	0.78 [-4.62, 6.18]	-1.36 [-4.04, 1.32]	0.73 [-5.73, 7.2]	0.41 [-7.12, 7.94]
CVD	Overall	1.07 [-4.11, 6.26]	0.62 [-2.21, 3.46]	0.49 [-6.32, 7.29]	0.8 [-6.46, 8.07]

CVD - cardiovascular disease

Table 6 | Relative changes of cause-specific hospitalization rates in N-III (200-300 km) regions.

Enduring period

Disease	Resilience	Storm	Flood	Tropical cyclone	Blizzard/Winter storm
Respiratory	Overall	1.23 [-3.2, 5.66]	1.53 [-0.71, 3.76]	2.69 [-2.54, 7.93]	1.5 [-5.35, 8.35]
Injuries	Overall	0.18 [-3.34, 3.7]	1.23 [-0.51, 2.96]	1.03 [-3.49, 5.56]	1.56 [-3.88, 7.01]
Infectious	Overall	1.37 [-1.46, 4.19]	1.21 [-0.31, 2.72]	2.16 [-1.29, 5.61]	2.59 [-1.32, 6.49]
Mental	Overall	1.45 [-0.25, 3.14]	1.42 [0.71, 2.13]	1.48 [-0.61, 3.57]	1.34 [-2.11, 4.78]
Neoplasms	Overall	0.49 [-3.06, 4.04]	1.11 [-0.65, 2.86]	1.34 [-3.3, 5.98]	1.33 [-4.31, 6.97]
Endocrine	Overall	1.49 [-0.69, 3.67]	1.21 [0.19, 2.23]	1.75 [-1.15, 4.65]	2.67 [-0.19, 5.54]
Neurology	Overall	0.93 [-1.88, 3.73]	1.03 [-0.28, 2.35]	2.22 [-1.3, 5.75]	0.87 [-3.05, 4.79]
Genitourinary	Overall	0.47 [-3.52, 4.45]	0.3 [-1.96, 2.56]	0.56 [-4.56, 5.67]	0.67 [-5.11, 6.46]
CVD	Overall	1.76 [-2.43, 5.94]	2.27 [0.12, 4.43]	4.02 [-1.28, 9.33]	2.38 [-4.09, 8.86]

Stabilization-I period

Disease	Resilience	Storm	Flood	Tropical cyclone	Blizzard/Winter storm
Respiratory	Overall	1.98 [-3.43, 7.39]	-0.86 [-3.07, 1.36]	2.09 [-2.63, 6.8]	1.48 [-4.46, 7.41]
Injuries	Overall	0.86 [-4.08, 5.8]	1.09 [-0.61, 2.79]	1.14 [-2.94, 5.21]	1.53 [-2.81, 5.87]
Infectious	Overall	0.72 [-2.98, 4.42]	1.4 [0.12, 2.69]	2.19 [-0.77, 5.16]	1.81 [-0.8, 4.41]
Mental	Overall	1.77 [-0.51, 4.06]	1.19 [0.53, 1.84]	1.18 [-0.42, 2.79]	1.47 [0.32, 2.61]
Neoplasms	Overall	0.14 [-4.55, 4.82]	-1.04 [-2.75, 0.67]	1.15 [-3, 5.31]	-0.56 [-4.82, 3.71]
Endocrine	Overall	1.7 [-0.82, 4.22]	0.62 [-0.58, 1.82]	0.27 [-2.84, 3.38]	1.46 [-0.21, 3.13]
Neurology	Overall	-0.38 [-6.32, 5.57]	0.52 [-0.76, 1.8]	1.68 [-1.37, 4.72]	0.84 [-1.95, 3.62]
Genitourinary	Overall	0.19 [-5.2, 5.59]	0.39 [-2.42, 1.63]	1.05 [-3.96, 6.07]	-0.32 [-5.39, 4.74]
CVD	Overall	0.63 [-4.58, 5.83]	0.46 [-1.8, 2.73]	1.85 [-3.01, 6.7]	1.73 [-3.56, 7.03]

Stabilization-II period

Disease	Resilience	Storm	Flood	Tropical cyclone	Blizzard/Winter storm
Respiratory	Overall	0.77 [-3.99, 5.54]	0.72 [-3.15, 1.71]	-1.07 [-6.85, 4.7]	1.67 [-5.2, 8.54]
Injuries	Overall	-1.25 [-5.83, 3.34]	-0.13 [-2.33, 2.06]	0.6 [-3.81, 5]	0.82 [-4.05, 5.69]
Infectious	Overall	0.7 [-2.26, 3.67]	-0.7 [-2.42, 1.02]	1.04 [-2.9, 4.98]	0.88 [-1.7, 3.46]
Mental	Overall	0.3 [-2.11, 2.7]	-0.44 [-1.53, 0.64]	-0.55 [-3.15, 2.05]	0.91 [-0.13, 1.95]
Neoplasms	Overall	-0.41 [-5.07, 4.24]	0.74 [-1.15, 2.64]	0 [-5.22, 5.22]	0.03 [-6.37, 6.44]
Endocrine	Overall	1.43 [-0.77, 3.62]	0.56 [-0.93, 2.04]	1.23 [-1.19, 3.65]	0.9 [-1.03, 2.83]
Neurology	Overall	0.86 [-2.17, 3.89]	0.62 [-1.22, 2.47]	1.81 [-1.49, 5.1]	0.83 [-2.15, 3.81]
Genitourinary	Overall	-0.74 [-5.09, 3.62]	-0.97 [-3.22, 1.28]	-0.46 [-6.26, 5.34]	-1.67 [-9.15, 5.81]
CVD	Overall	0.98 [-3.59, 5.55]	-0.82 [-3.16, 1.53]	0.93 [-4.38, 6.23]	-0.75 [-6.92, 5.41]

Return to normal period

Disease	Resilience	Storm	Flood	Tropical cyclone	Blizzard/Winter storm
Respiratory	Overall	1.18 [-3.48, 5.83]	-0.24 [-2.44, 1.97]	0.71 [-6.26, 4.83]	1.14 [-6.09, 8.37]
Injuries	Overall	0.47 [-3.21, 4.16]	0.6 [-1.41, 2.62]	0.46 [-5.13, 6.04]	0.15 [-6.53, 6.82]
Infectious	Overall	0.45 [-2.22, 3.13]	0.01 [-1.58, 1.6]	-1.1 [-5.4, 3.21]	-1.18 [-6.17, 3.8]
Mental	Overall	0.26 [-1.96, 2.47	0.71 [0.05, 1.36]	-0.37 [-3.2, 2.47]	1.31 [0.05, 2.56]
Neoplasms	Overall	0.99 [-2.8, 4.79]	-0.04 [-2.06, 1.99]	0 [-4.86, 4.86]	0.42 [-6.21, 7.04]
Endocrine	Overall	-0.55 [-3.55, 2.44]	-0.33 [-1.71, 1.05]	0.19 [-3.62, 3.99]	0.86 [-1.03, 2.75]
Neurology	Overall	0.61 [-2.23, 3.45]	0.93 [-0.76, 2.62]	-0.86 [-5.52, 3.79]	1.03 [-2.06, 4.11]
Genitourinary	Overall	0.09 [-4.14, 4.32]	0.88 [-2.38, 4.14]	-0.71 [-6.3, 4.89]	-0.26 [-6.33, 5.82]
CVD	Overall	1.05 [-3.36, 5.45]	0.61 [-1.55, 2.76]	0.64 [-6.36, 5.08]	0.33 [-6.3, 6.96]

CVD - cardiovascular disease

Table 7 | Sensitivity analysis of tertile-based resilience classifications.

Enduring period

Disease	Resilience	Storm	Flood	Tropical cyclone	Blizzard/Winter storm
Respiratory	High	7.58 [-3.24, 18.4]	8.92 [4.89, 12.95]	10.84 [2.15, 19.53]	11.02 [3.36, 18.68]
	Moderate	9.85 [1.03, 18.67]	12.54 [8.37, 16.71]	14.11 [1.53, 26.69]	16.27 [5.83, 26.71]
	Low	14.23 [4.69, 23.77]	18.36 [14.72, 22]	14.97 [-6.44, 36.38]	18.35 [10.43, 26.27]
Injuries	High	5.07 [-4.39, 14.53]	7.87 [4.28, 11.46]	9.18 [1.75, 16.61]	6.55 [1.29, 11.81]
	Moderate	8.73 [1.27, 16.19]	11.88 [8.69, 15.07]	12.83 [1.45, 24.21]	11.16 [1.84, 20.48]
	Low	9.34 [1.38, 17.3]	13.94 [11.12, 16.76]	16.27 [2.99, 29.55]	11.92 [7.84, 16]
Infectious	High	-7.03 [-13.48, -0.58]	-8.46 [-10.2, -6.72]	6.13 [0.89, 11.37]	7.63 [3.91, 11.35]
	Moderate	-8.92 [-15.62, -2.22]	-12.16 [-14.95, -9.37]	9.11 [0.53, 17.69]	12.06 [5.85, 18.27]
	Low	-10.21 [-14.29, -6.13]	-14.69 [-17.12, -12.26]	11.23 [1.73, 20.73]	14.31 [10.88, 17.74]
Mental	High	-5.33 [-9.36, -1.3]	-6.93 [-8.89, -4.97]	-5.33 [-9.02, -1.64]	-4.56 [-7.03, -2.09]
	Moderate	-7.85 [-11.2, -4.5]	-11.94 [-14.31, -9.57]	-7.92 [-14.88, -0.96]	-9.15 [-11.16, -7.14]
	Low	-11.84 [-15.43, -8.25]	-14.05 [-15.58, -12.52]	-8.23 [-16.45, -0.01]	-11.85 [-14.28, -9.42]
Neoplasms	High	-6.47 [-15.88, 2.94]	-6.33 [-8.75, -3.91]	-9.76 [-16.15, -3.37]	1.39 [-3.23, 6.01]
	Moderate	-11.72 [-18.73, -4.71]	-9.34 [-12.98, -5.7]	-15.93 [-26.94, -4.92]	2.78 [-6.42, 11.98]
	Low	-14.53 [-21.83, -7.23]	-10.74 [-13.82, -7.66]	-17.11 [-29.45, -4.77]	3.53 [-1.31, 8.37]
Endocrine	High	-6.58 [-11.73, -1.43]	-8.79 [-11.12, -6.46]	-8.12 [-12.81, -3.43]	-5.46 [-8.85, -2.07]
	Moderate	-9.36 [-14.62, -4.1]	-12.84 [-15.23, -10.45]	-12.67 [-20.44, -4.9]	-8.01 [-12.74, -3.28]
	Low	-10.74 [-14.92, -6.56]	-15.71 [-17.82, -13.6]	-12.83 [-23.34, -2.32]	-9.13 [-11.94, -6.32]
Neurology	High	-6.18 [-13.58, 1.22]	-7.89 [-10.57, -5.21]	-8.13 [-13.87, -2.39]	-8.27 [-12.43, -4.11]
	Moderate	-8.33 [-14.21, -2.45]	-10.93 [-13.89, -7.97]	-11.65 [-22.42, -0.88]	-10.75 [-15.86, -5.64]
	Low	-15.39 [-21.84, -8.94]	-12.45 [-14.26, -10.64]	-15.04 [-28.79, -1.29]	-13.69 [-16.84, -10.54]
Genitourinary	High	-4.64 [-15.12, 5.84]	-11.44 [-15.78, -7.1]	-13.02 [20.78, -46.82]	-8.12 [-15.68, -0.56]
	Moderate	-6.37 [-15.01, 2.27]	-14.97 [-18.94, -11]	-15.13 [-27.05, -3.21]	-11.24 [-20.42, -2.06]
	Low	-8.53 [-16.65, -0.41]	-17.35 [-20.66, -14.04]	-17.29 [-31.08, -3.5]	-14.13 [-19.03, -9.23]
CVD	High	7.24 [-2.13, 16.61]	5.76 [1.68, 9.84]	12.89 [5.42, 20.36]	5.03 [-1.32, 11.38]
	Moderate	10.44 [1.69, 19.19]	8.92 [5.13, 12.71]	15.03 [1.87, 28.19]	7.44 [-2.01, 16.89]
	Low	12.53 [2.48, 22.58]	13.57 [11.23, 15.91]	17.42 [2.44, 32.4]	9.58 [4.24, 14.92]

Stabilization-I period

Disease	Resilience	Storm	Flood	Tropical cyclone	Blizzard/Winter storm
Respiratory	High	4.56 [-4.32, 13.44]	6.68 [2.97, 10.39]	6.42 [1.07, 11.77]	8.21 [0.73, 15.69]
	Moderate	7.05 [-2.52, 16.62]	9.32 [5.44, 13.2]	9.13 [-3.26, 21.52]	13.82 [2.29, 25.35]
	Low	11.48 [1.84, 21.12]	12.59 [9.37, 15.81]	11.44 [-3.39, 26.27]	14.76 [9.07, 20.45]
Injuries	High	2.65 [-5.67, 10.97]	4.77 [1.21, 8.33]	4.59 [-2.16, 11.34]	2.87 [0.15, 5.59]
	Moderate	5.94 [-1.12, 13]	8.18 [5.23, 11.13]	6.98 [-4.86, 18.82]	4.94 [-3.85, 13.73]
	Low	7.35 [-0.79, 15.49]	9.93 [6.28, 13.58]	8.03 [-5.93, 21.99]	6.67 [1.52, 11.82]
Infectious	High	9.05 [1.73, 16.37]	9.21 [7.24, 11.18]	7.22 [2.32, 12.12]	6.99 [4.78, 9.2]
	Moderate	14.51 [7.68, 21.34]	16.03 [12.96, 19.1]	10.69 [1.83, 19.55]	9.47 [3.03, 15.91]

	Low	16.74 [10.09, 23.39]	17.89 [16.42, 19.36]	13.49 [3.74, 23.24]	10.62 [6.64, 14.6]
Mental	High	6.61 [2.56, 10.66]	7.85 [6.04, 9.66]	4.37 [1.29, 7.45]	5.64 [2.76, 8.52]
	Moderate	11.04 [7.87, 14.21]	10.33 [8.72, 11.94]	7.32 [1.38, 13.26]	11.07 [6.48, 15.66]
	Low	12.36 [7.95, 16.77]	14.68 [13.79, 15.57]	8.07 [1.45, 14.69]	12.93 [9.42, 16.44]
Neoplasms	High	4.13 [-3.95, 12.21]	5.14 [1.86, 8.42]	7.76 [1.27, 14.25]	0.47 [-6.93, 7.87]
	Moderate	9.94 [1.36, 18.52]	6.77 [3.42, 10.12]	13.14 [1.78, 24.5]	1.32 [-7.35, 9.99]
	Low	11.93 [3.78, 20.08]	9.13 [6.82, 11.44]	14.29 [1.63, 26.95]	1.54 [-3.19, 6.27]
Endocrine	High	4.62 [0.41, 8.83]	8.74 [5.89, 11.59]	5.79 [1.75, 9.83]	3.78 [0.36, 7.2]
	Moderate	9.11 [3.78, 14.44]	11.05 [8.74, 13.36]	8.93 [1.92, 15.94]	6.27 [1.84, 10.7]
	Low	12.63 [7.94, 17.32]	15.16 [12.85, 17.47]	10.88 [0.04, 21.72]	6.92 [3.68, 10.16]
Neurology	High	4.75 [-1.97, 11.47]	4.03 [1.55, 6.51]	7.09 [1.84, 12.34]	4.54 [0.31, 8.77]
	Moderate	8.05 [2.33, 13.77]	6.98 [4.16, 9.8]	11.86 [2.23, 21.49]	6.29 [0.62, 11.96]
	Low	12.01 [4.72, 19.3]	8.21 [5.74, 10.68]	13.72 [0.41, 27.03]	7.85 [3.71, 11.99]
Genitourinary	High	2.89 [-5.33, 11.11]	9.14 [5.86, 12.42]	10.03 [1.92, 18.14]	7.23 [0.68, 13.78]
	Moderate	6.71 [-2.46, 15.88]	14.04 [10.83, 17.25]	13.14 [0.06, 26.22]	10.07 [0.35, 19.79]
	Low	8.13 [0.32, 15.94]	15.98 [12.84, 19.12]	14.72 [0.14, 29.3]	15.83 [10.32, 21.34]
CVD	High	5.26 [-3.89, 14.41]	3.88 [0.46, 7.3]	7.23 [-0.27, 14.73]	4.89 [-2.48, 12.26]
	Moderate	8.14 [-0.24, 16.52]	6.03 [2.04, 10.02]	10.76 [-2.02, 23.54]	7.47 [-3.82, 18.76]
	Low	10.87 [1.38, 20.36]	8.84 [5.12, 12.56]	11.79 [-3.63, 27.21]	8.25 [2.94, 13.56]

Stabilization-II period

Disease	Resilience	Storm	Flood	Tropical cyclone	Blizzard/Winter storm
Respiratory	High	2.37 [-7.35, 12.09]	2.78 [-0.42, 5.98]	5.15 [-2.21, 12.51]	4.11 [-3.23, 11.45]
	Moderate	2.83 [-6.93, 12.59]	4.33 [0.39, 8.27]	6.67 [-5.83, 19.17]	5.36 [-6.21, 16.93]
	Low	3.74 [-3.69, 11.17]	5.46 [2.05, 8.87]	8.24 [-7.98, 24.46]	5.78 [0.42, 11.14]
Injuries	High	1.07 [-7.12, 9.26]	1.28 [-1.04, 3.6]	1.89 [-3.86, 7.64]	1.74 [-4.23, 7.71]
	Moderate	1.64 [-5.82, 9.1]	2.36 [-0.24, 4.96]	4.79 [-5.97, 15.55]	2.67 [-6.14, 11.48]
	Low	2.49 [-4.91, 9.89]	2.53 [-0.07, 5.13]	4.89 [-8.52, 18.3]	3.98 [-1.16, 9.12]
Infectious	High	5.62 [-1.74, 12.98]	3.32 [1.37, 5.27]	5.32 [0.36, 10.28]	3.56 [-0.48, 7.6]
	Moderate	5.97 [-0.32, 12.26]	4.57 [2.36, 6.78]	6.45 [-1.98, 14.88]	5.13 [-0.73, 10.99]
	Low	7.73 [2.63, 12.83]	5.89 [4.21, 7.57]	5.89 [-3.21, 14.99]	5.46 [2.23, 8.69]
Mental	High	3.78 [-0.24, 7.8]	1.78 [0.47, 3.09]	2.67 [-0.44, 5.78]	2.72 [0.06, 5.38]
	Moderate	6.02 [2.74, 9.3]	2.83 [1.76, 3.9]	3.79 [-2.33, 9.91]	4.13 [0.76, 7.5]
	Low	6.94 [2.42, 11.46]	3.23 [2.07, 4.39]	4.43 [-2.63, 11.49]	4.29 [2.21, 6.37]
Neoplasms	High	3.67 [-5.42, 12.76]	2.47 [-0.32, 5.26]	4.58 [-1.42, 10.58]	0.47 [-5.22, 6.16]
	Moderate	4.49 [-3.74, 12.72]	3.57 [0.26, 6.88]	6.72 [-4.58, 18.02]	0.84 [-7.93, 9.61]
	Low	6.21 [-1.69, 14.11]	4.49 [1.98, 7]	6.61 [-6.57, 19.79]	1.35 [-2.87, 5.57]
Endocrine	High	2.48 [-2.41, 7.37]	3.12 [1.03, 5.21]	4.79 [0.47, 9.11]	3.3 [-0.11, 6.71]
	Moderate	3.38 [-1.42, 8.18]	4.08 [1.86, 6.3]	6.42 [-0.47, 13.31]	4.32 [-0.27, 8.91]
	Low	4.62 [-0.33, 9.57]	4.77 [3.51, 6.03]	8.13 [-4.02, 20.28]	4.53 [1.78, 7.28]
	High	2.02 [-4.28, 8.32]	2.64 [0.37, 4.91]	3.46 [-0.42, 7.34]	3.21 [-0.17, 6.59]

Neurology	Moderate	3.31 [-3.03, 9.65]	3.32 [0.78, 5.86]	5.64 [-3.55, 14.83]	4.42 [-1.58, 10.42]
	Low	3.87 [-2.94, 10.68]	3.61 [1.62, 5.6]	7.99 [-5.04, 21.02]	5.37 [1.29, 9.45]
Genitourinary	High	1.79 [-6.37, 9.95]	3.97 [0.41, 7.53]	3.82 [-3.74, 11.38]	2.66 [-4.83, 10.15]
	Moderate	3.42 [-4.59, 11.43]	4.78 [1.33, 8.23]	4.92 [-6.84, 16.68]	4.29 [-6.05, 14.63]
	Low	4.02 [-3.64, 11.68]	5.64 [2.58, 8.7]	8.01 [-5.83, 21.85]	5.56 [0.52, 10.6]
CVD	High	3.03 [-6.02, 12.08]	2.72 [-0.53, 5.97]	4.22 [-2.79, 11.23]	3.46 [-3.59, 10.51]
	Moderate	4.95 [-5.36, 15.26]	4.13 [0.62, 7.64]	5.33 [-6.89, 17.55]	4.87 [-5.36, 15.1]
	Low	5.38 [-4.21, 14.97]	4.67 [1.38, 7.96]	7.93 [-6.41, 22.27]	5.13 [-0.92, 11.18]

Return to normal period

Disease	Resilience	Storm	Flood	Tropical cyclone	Blizzard/Winter storm
Respiratory	High	0.53 [-9.32, 10.38]	1.03 [-0.24, 2.3]	1.13 [-5.84, 8.1]	1.83 [-2.01, 5.67]
	Moderate	0.78 [-8.54, 10.1]	1.78 [-3.12, 6.68]	1.89 [-10.92, 14.7]	2.37 [-8.33, 13.07]
	Low	1.35 [-7.91, 10.61]	2.02 [-1.49, 5.53]	2.21 [-13.52, 17.94]	2.41 [-2.52, 7.34]
Injuries	High	0.31 [-7.98, 8.6]	-0.38 [-4.57, 3.81]	0.38 [-5.73, 6.49]	0.83 [-5.21, 6.87]
	Moderate	0.78 [-6.04, 7.6]	0.23 [-3.61, 4.07]	1.12 [-10.87, 13.11]	1.58 [-6.93, 10.09]
	Low	1.29 [-7.53, 10.11]	0.58 [-1.92, 3.08]	1.46 [-12.42, 15.34]	2.22 [-2.45, 6.89]
Infectious	High	0.64 [-5.93, 7.21]	0.48 [-4.62, 5.58]	0.42 [-4.08, 4.92]	1.85 [-4.23, 7.93]
	Moderate	1.29 [-3.78, 6.36]	1.02 [-7.84, 9.88]	0.97 [-7.23, 9.17]	2.72 [-2.87, 8.31]
	Low	1.43 [-4.11, 6.97]	1.76 [-5.51, 9.03]	1.69 [-5.52, 8.9]	3.24 [-0.47, 6.95]
Mental	High	0.52 [-3.01, 4.05]	-0.32 [-2.59, 1.95]	-0.33 [-2.84, 2.18]	0.68 [-1.35, 2.71]
	Moderate	1.36 [-2.39, 5.11]	0.57 [-5.84, 6.98]	0.62 [-5.18, 6.42]	1.22 [-1.56, 4]
	Low	1.87 [-2.14, 5.88]	1.02 [-6.43, 8.47]	0.94 [-6.02, 7.9]	1.59 [-2.97, 6.15]
Neoplasms	High	0.15 [-8.98, 9.28]	0.46 [-4.89, 5.81]	0.54 [-5.13, 6.21]	0.21 [-6.55, 6.97]
	Moderate	0.93 [-6.82, 8.68]	1.83 [-9.48, 13.14]	1.78 [-10.08, 13.64]	0.43 [-8.92, 9.78]
	Low	1.01 [-7.21, 9.23]	2.11 [-9.83, 14.05]	1.93 [-9.27, 13.13]	0.67 [-3.42, 4.76]
Endocrine	High	0.42 [-4.88, 5.72]	0.61 [-2.47, 3.69]	0.82 [-2.53, 4.17]	1.55 [-1.48, 4.58]
	Moderate	0.86 [-4.02, 5.74]	1.73 [-5.62, 9.08]	1.43 [-5.42, 8.28]	1.78 [-2.17, 5.73]
	Low	1.13 [-3.94, 6.2]	1.58 [-7.83, 10.99]	1.67 [-7.96, 11.3]	2.29 [-0.32, 4.9]
Neurology	High	0.24 [-7.17, 7.65]	1.37 [-0.52, 3.26]	0.23 [-4.24, 4.7]	0.52 [-5.49, 6.53]
	Moderate	0.66 [-5.38, 6.7]	2.21 [-0.87, 5.29]	1.12 [-8.89, 11.13]	0.97 [-4.98, 6.92]
	Low	1.32 [-6.02, 8.66]	2.32 [0.33, 4.31]	1.78 [-8.32, 11.88]	1.78 [-2.22, 5.78]
Genitourinary	High	0.33 [-9.23, 9.89]	0.06 [-4.08, 4.2]	0.66 [-6.84, 8.16]	1.68 [-5.78, 9.14]
	Moderate	0.92 [-6.63, 8.47]	0.24 [-2.87, 3.35]	1.14 [-11.17, 13.45]	1.93 [-7.44, 11.3]
	Low	1.41 [-8.21, 11.03]	0.93 [-2.25, 4.11]	1.63 [-12.03, 15.29]	2.41 [-2.67, 7.49]
CVD	High	1.01 [-7.52, 9.54]	1.44 [-1.06, 3.94]	0.78 [-6.54, 8.1]	0.58 [-6.23, 7.39]
	Moderate	1.89 [-5.29, 9.07]	1.56 [-2.13, 5.25]	1.32 [-9.26, 11.9]	2.39 [-7.04, 11.82]
	Low	2.24 [-8.53, 13.01]	2.32 [-0.74, 5.38]	1.54 [-11.18, 14.26]	2.55 [-2.49, 7.59]

CVD - cardiovascular disease

Table 8 | Sensitivity analysis using county-level random effects.

Enduring period

Disease	Resilience	Storm	Flood	Tropical cyclone	Blizzard/Winter storm
Respiratory	Overall	8.73 [3.36, 14.1]	11.92 [9.58, 14.27]	10.98 [3.89, 18.06]	16.29 [12.08, 20.5]
	High	6.97 [-0.31, 14.25]	10.09 [7.15, 13.03]	9.95 [2.32, 17.58]	14.47 [8.26, 20.67]
	Low	10.5 [3.22, 17.78]	14.38 [10.98, 17.78]	17.23 [2.55, 31.9]	18.24 [13.18, 23.3]
Injuries	Overall	8.6 [4.44, 12.77]	9.26 [7.47, 11.04]	10.29 [3.99, 16.59]	10.82 [7.39, 14.26]
	High	8.11 [2.14, 14.07]	8.41 [6.16, 10.66]	10.14 [3.3, 16.98]	9.96 [4.75, 15.16]
	Low	9.1 [3.46, 14.73]	10.4 [7.78, 13.02]	12.13 [-1.26, 25.52]	11.31 [7.07, 15.55]
Infectious	Overall	-6.18 [-9.12, -3.23]	-10.53 [-11.88, -9.18]	6.9 [1.81, 11.98]	12.49 [9.75, 15.23]
	High	-5.49 [-9.73, -1.25]	-9.43 [-11.12, -7.74]	6.24 [0.82, 11.65]	9.83 [5.71, 13.95]
	Low	-6.85 [-10.84, -2.86]	-12.3 [-14.35, -10.24]	10.94 [-2.16, 24.03]	15.22 [11.71, 18.73]
Mental	Overall	-6.26 [-8.44, -4.08]	-9.58 [-10.47, -8.68]	-6.24 [-9.5, -2.97]	-8.16 [-9.93, -6.38]
	High	-4.46 [-7.34, -1.59]	-8.3 [-9.41, -7.2]	-5.96 [-9.42, -2.5]	-5.96 [-8.72, -3.2]
	Low	-8.05 [-11.26, -4.83]	-11.55 [-13.04, -10.07]	-7.92 [-17.21, 1.37]	-10.53 [-12.84, -8.22]
Neoplasms	Overall	-7.39 [-11.6, -3.19]	-7.5 [-9.31, -5.69]	-10.67 [-17.62, -3.71]	4.64 [1.16, 8.13]
	High	-6.39 [-12.3, -0.49]	-6.53 [-8.84, -4.23]	-9.72 [-17.25, -2.2]	3.62 [-1.72, 8.97]
	Low	-8.37 [-14.05, -2.7]	-9.25 [-11.87, -6.63]	-14.27 [-28.28, -0.25]	5.14 [0.79, 9.48]
Endocrine	Overall	-6.53 [-9.03, -4.03]	-10.7 [-11.8, -9.6]	-9.87 [-14.1, -5.64]	-6.46 [-8.63, -4.28]
	High	-5.73 [-9.4, -2.07]	-9.78 [-11.14, -8.41]	-9.56 [-14.11, -5.01]	-4.82 [-7.87, -1.76]
	Low	-7.36 [-10.75, -3.97]	-12.17 [-13.96, -10.39]	-11.74 [-22.13, -1.35]	-8.59 [-11.64, -5.54]
Neurology	Overall	-7.69 [-10.92, -4.45]	-9.5 [-10.88, -8.13]	-8.7 [-13.54, -3.87]	-8.82 [-11.31, -6.33]
	High	-7.16 [-11.97, -2.35]	-9.02 [-10.73, -7.3]	-7.57 [-12.59, -2.55]	-7.87 [-11.61, -4.12]
	Low	-8.2 [-12.48, -3.93]	-10.36 [-12.48, -8.24]	-15.58 [-31.29, 0.12]	-10.4 [-13.56, -7.23]
Genitourinary	Overall	-5.38 [-9.95, -0.8]	-13.09 [-15.27, -10.92]	-12.01 [-19.51, -4.52]	-10.48 [-14.45, -6.52]
	High	-4.16 [-10.59, 2.26]	-12.43 [-15.15, -9.72]	-11.29 [-19.43, -3.16]	-9.33 [-15.66, -3]
	Low	-6.55 [-12.64, -0.46]	-14.42 [-17.55, -11.29]	-16.45 [-30.93, -1.98]	-12.58 [-17.11, -8.05]
CVD	Overall	9.6 [4.45, 14.75]	9.74 [7.49, 11.98]	12.46 [5.24, 19.68]	9.23 [5.18, 13.28]
	High	7.75 [0.59, 14.91]	8.8 [6.02, 11.57]	11.95 [4.1, 19.79]	8.22 [2.16, 14.27]
	Low	11.46 [4.53, 18.38]	10.67 [7.42, 13.92]	15.64 [1.67, 29.61]	10.32 [5.5, 15.14]

Stabilization-I period

Disease	Resilience	Storm	Flood	Tropical cyclone	Blizzard/Winter storm
Respiratory	Overall	2.88 [0.06, 5.71]	8.65 [7.01, 10.29]	4.62 [1.66, 7.57]	9.77 [7.13, 12.4]
	High	2.38 [-1.44, 6.2]	7.44 [5.26, 9.63]	4.98 [1.6, 8.37]	12.06 [8.26, 15.86]
	Low	3.31 [-0.35, 6.97]	9.92 [7.75, 12.1]	3.1 [-1.34, 7.54]	8.56 [5.42, 11.7]
Injuries	Overall	2.8 [0.58, 5.02]	5.09 [3.82, 6.35]	4.02 [1.46, 6.58]	4.08 [2.03, 6.13]
	High	2.73 [-0.42, 5.87]	4.16 [2.48, 5.84]	3.63 [0.7, 6.56]	5.28 [2.22, 8.34]
	Low	2.86 [-0.06, 5.77]	6.25 [4.52, 7.98]	5.55 [1.31, 9.8]	3.19 [0.68, 5.69]
Infectious	Overall	5.54 [3.84, 7.25]	8.25 [7.28, 9.21]	5.61 [3.56, 7.66]	5.61 [4.02, 7.2]
	High	4.3 [1.92, 6.67]	7.59 [6.32, 8.86]	5.32 [3, 7.64]	6.57 [4.09, 9.05]

	Low	6.66 [4.32, 9.01]	8.97 [7.61, 10.33]	6.73 [2.8, 10.66]	6.35 [3.09, 9.6]
Mental	Overall	5.78 [4.55, 7.01]	6.5 [5.89, 7.12]	3.98 [2.69, 5.26]	5.58 [4.64, 6.52]
	High	4.95 [3.19, 6.72]	5.69 [4.9, 6.49]	3.82 [2.39, 5.24]	5.13 [3.7, 6.56]
	Low	6.52 [4.81, 8.23]	7.64 [6.71, 8.58]	4.6 [1.72, 7.47]	6.02 [4.8, 7.25]
Neoplasms	Overall	2.8 [0.2, 5.39]	5.09 [3.82, 6.36]	2.29 [-0.25, 4.83]	1.72 [-0.35, 3.79]
	High	1.93 [-1.75, 5.61]	4.46 [2.73, 6.2]	2.62 [-0.27, 5.51]	3.02 [-0.12, 6.16]
	Low	3.61 [0.24, 6.99]	5.77 [4.09, 7.45]	0.96 [-3.38, 5.3]	0.73 [-1.81, 3.28]
Endocrine	Overall	3.81 [2.4, 5.23]	5.44 [4.7, 6.18]	4.49 [2.88, 6.11]	4.47 [3.29, 5.65]
	High	2.59 [0.54, 4.65]	5.06 [4.08, 6.04]	4.27 [2.48, 6.07]	4.24 [2.5, 5.97]
	Low	4.91 [3.02, 6.8]	5.93 [4.85, 7.01]	5.35 [1.85, 8.84]	4.57 [3.01, 6.13]
Neurology	Overall	3.69 [1.64, 5.75]	5.76 [4.8, 6.72]	4.91 [2.87, 6.96]	4.02 [2.56, 5.47]
	High	1.16 [-1.33, 3.65]	5.3 [4.02, 6.58]	5.05 [2.75, 7.35]	3.83 [1.68, 5.98]
	Low	5.66 [2.66, 8.66]	6.14 [4.8, 7.49]	4.57 [0.46, 8.67]	4.43 [2.56, 6.3]
Genitourinary	Overall	0.83 [-1.61, 3.28]	8 [6.46, 9.55]	2.51 [-0.64, 5.66]	6.74 [4.32, 9.17]
	High	1.24 [-2.14, 4.62]	7.28 [5.21, 9.34]	2.46 [-1.17, 6.09]	7.86 [4.24, 11.49]
	Low	0.49 [-2.69, 3.67]	8.75 [6.73, 10.78]	2.62 [-1.87, 7.1]	6.29 [3.45, 9.13]
CVD	Overall	4.25 [1.26, 7.23]	5.85 [4.26, 7.44]	5.33 [2.33, 8.33]	3.94 [1.49, 6.38]
	High	3.46 [-0.72, 7.63]	5.21 [3.13, 7.29]	6.38 [2.93, 9.84]	6.89 [3.32, 10.45]
	Low	5.04 [1.21, 8.87]	6.13 [3.99, 8.27]	1.04 [-3.11, 5.19]	2.27 [-0.6, 5.14]

Stabilization-II period

Disease	Resilience	Storm	Flood	Tropical cyclone	Blizzard/Winter storm
Respiratory	Overall	2.21 [-0.55, 4.97]	6.39 [4.67, 8.1]	0.56 [-2.28, 3.4]	-0.83 [-3.5, 1.84]
	High	4.1 [0.34, 7.86]	6.6 [4.23, 8.97]	-0.8 [-4.03, 2.43]	0.23 [-3.71, 4.17]
	Low	0.47 [-3.11, 4.05]	3.63 [1.53, 5.73]	5.72 [1.1, 10.35]	-0.49 [-3.64, 2.66]
Injuries	Overall	0.44 [-1.74, 2.63]	1.98 [0.55, 3.41]	0.64 [-1.83, 3.12]	-0.2 [-2.24, 1.84]
	High	4.12 [1.01, 7.24]	1.67 [-0.24, 3.58]	0.67 [-2.16, 3.49]	-0.48 [-3.6, 2.64]
	Low	-2.9 [-5.76, -0.03]	0.63 [-1.32, 2.57]	0.56 [-3.52, 4.63]	0.79 [-1.65, 3.23]
Infectious	Overall	0.44 [-1.74, 2.63]	1.52 [0.66, 2.39]	1.62 [-0.66, 3.89]	2.76 [1.3, 4.21]
	High	4.12 [1.01, 7.24]	1.26 [0.12, 2.41]	1.23 [-1.4, 3.87]	2.32 [0.04, 4.6]
	Low	-2.9 [-5.76, -0.03]	1.47 [0.28, 2.66]	3.08 [-0.34, 6.5]	3.3 [1.49, 5.1]
Mental	Overall	3.24 [2.22, 4.25]	1.13 [0.66, 1.6]	1.94 [0.79, 3.08]	2.69 [1.84, 3.53]
	High	3.09 [1.64, 4.54]	0.81 [0.18, 1.43]	1.83 [0.56, 3.1]	2.22 [0.9, 3.55]
	Low	3.36 [1.96, 4.76]	1.57 [0.88, 2.27]	2.35 [-0.23, 4.93]	3.18 [2.11, 4.25]
Neoplasms	Overall	4.39 [2.13, 6.65]	0.95 [-0.29, 2.18]	1.46 [-1.06, 3.99]	-0.06 [-2.16, 2.05]
	High	6.61 [3.29, 9.92]	0.33 [-1.36, 2.01]	1.28 [-1.6, 4.16]	0.81 [-2.44, 4.06]
	Low	2.36 [-0.51, 5.24]	1.35 [-0.29, 2.98]	2.12 [-2.12, 6.37]	-0.25 [-2.83, 2.33]
Endocrine	Overall	2.53 [1.24, 3.82]	1.85 [1.17, 2.53]	2.71 [1.16, 4.25]	2.41 [1.29, 3.52]
	High	3.06 [1.06, 5.05]	1.52 [0.62, 2.42]	2.7 [0.97, 4.44]	2.3 [0.68, 3.92]
	Low	2.05 [0.41, 3.68]	2.31 [1.33, 3.3]	2.73 [-0.38, 5.84]	2.22 [0.73, 3.71]
	Overall	2.49 [0.79, 4.19]	1.17 [0.27, 2.08]	2.47 [0.53, 4.41]	2.11 [0.68, 3.54]

Neurology	High	4.97 [2.38, 7.55]	0.89 [-0.31, 2.09]	2.39 [0.19, 4.59]	2.68 [0.58, 4.78]
	Low	0.22 [-1.96, 2.41]	1.28 [0.01, 2.56]	3.04 [-0.67, 6.76]	1.94 [0.08, 3.79]
Genitourinary	Overall	0.62 [-1.85, 3.09]	1.56 [0.08, 3.04]	0.8 [-2.28, 3.87]	-0.95 [-3.33, 1.42]
	High	3.92 [0.48, 7.36]	-0.09 [-2.07, 1.88]	1.47 [-2.08, 5.02]	-1.4 [-5.05, 2.24]
	Low	-2.33 [-5.55, 0.9]	2.93 [0.97, 4.9]	-1.95 [-6.14, 2.24]	0.17 [-2.58, 2.91]
CVD	Overall	-0.9 [-3.69, 1.89]	3.95 [2.27, 5.63]	-0.61 [-3.48, 2.27]	-4.96 [-7.73, -2.19]
	High	1.13 [-3.05, 5.31]	4.5 [2.21, 6.79]	-0.21 [-3.49, 3.07]	-0.57 [-4.23, 3.08]
	Low	-2.62 [-6, 0.75]	0.67 [-1.42, 2.76]	-2.33 [-6.61, 1.96]	-4.18 [-7.75, -0.62]

Return to normal period

Disease	Resilience	Storm	Flood	Tropical cyclone	Blizzard/Winter storm
Respiratory	Overall	-5.3 [-8.3, -2.31]	1.69 [0.13, 3.25]	1.28 [-1.58, 4.13]	-3.47 [-6.11, -0.84]
	High	-3.55 [-7.31, 0.21]	2.11 [0, 4.22]	1.16 [-2.14, 4.47]	1.23 [-2.54, 5.01]
	Low	-6.68 [-10.67, -2.69]	0.35 [-1.67, 2.38]	1.76 [-2.67, 6.19]	-9.33 [-12.52, -6.13]
Injuries	Overall	1.12 [-1.07, 3.31]	1.16 [-0.19, 2.5]	0.67 [-1.77, 3.11]	0.05 [-1.95, 2.05]
	High	-4.77 [-7.97, -1.57]	0.87 [-1.06, 2.81]	0.63 [-2.17, 3.44]	1.03 [-1.97, 4.02]
	Low	6.43 [3.64, 9.22]	0.44 [-1.14, 2.03]	0.89 [-3.16, 4.94]	-1.47 [-3.92, 0.98]
Infectious	Overall	1.12 [-1.07, 3.31]	0.46 [-0.51, 1.43]	1.27 [-0.57, 3.11]	0.77 [-0.62, 2.16]
	High	-4.77 [-7.97, -1.57]	0.6 [-0.57, 1.77]	1.26 [-0.86, 3.37]	1.02 [-1.2, 3.24]
	Low	6.43 [3.64, 9.22]	-0.32 [-1.8, 1.15]	1.3 [-1.89, 4.49]	0.63 [-1.05, 2.32]
Mental	Overall	1.34 [0.06, 2.63]	0.75 [0.29, 1.21]	0.56 [-0.49, 1.6]	0.45 [-0.28, 1.18]
	High	1.53 [-0.33, 3.4]	0.53 [-0.1, 1.15]	0.46 [-0.74, 1.66]	0.39 [-0.73, 1.51]
	Low	1.26 [-0.47, 2.98]	0.93 [0.29, 1.58]	0.95 [-1.02, 2.92]	0.45 [-0.5, 1.4]
Neoplasms	Overall	-1.21 [-3.63, 1.21]	0.98 [-0.25, 2.21]	0.46 [-1.98, 2.91]	-1.25 [-3.35, 0.85]
	High	-3.92 [-7.12, -0.72]	0.94 [-0.76, 2.65]	0.4 [-2.42, 3.22]	0.9 [-2.34, 4.13]
	Low	1.22 [-2.05, 4.48]	0.54 [-1.03, 2.12]	0.73 [-3.4, 4.87]	-4.3 [-6.87, -1.72]
Endocrine	Overall	0.71 [-0.72, 2.14]	0.7 [0.05, 1.35]	0.79 [-0.67, 2.24]	0.35 [-0.66, 1.37]
	High	0.25 [-2.32, 2.82]	0.58 [-0.32, 1.48]	0.84 [-0.82, 2.49]	1.09 [-0.44, 2.62]
	Low	1.3 [-0.1, 2.7]	0.62 [-0.27, 1.52]	0.6 [-2.16, 3.36]	-0.48 [-1.82, 0.87]
Neurology	Overall	3.19 [1.23, 5.15]	1.06 [0.19, 1.93]	1.17 [-0.71, 3.05]	-0.49 [-1.91, 0.93]
	High	-0.17 [-2.53, 2.2]	0.86 [-0.32, 2.05]	1.12 [-1.03, 3.27]	0.8 [-1.31, 2.91]
	Low	5.89 [3.04, 8.75]	1.03 [-0.16, 2.23]	1.52 [-2.1, 5.14]	-2.18 [-4.01, -0.35]
Genitourinary	Overall	-1.88 [-4.37, 0.61]	-0.11 [-1.56, 1.35]	0.71 [-2.31, 3.73]	-1.15 [-3.52, 1.23]
	High	-7.66 [-11.25, -4.08]	-0.12 [-2.11, 1.87]	0.49 [-3.04, 4.03]	3.61 [0.04, 7.18]
	Low	3.33 [0.23, 6.44]	-0.92 [-2.79, 0.94]	1.58 [-2.67, 5.83]	-6.56 [-9.37, -3.75]
CVD	Overall	-2.91 [-5.56, -0.26]	0.7 [-0.84, 2.25]	0.68 [-2.2, 3.55]	-1.17 [-3.73, 1.39]
	High	-4.23 [-7.98, -0.48]	1.07 [-0.98, 3.12]	1.04 [-2.31, 4.38]	2.39 [-1.23, 6.02]
	Low	-1.71 [-4.95, 1.54]	-0.34 [-2.36, 1.68]	-0.76 [-4.77, 3.25]	-5.15 [-8.26, -2.05]

CVD - cardiovascular disease

Table 9 | Sensitivity analysis of DID estimates without PSM.

Enduring period

Disease	Resilience	Storm	Flood	Tropical cyclone	Blizzard/Winter storm
Respiratory	Overall	11.21 [5.99, 16.43]	13.98 [12.13, 15.83]	13.44 [6.36, 20.52]	13.86 [9.43, 18.29]
Injuries	Overall	7.92 [3.41, 12.43]	11.76 [9.52, 14]	11.59 [5.87, 17.31]	9.52 [6.27, 12.77]
Infectious	Overall	-7.84 [-11.32, -4.36]	-10.49 [-12.91, -8.07]	8.14 [3.79, 12.49]	11.35 [8.94, 13.76]
Mental	Overall	-8.12 [-10.08, -6.16]	-10.03 [-11.34, -8.72]	-6.65 [-9.33, -3.97]	-8.13 [-9.22, -7.04]
Neoplasms	Overall	-10.56 [-13.28, -7.84]	-8.35 [-9.22, -7.48]	-11.71 [-17.48, -5.94]	2.75 [-0.91, 6.41]
Endocrine	Overall	-9.64 [-11.46, -7.82]	-13.28 [-13.99, -12.57]	-9.46 [-12.35, -6.57]	-7.23 [-9.15, -5.31]
Neurology	Overall	-9.42 [-13.47, -5.37]	-8.93 [-10.79, -7.07]	-9.97 [-14.33, -5.61]	-10.85 [-13.55, -8.15]
Genitourinary	Overall	-5.97 [-11.26, -0.68]	-14.11 [-15.37, -12.85]	-13.56 [-19.38, -7.74]	-12.02 [-16.17, -7.87]
CVD	Overall	10.53 [5.72, 15.34]	10.47 [8.21, 12.73]	13.72 [8.15, 19.29]	6.98 [2.54, 11.42]

CVD - cardiovascular disease

Stabilization-I period

Disease	Resilience	Storm	Flood	Tropical cyclone	Blizzard/Winter storm
Respiratory	Overall	6.72 [1.13, 12.31]	5.93 [4.13, 7.73]	7.71 [2.85, 12.57]	5.08 [1.02, 9.14]
Injuries	Overall	5.89 [1.25, 10.53]	4.77 [3.32, 6.22]	4.13 [0.07, 8.19]	3.75 [0.58, 6.92]
Infectious	Overall	8.12 [4.67, 11.57]	8.13 [6.84, 9.42]	6.35 [2.64, 10.06]	5.73 [3.62, 7.84]
Mental	Overall	6.64 [4.72, 8.56]	7.05 [6.14, 7.96]	4.82 [3.17, 6.47]	6.23 [5.17, 7.29]
Neoplasms	Overall	4.22 [-1.13, 9.57]	3.56 [2.09, 5.03]	4.99 [0.35, 9.63]	1.47 [-1.59, 4.53]
Endocrine	Overall	4.87 [2.05, 7.69]	5.63 [4.78, 6.48]	4.12 [1.64, 6.6]	4.23 [3.17, 5.29]
Neurology	Overall	5.33 [0.87, 9.79]	4.72 [3.48, 5.96]	5.33 [2.04, 8.62]	4.61 [2.49, 6.73]
Genitourinary	Overall	4.56 [0.03, 9.09]	6.73 [4.84, 8.62]	6.08 [0.57, 11.59]	2.38 [-1.65, 6.41]
CVD	Overall	4.82 [-1.11, 10.75]	5.09 [3.13, 7.05]	6.32 [1.73, 10.91]	1.38 [-2.77, 5.53]

CVD - cardiovascular disease

Stabilization-II period

Disease	Resilience	Storm	Flood	Tropical cyclone	Blizzard/Winter storm
Respiratory	Overall	1.66 [-2.02, 5.34]	1.23 [-0.77, 3.23]	1.89 [-3.45, 7.23]	2.03 [-3.41, 7.47]
Injuries	Overall	2.11 [-1.73, 5.95]	0.68 [-0.93, 2.29]	1.56 [-2.99, 6.11]	2.75 [-1.04, 6.54]
Infectious	Overall	3.38 [0.45, 6.31]	3.49 [2.23, 4.75]	2.97 [-1.23, 7.17]	4.02 [1.57, 6.47]
Mental	Overall	3.14 [1.69, 4.59]	2.02 [1.68, 2.36]	2.36 [0.54, 4.18]	2.84 [1.49, 4.19]
Neoplasms	Overall	2.29 [-1.53, 6.11]	2.21 [0.74, 3.68]	2.63 [-2.38, 7.64]	1.15 [-2.47, 4.77]
Endocrine	Overall	2.47 [0.42, 4.52]	3.38 [2.46, 4.3]	3.11 [0.27, 5.95]	2.69 [1.12, 4.26]
Neurology	Overall	2.84 [0.02, 5.66]	2.69 [1.47, 3.91]	2.84 [-2.77, 8.45]	2.04 [-0.33, 4.41]
Genitourinary	Overall	1.05 [-3.47, 5.57]	0.93 [-3.11, 4.97]	2.38 [-3.11, 7.87]	3.14 [-1.02, 7.3]
CVD	Overall	1.09 [-4.87, 7.05]	2.05 [0.17, 3.93]	6.32 [1.73, 10.91]	1.38 [-2.77, 5.53]

CVD - cardiovascular disease

Return to normal period

Disease	Resilience	Storm	Flood	Tropical cyclone	Blizzard/Winter storm
Respiratory	Overall	1.06 [-0.73, 2.85]	1.25 [-4.13, 6.63]	1.54 [-3.21, 6.29]	2.03 [-3.41, 7.47]
Injuries	Overall	0.23 [-1.45, 1.91]	0.68 [-4.34, 5.7]	2.42 [-1.13, 5.97]	2.75 [-1.04, 6.54]
Infectious	Overall	1.25 [-0.23, 2.73]	0.54 [-2.87, 3.95]	1.35 [-0.69, 3.39]	4.02 [1.57, 6.47]
Mental	Overall	0.75 [0.31, 1.19]	0.42 [-1.14, 1.98]	0.64 [-0.32, 1.6]	2.84 [1.49, 4.19]
Neoplasms	Overall	0.34 [-1.05, 1.73]	0.35 [-4.11, 4.81]	1.67 [-1.48, 4.82]	1.15 [-2.47, 4.77]
Endocrine	Overall	1.12 [0.32, 1.92]	0.42 [-1.97, 2.81]	1.21 [-0.43, 2.85]	2.69 [1.12, 4.26]
Neurology	Overall	1.26 [0.35, 2.17]	0.08 [-3.32, 3.48]	1.45 [-0.92, 3.82]	2.04 [-0.33, 4.41]
Genitourinary	Overall	0.57 [-1.52, 2.66]	0.21 [-5.89, 6.31]	2.42 [-1.93, 6.77]	4.05 [-1.02, 9.12]
CVD	Overall	1.42 [-0.44, 3.28]	0.58 [-5.11, 6.27]	1.49 [-2.96, 5.94]	1.38 [-2.77, 5.53]

CVD - cardiovascular disease

Table 10 | Representativeness of county-hazard exposures in the pandemic-excluded study period

Climate hazard	Events during pandemic period (%)	Distribution of excluded exposures by resilience level* (%)		Representativeness of pandemic-exluded period** (%)		
		High-resilience	Low-resilience	High-resilience	Low-resilience	Overall
Storms	23.5	42.6	57.4	100	100	100
Floods	15.5	52.7	47.3	100	100	100
Tropical cyclones	13.8	65.4	34.6	100	100	100
Blizzard/Winter storms	25.0	49.7	50.3	95.9	74.9	84.5

Note: * The proportion of constituting county-hazard combinations within high- and low-resilience subgroups during the pandemic period

** Representativeness calculated as the percentage of unique county-hazard combinations in the pandemic-excluded study period relative to all unique combinations across the entire study period. Values of 100% indicate complete geographic coverage is retained after pandemic exclusion.

Table 11 | Sensitivity analysis excluding the pandemic period.

Enduring period

Disease	Resilience	Storm	Flood	Tropical cyclone	Blizzard/Winter storm
	Overall	9.76 [4.83, 14.7]	11.97 [9.77, 14.17]	10.46 [3.86, 17.06]	13.47 [9.26, 17.67]
Respiratory	High	7.21 [0.67, 13.75]	10.18 [7.5, 12.85]	9.56 [2.63, 16.49]	12.15 [6.03, 18.27]
	Low	12.13 [5.2, 19.05]	15.68 [12.41, 18.96]	17.85 [4.42, 31.29]	15.87 [10.77, 20.98]
	Overall	7.91 [3.61, 12.21]	9.36 [7.6, 11.12]	8.81 [2.88, 14.75]	8.79 [5.3, 12.29]
Injuries	High	6.39 [0.5, 12.28]	8.47 [6.29, 10.64]	9.27 [3.03, 15.51]	7.98 [2.81, 13.16]
	Low	9.01 [3.03, 14.98]	11.33 [8.67, 13.99]	5.41 [-9.04, 19.86]	9.97 [5.62, 14.31]
	Overall	-8.16 [-11.4, -4.93]	-11.65 [-13.05, -10.25]	7.04 [2.06, 12.03]	11.35 [8.53, 14.17]
Infectious	High	-6.99 [-11.61, -2.37]	-10.47 [-12.21, -8.74]	6.41 [1.15, 11.67]	8.94 [4.78, 13.11]
	Low	-9.07 [-13.46, -4.69]	-13.07 [-15.24, -10.91]	12.68 [-1.42, 26.77]	14.22 [10.63, 17.8]
	Overall	-7.58 [-10.01, -5.15]	-10.39 [-11.35, -9.42]	-6.46 [-9.72, -3.2]	-8.53 [-10.37, -6.7]
Mental	High	-5.52 [-8.63, -2.41]	-8.69 [-9.86, -7.51]	-6.53 [-9.97, -3.09]	-6.13 [-8.94, -3.32]
	Low	-8.74 [-12.19, -5.3]	-12.73 [-14.32, -11.13]	-6.27 [-16.94, 4.39]	-10.7 [-13.06, -8.34]
	Overall	-10.28 [-14.61, -5.96]	-8.91 [-10.69, -7.12]	-10.58 [-17.16, -3.99]	2.82 [-0.73, 6.38]
Neoplasms	High	-8.13 [-14, -2.26]	-7.56 [-9.78, -5.35]	-10.17 [-17.16, -3.18]	1.71 [-3.62, 7.04]
	Low	-12.3 [-18.29, -6.32]	-10.6 [-13.27, -7.93]	-15.07 [-29.5, -0.63]	3.94 [-0.5, 8.39]
	Overall	-9.81 [-12.6, -7.02]	-11.49 [-12.66, -10.32]	-9.81 [-14.1, -5.52]	-7.07 [-9.32, -4.82]
Endocrine	High	-7.96 [-11.95, -3.97]	-10.32 [-11.75, -8.89]	-9.39 [-13.95, -4.83]	-5.29 [-8.41, -2.16]
	Low	-10.87 [-14.66, -7.08]	-13.11 [-15.01, -11.2]	-13.12 [-24.81, -1.42]	-8.78 [-11.91, -5.66]
	Overall	-10.08 [-13.6, -6.55]	-10.67 [-12.09, -9.26]	-8.4 [-13.09, -3.7]	-10.14 [-12.7, -7.57]
Neurology	High	-8.48 [-13.63, -3.34]	-9.9 [-11.66, -8.15]	-7.33 [-12.15, -2.51]	-8.62 [-12.44, -4.8]
	Low	-11.02 [-15.75, -6.28]	-11.71 [-13.91, -9.5]	-17.29 [-33.04, -1.54]	-11.35 [-14.61, -8.1]
	Overall	-7 [-11.48, -2.52]	-15.08 [-17.12, -13.04]	-12.39 [-18.96, -5.81]	-13.43 [-17.32, -9.53]
Genitourinary	High	-4.96 [-11.05, 1.14]	-14.09 [-16.6, -11.57]	-12.21 [-19.2, -5.22]	-11.24 [-17.24, -5.23]
	Low	-8.59 [-14.57, -2.6]	-15.85 [-18.89, -12.82]	-16.07 [-30.1, -2.05]	-14.12 [-18.76, -9.47]
	Overall	8.87 [3.98, 13.76]	10.07 [7.93, 12.21]	12.17 [5.64, 18.69]	5.85 [1.85, 9.86]
CVD	High	7.15 [0.63, 13.66]	9.51 [6.93, 12.1]	12.46 [5.62, 19.29]	5.42 [-0.42, 11.26]
	Low	10.26 [3.51, 17.02]	11.17 [7.99, 14.36]	10.87 [-2.26, 24]	7.93 [3.01, 12.85]

Stabilization-I period

Disease	Resilience	Storm	Flood	Tropical cyclone	Blizzard/Winter storm
	Overall	6.21 [0.37, 12.05]	6.04 [4.1, 7.97]	4.97 [0.05, 9.9]	4.42 [-0.02, 8.87]
Respiratory	High	5.03 [-1.78, 11.85]	5.99 [3.46, 8.52]	2.09 [-3.81, 7.99]	4.91 [-0.74, 10.55]
	Low	7.1 [-1.32, 15.52]	6.95 [4.19, 9.72]	5.2 [-0.87, 11.26]	8.79 [3.2, 14.38]
	Overall	5.36 [0.53, 10.18]	4.67 [3.14, 6.21]	1.59 [-2.59, 5.77]	3.1 [-0.34, 6.54]
Injuries	High	3.49 [-2.43, 9.41]	4.33 [2.33, 6.32]	-0.09 [-5.05, 4.86]	2.47 [-2.02, 6.97]
	Low	6.08 [-0.69, 12.85]	5.96 [3.67, 8.25]	3.04 [-2.36, 8.44]	4.72 [0.69, 8.75]
	Overall	7.59 [3.91, 11.27]	7.84 [6.61, 9.07]	5.1 [1.66, 8.55]	5.58 [2.94, 8.21]
Infectious	High	6.69 [2.62, 10.76]	7.3 [5.74, 8.86]	3.97 [0.02, 7.92]	5.57 [2.2, 8.95]

	Low	8.18 [3.18, 13.19]	9.17 [7.32, 11.03]	6.43 [1.54, 11.32]	6.35 [3.09, 9.6]
Mental	Overall	6.38 [4.36, 8.4]	6.5 [5.72, 7.29]	3.47 [1.86, 5.08]	5.75 [4.48, 7.03]
	High	5.42 [3.17, 7.67]	5.88 [4.89, 6.87]	3.32 [1.56, 5.07]	5.08 [3.47, 6.69]
	Low	6.6 [4.06, 9.14]	7.38 [6.17, 8.59]	3.34 [0.24, 6.43]	6.62 [4.99, 8.25]
Neoplasms	Overall	3.66 [-1.92, 9.25]	3.51 [1.95, 5.06]	3.17 [-0.95, 7.28]	0.74 [-2.74, 4.22]
	High	2.42 [-4.39, 9.22]	3.49 [1.43, 5.54]	1.11 [-3.78, 5.99]	-0.52 [-5.2, 4.15]
	Low	4.4 [-3.38, 12.19]	5.02 [2.77, 7.27]	2.97 [-2.04, 7.98]	2.14 [-2.07, 6.34]
Endocrine	Overall	4.46 [1.61, 7.3]	5.45 [4.52, 6.39]	3.18 [0.77, 5.59]	3.88 [2.02, 5.74]
	High	3.58 [0.1, 7.07]	4.87 [3.65, 6.09]	2.94 [0.29, 5.6]	3.53 [1.32, 5.75]
	Low	5.66 [2.32, 9]	6.56 [5.15, 7.97]	4.4 [0.33, 8.47]	4.58 [2.36, 6.81]
Neurology	Overall	5.04 [0.6, 9.47]	4.65 [3.45, 5.85]	4.03 [0.7, 7.36]	4.07 [1.74, 6.4]
	High	3.64 [-0.66, 7.94]	4.43 [2.86, 6]	2.87 [-0.98, 6.71]	3.44 [0.6, 6.28]
	Low	4.73 [-1.77, 11.23]	5.13 [3.37, 6.9]	2.79 [-2.03, 7.61]	5.2 [2.44, 7.96]
Genitourinary	Overall	4.11 [-1.08, 9.29]	6.99 [5.2, 8.79]	2.96 [-1.97, 7.89]	2.03 [-2.04, 6.1]
	High	3.75 [-2.51, 10.01]	6.44 [4.07, 8.81]	-0.47 [-6.39, 5.44]	2.73 [-2.88, 8.34]
	Low	4.55 [-2.77, 11.87]	8.13 [5.56, 10.71]	4.67 [-0.73, 10.07]	7.03 [2.02, 12.04]
CVD	Overall	4.4 [-1.86, 10.67]	4.73 [2.86, 6.6]	3.79 [-1.14, 8.73]	-0.56 [-4.65, 3.54]
	High	4.17 [-3.3, 11.64]	4.49 [2.09, 6.9]	-0.11 [-6.03, 5.8]	1.83 [-3.64, 7.29]
	Low	4.27 [-4.61, 13.14]	6.04 [3.31, 8.78]	3.79 [-2.02, 9.6]	3.83 [-1.1, 8.75]

Stabilization-II period

Disease	Resilience	Storm	Flood	Tropical cyclone	Blizzard/Winter storm
Respiratory	Overall	1.45 [-3.06, 5.95]	1.27 [-0.91, 3.44]	0.28 [-4.93, 5.49]	0.43 [-4.95, 5.81]
	High	2.01 [-3.42, 7.43]	3.39 [0.57, 6.21]	-0.53 [-6.73, 5.68]	-0.69 [-7.52, 6.15]
	Low	2.7 [-3.85, 9.26]	3.31 [0.38, 6.24]	2.25 [-4.25, 8.75]	2.74 [-3.69, 9.17]
Injuries	Overall	1.8 [-1.99, 5.58]	-0.44 [-2.27, 1.39]	0.34 [-4.11, 4.79]	1.44 [-2.55, 5.44]
	High	1.1 [-3.69, 5.9]	1.08 [-1.24, 3.4]	-0.52 [-5.77, 4.74]	-0.12 [-4.82, 4.58]
	Low	1.99 [-3.47, 7.44]	0.32 [-2.44, 3.08]	2.02 [-3.34, 7.38]	2.5 [-1.62, 6.63]
Infectious	Overall	3.06 [0.19, 5.94]	3 [1.83, 4.17]	2.3 [-1.83, 6.43]	4.2 [1.68, 6.73]
	High	2.51 [-1.14, 6.17]	3.02 [1.56, 4.48]	1.63 [-3.24, 6.49]	4.67 [1.15, 8.2]
	Low	4.02 [0.04, 8]	3.63 [1.88, 5.38]	2.73 [-1.75, 7.22]	3.65 [0.72, 6.57]
Mental	Overall	2.96 [1.43, 4.49]	1.83 [1.19, 2.47]	1.81 [0.39, 3.23]	2.47 [1.26, 3.69]
	High	2.6 [0.87, 4.32]	1.65 [0.86, 2.44]	1.73 [0.21, 3.25]	2.22 [0.66, 3.78]
	Low	3.64 [1.56, 5.72]	1.84 [0.87, 2.82]	2.06 [-0.92, 5.05]	3.06 [1.61, 4.51]
Neoplasms	Overall	2.06 [-1.87, 5.98]	1.89 [0.27, 3.51]	1.72 [-2.79, 6.23]	-0.09 [-4.22, 4.04]
	High	1.91 [-3.15, 6.97]	2.19 [0.12, 4.27]	0.87 [-4.46, 6.21]	-1.36 [-6.53, 3.8]
	Low	2.85 [-2.62, 8.33]	2.48 [0.1, 4.87]	2.35 [-2.72, 7.41]	1.13 [-3.34, 5.61]
Endocrine	Overall	2.26 [0.04, 4.49]	2.94 [2.03, 3.86]	2.1 [-0.29, 4.48]	2.38 [0.48, 4.28]
	High	1.88 [-1.04, 4.8]	2.66 [1.5, 3.81]	2.22 [-0.39, 4.82]	1.84 [-0.15, 3.82]
	Low	2.83 [0.1, 5.55]	2.82 [1.43, 4.22]	2.16 [-1.43, 5.74]	2.76 [0.57, 4.96]
	Overall	2.51 [-0.46, 5.47]	2.31 [1.11, 3.51]	2.36 [-0.98, 5.69]	1.32 [-1.22, 3.87]

Neurology	High	1.55 [-2.32, 5.41]	2 [0.47, 3.53]	1.82 [-2.01, 5.65]	0.97 [-1.91, 3.85]
	Low	1.99 [-1.95, 5.94]	2.51 [0.72, 4.31]	2.18 [-2.55, 6.92]	3.31 [0.45, 6.16]
Genitourinary	Overall	0.57 [-3.58, 4.71]	2.06 [0.22, 3.9]	1.52 [-3.82, 6.86]	3.05 [-1.7, 7.79]
	High	1.84 [-3.34, 7.03]	2.55 [0.22, 4.89]	0.11 [-6.33, 6.55]	-1.07 [-7.76, 5.62]
	Low	2.14 [-3.82, 8.11]	3.37 [0.64, 6.09]	0.79 [-4.23, 5.82]	3.5 [-1.92, 8.91]
CVD	Overall	-0.8 [-5.42, 3.83]	1.64 [-0.49, 3.77	0.96 [-4.27, 6.19]	1.36 [-4.11, 6.84]
	High	1.02 [-4.99, 7.03]	2.65 [-0.07, 5.38]	0.09 [-6.11, 6.3]	-2.76 [-9.44, 3.93]
	Low	2.31 [-3.97, 8.58]	2.79 [-0.14, 5.72]	2.15 [-4.39, 8.69]	0.52 [-6.53, 7.57]

Return to normal period

Disease	Resilience	Storm	Flood	Tropical cyclone	Blizzard/Winter storm
Respiratory	Overall	1.78 [-3.47, 7.03]	1.31 [-0.56, 3.19]	1.49 [-4.3, 7.27]	1.29 [-3.8, 6.38]
	High	-1.16 [-7.08, 4.75]	1.32 [-1.16, 3.8]	1.26 [-5.61, 8.13]	1.19 [-4.8, 7.18]
	Low	-2.81 [-10.5, 4.87]	1.87 [-0.71, 4.45]	2.39 [-4.41, 9.19]	2.16 [-4.06, 8.38]
Injuries	Overall	1.71 [-2.32, 5.74]	-0.01 [-1.68, 1.66]	1.31 [-3.71, 6.32]	2.26 [-1.57, 6.09]
	High	-1.58 [-6.92, 3.75]	-0.11 [-2.44, 2.21]	0.84 [-5.08, 6.77]	0.01 [-4.94, 4.96]
	Low	0.31 [-5.11, 5.74]	0.79 [-1.31, 2.89]	1.67 [-4.04, 7.38]	-0.59 [-4.9, 3.72]
Infectious	Overall	-0.31 [-3.79, 3.17]	1.09 [-0.16, 2.34]	0.55 [-3.16, 4.25]	1.79 [-0.6, 4.18]
	High	-0.5 [-4.96, 3.96]	1.35 [-0.13, 2.83]	1.01 [-3.28, 5.3]	1.47 [-1.88, 4.83]
	Low	-2.79 [-7.51, 1.93]	1.56 [-0.43, 3.55]	0.38 [-3.76, 4.52]	1.1 [-1.48, 3.69]
Mental	Overall	0.89 [-1.31, 3.09]	0.58 [-0.01, 1.18]	0.3 [-1.24, 1.84]	0.67 [-0.64, 1.99]
	High	0.55 [-2, 3.1]	0.46 [-0.34, 1.25]	0.36 [-1.28, 2]	0.26 [-1.27, 1.79]
	Low	1.25 [-1.38, 3.88]	0.73 [-0.11, 1.57]	0.7 [-1.32, 2.73]	0.59 [-0.53, 1.72]
Neoplasms	Overall	0.99 [-3.43, 5.4]	-0.01 [-1.55, 1.52]	1.43 [-3.42, 6.27]	1.46 [-2.49, 5.4]
	High	-2.83 [-8.14, 2.48]	0.75 [-1.32, 2.82]	0.96 [-4.77, 6.7]	-1.06 [-5.98, 3.87]
	Low	-0.33 [-6.65, 5.98]	0.39 [-1.72, 2.5]	1.72 [-3.85, 7.29]	-1.43 [-5.86, 3]
Endocrine	Overall	1.1 [-1.44, 3.63]	0.71 [-0.13, 1.55]	0.77 [-1.72, 3.26]	0.97 [-0.76, 2.7]
	High	-1.67 [-5.76, 2.42]	0.93 [-0.2, 2.07]	0.52 [-2.2, 3.25]	0.6 [-1.58, 2.78]
	Low	0.52 [-1.76, 2.81]	0.37 [-0.82, 1.56]	0.79 [-2.35, 3.93]	0.37 [-1.58, 2.32]
Neurology	Overall	0.98 [-2.6, 4.57]	1.15 [0.04, 2.26]	0.21 [-3.15, 3.57]	1.17 [-1.35, 3.68]
	High	0.5 [-3.32, 4.31]	1.07 [-0.43, 2.56]	0.77 [-3.02, 4.57]	0.02 [-3.05, 3.1]
	Low	-0.03 [-5.4, 5.33]	0.95 [-0.61, 2.52]	-0.55 [-5.22, 4.12]	0.07 [-2.71, 2.84]
Genitourinary	Overall	2.47 [-2.01, 6.95]	0.11 [-1.62, 1.85]	0.64 [-5.18, 6.45]	2.4 [-2.21, 7.01]
	High	-2.66 [-8.52, 3.2]	0.57 [-1.76, 2.9]	-0.1 [-7.12, 6.92]	-1.21 [-7.41, 5]
	Low	-2.32 [-8.36, 3.71]	1.18 [-1.2, 3.56]	2.1 [-3.26, 7.47]	-1.19 [-6.73, 4.36]
CVD	Overall	2.03 [-2.7, 6.77]	0.92 [-0.94, 2.78]	1.06 [-4.7, 6.81]	1.03 [-3.91, 5.97]
	High	-3.51 [-9.45, 2.44]	1.38 [-1.04, 3.8]	1.09 [-5.81, 7.99]	-3.47 [-9.46, 2.52]
	Low	-3.77 [-10.09, 2.56]	1.38 [-1.23, 4]	2.06 [-4.83, 8.94]	-0.9 [-6.93, 5.14]

CVD - cardiovascular disease

Table 12 | Age-stratification analysis following climate hazards in China during 2016-2023.

Age group	Storm	Flood	Tropical cyclone	Blizzard/Winter storm
**Enduring**				
≤18	-8.39 [-10.31, -6.47]	-8.25 [-9.47, -7.03]	-5.82 [-7.13, -4.51]	-3.85 [-5.32, -2.38]
19-44	-6.26 [-8.69, -3.83]	-8.31 [-10.15, -6.47]	-4.78 [-6.53, -3.03]	-4.29 [-5.73, -2.85]
45-64	-7.23 [-8.45, -6.01]	-9.67 [-11.53, -7.81]	-5.99 [-7.84, -4.14]	-4.76 [-6.11, -3.41]
≥65	-10.62 [-14.38, -6.86]	-12.48 [-15.32, -9.64]	-7.39 [-10.42, -4.36]	-5.38 [-7.92, -2.84]
**Stabilization-I**				
≤18	5.03 [3.69, 6.37]	7.15 [6.42, 7.88]	5.84 [3.26, 8.42]	4.22 [2.16, 6.28]
19-44	4.66 [3.28, 6.04]	4.39 [3.74, 5.04]	4.87 [3.52, 6.22]	3.07 [1.35, 4.79]
45-64	5.95 [3.47, 8.43]	5.18 [4.56, 5.8]	6.42 [4.35, 8.49]	4.42 [2.16, 6.68]
≥65	6.84 [4.13, 9.55]	9.83 [7.26, 12.4]	7.39 [5.78, 9]	5.13 [2.67, 7.59]
**Stabilization-II**				
≤18	2.52 [1.21, 3.83]	2.89 [1.64, 4.14]	2.32 [1.01, 3.63]	1.98 [0.75, 3.21]
19-44	1.48 [0.87, 2.09]	2.06 [0.57, 3.55]	1.98 [0.74, 3.22]	1.67 [0.24, 3.1]
45-64	1.95 [1.05, 2.85]	3.13 [1.07, 5.19]	3.34 [1.62, 5.06]	2.64 [0.52, 4.76]
≥65	3.46 [0.69, 6.23]	3.52 [1.21, 5.83]	3.78 [1.68, 5.88]	3.26 [1.05, 5.47]
**Return to normal**				
≤18	1.46 [-0.69, 3.61]	1.34 [-0.32, 3]	0.47 [-0.77, 1.71]	1.48 [-0.26, 3.22]
19-44	0.83 [-0.24, 1.9]	0.45 [-0.56, 1.46]	0.12 [-0.59, 0.83]	1.32 [-0.58, 3.22]
45-64	1.93 [-0.89, 4.75]	1.66 [-0.77, 4.09]	0.62 [-1.03, 2.27]	1.75 [-1.53, 5.03]
≥65	2.19 [-1.21, 5.59]	1.78 [-1.12, 4.68]	1.22 [-1.36, 3.8]	2.08 [-1.77, 5.93]

Table 13 | Relative changes of cause-specific hospitalization rates in disaster-exposed regions at warning period.

Disease	Resilience	Storm	Flood	Tropical cyclone	Blizzard/Winter storm
Respiratory	Overall	-2.13 [-13.73, 9.46]	0.6 [-3.08, 4.28]	3.04 [-5.89, 11.97]	-0.29 [-10.72, 10.15]
	High	-1.63 [-19.14, 15.88]	2.16 [-2.99, 7.31]	3.66 [-5.98, 13.3]	5.59 [-7.02, 18.19]
	Low	-3.73 [-18.02, 10.57]	0.77 [-4.56, 6.1]	-8.58 [-28.14, 10.99]	-4.55 [-16.44, 7.33]
Injuries	Overall	-4.49 [-13.89, 4.91]	-1.22 [-4.02, 1.58]	2.18 [-5.64, 9.99]	1.21 [-5.98, 8.4]
	High	-0.95 [-14.84, 12.93]	0.74 [-3.13, 4.61]	1.74 [-6.87, 10.34]	2.55 [-6.87, 11.96]
	Low	-7.62 [-18.51, 3.27]	-0.77 [-4.79, 3.26]	-7.96 [-21.97, 6.04]	0.92 [-5.81, 7.66]
Infectious	Overall	0.38 [-6.01, 6.77]	0.79 [-1.27, 2.85]	1.85 [-3.65, 7.35]	0.67 [-4.94, 6.28]
	High	1.87 [-5.66, 9.39]	1.49 [-1.2, 4.18]	1.02 [-5.18, 7.22]	1.79 [-5.14, 8.72]
	Low	-1.9 [-10.03, 6.24]	1.33 [-1.77, 4.42]	-1.17 [-10.43, 8.09]	1.79 [-3.96, 7.54]
Mental	Overall	1.17 [-1.09, 3.42]	0.37 [-0.66, 1.4]	0.86 [-2.22, 3.94]	0.92 [-1.19, 3.03]
	High	1.41 [-1.43, 4.25]	0.12 [-1.26, 1.49]	1.02 [-2.46, 4.5]	0.94 [-2.14, 4.02]
	Low	0.81 [-1.44, 3.06]	0.9 [-0.51, 2.31]	0.19 [-4.52, 4.89]	1.38 [-0.29, 3.05]
Neoplasms	Overall	-3.28 [-12.82, 6.25]	-1.32 [-4.21, 1.56]	1.47 [-6.23, 9.16]	0.16 [-7.66, 7.97]
	High	-0.7 [-14.32, 12.92]	-0.35 [-4.35, 3.65]	0.1 [-8.38, 8.57]	2.74 [-7.05, 12.52]
	Low	-4.69 [-16, 6.62]	-1.35 [-5.52, 2.81]	-2.62 [-17.48, 12.25]	0.11 [-7.57, 7.79]
Endocrine	Overall	1.21 [-2.89, 5.31]	0.48 [-1.22, 2.19]	1.04 [-3.33, 5.41]	-0.52 [-4.83, 3.79]
	High	1.57 [-4, 7.13]	0.95 [-1.38, 3.28]	0.74 [-4.12, 5.61]	0.02 [-5.56, 5.59]
	Low	1.12 [-3.06, 5.31]	0.94 [-1.44, 3.32]	0.61 [-7.22, 8.43]	-0.13 [-3.43, 3.18]
Neurology	Overall	-0.91 [-7.71, 5.88]	-0.59 [-2.79, 1.61]	2.33 [-4.03, 8.69]	0.71 [-4.24, 5.66]
	High	1 [-7.26, 9.26]	0.67 [-2.25, 3.58]	1.07 [-6.07, 8.21]	1.9 [-4.25, 8.05]
	Low	-2.56 [-10.84, 5.72]	-0.72 [-3.98, 2.53]	0.44 [-10.17, 11.06]	0.4 [-5.36, 6.16]
Genitourinary	Overall	-4.03 [-14.68, 6.62]	-0.52 [-3.9, 2.86]	3.72 [-5.44, 12.87]	2.04 [-8.8, 12.88]
	High	-4.24 [-20.2, 11.72]	0.75 [-4.11, 5.6]	2.14 [-8.23, 12.51]	5.39 [-8.47, 19.25]
	Low	-5.53 [-18.39, 7.34]	-0.4 [-5.13, 4.34]	-3.54 [-21.27, 14.19]	1 [-9.08, 11.07]
CVD	Overall	-3.93 [-15.13, 7.26]	0.77 [-2.89, 4.43]	2.7 [-6.61, 12.02]	-2.49 [-12.59, 7.61]
	High	-0.8 [-17.67, 16.06]	2.92 [-2.02, 7.86]	3.12 [-7.28, 13.53]	2.7 [-10.02, 15.42]
	Low	-5.22 [-18.66, 8.21]	0.9 [-4.58, 6.37]	-13.62 [-29.67, 2.43]	0.69 [-8.78, 10.16]

CVD - cardiovascular disease

Table 14 | Relative changes of cause-specific hospitalization rates in neighboring regions (0-200 km) at warning period.

Disease	Resilience	Storm	Flood	Tropical cyclone	Blizzard/Winter storm
Respiratory	Overall	-1.59 [-11.8, 8.62]	-0.9 [-6.12, 4.32]	3.37 [-4.57, 11.32]	0.67 [-10.1, 11.45]
	High	2.18 [-10.71, 15.06]	-2.29 [-9.44, 4.86]	1.88 [-9.05, 12.81]	2.31 [-10.23, 14.84]
	Low	3.68 [-8.94, 16.3]	0.01 [-6.67, 6.68]	2.53 [-7.93, 12.98]	2.87 [-10.35, 16.09]
Injuries	Overall	-1.6 [-9.27, 6.08]	0.25 [-3.88, 4.38]	1.69 [-5.31, 8.68]	1.06 [-6.53, 8.64]
	High	-0.13 [-8.6, 8.34]	-0.41 [-6.05, 5.24]	-0.4 [-10.48, 9.68]	2.06 [-7.21, 11.34]
	Low	1.24 [-9.38, 11.86]	2.08 [-3.23, 7.4]	0.81 [-8.18, 9.79]	0.5 [-9.43, 10.42]
Infectious	Overall	1.4 [-4.5, 7.3]	-0.86 [-4.18, 2.47]	-1.22 [-7.21, 4.77]	1.57 [-2.94, 6.08]
	High	2.02 [-4.73, 8.77]	-0.31 [-4.76, 4.14]	-0.88 [-9.66, 7.9]	1 [-4.53, 6.54]
	Low	2.39 [-4.48, 9.26]	0.04 [-4.37, 4.45]	-3.39 [-11.21, 4.42]	2.6 [-3.89, 9.1]
Mental	Overall	0.45 [-3.17, 4.06]	0.59 [-1.41, 2.59]	0.32 [-3.39, 4.02]	-0.31 [-3.84, 3.21]
	High	1.62 [-1.93, 5.16]	-0.78 [-3.23, 1.68]	1.21 [-3.69, 6.1]	0.09 [-4.62, 4.8]
	Low	0.41 [-4.11, 4.94]	2.57 [0.25, 4.89]	-2.43 [-7.22, 2.36]	-0.95 [-5.74, 3.83]
Neoplasms	Overall	-0.81 [-9.16, 7.55]	1.18 [-2.76, 5.11]	2.19 [-5.46, 9.84]	2.01 [-7.19, 11.2]
	High	1.3 [-8.16, 10.76]	1.03 [-4.38, 6.43]	1.36 [-9.69, 12.41]	1.88 [-8.78, 12.53]
	Low	3.74 [-7.64, 15.12]	2.47 [-2.59, 7.52]	-0.44 [-10.33, 9.45]	4.83 [-8.45, 18.12]
Endocrine	Overall	1.67 [-2.88, 6.22]	2.67 [-0.2, 5.54]	2.64 [-2.17, 7.46]	-1.51 [-7.58, 4.57]
	High	1.86 [-3.61, 7.34]	2.58 [-1.13, 6.28]	0.4 [-5.85, 6.64]	-1.14 [-7.92, 5.65]
	Low	2.14 [-2.63, 6.92]	3.73 [-0.09, 7.55]	3.05 [-3.7, 9.79]	-1.59 [-10.01, 6.82]
Neurology	Overall	0.52 [-5.24, 6.27]	0.94 [-2.65, 4.53]	0.87 [-5.75, 7.49]	1.6 [-5.33, 8.53]
	High	1.68 [-4.1, 7.47]	0.3 [-4.57, 5.17]	-1.61 [-11.19, 7.98]	1.45 [-5.19, 8.08]
	Low	3.61 [-5.39, 12.61]	2.18 [-2.47, 6.83]	-1.3 [-9.8, 7.21]	2.27 [-9.28, 13.81]
Genitourinary	Overall	-0.8 [-9.85, 8.25]	-0.25 [-5.21, 4.71]	3.24 [-5.58, 12.05]	0.77 [-9.88, 11.42]
	High	2.05 [-8.67, 12.77]	-3.22 [-10.16, 3.73]	2.64 [-10.02, 15.29]	0.68 [-11.44, 12.79]
	Low	3.96 [-8.08, 15.99]	3.23 [-3.05, 9.5]	-0.3 [-11.61, 11.01]	3.83 [-11.32, 18.97]
CVD	Overall	-0.5 [-9.94, 8.93]	1.48 [-3.14, 6.1]	2.61 [-6.19, 11.41]	1.5 [-7.72, 10.73]
	High	2.14 [-9.45, 13.74]	0.27 [-6.02, 6.55]	-1.89 [-13.77, 9.99]	2.37 [-9.07, 13.8]
	Low	2.57 [-9.12, 14.26]	3.3 [-2.6, 9.2]	1.34 [-10.44, 13.12]	2.22 [-8.88, 13.32]

CVD - cardiovascular disease

Table 15 | Relative changes of cause-specific hospitalization rates in neighboring regions (0-100 km) in western China.

Enduring period

Disease	Resilience	Storm	Flood	Blizzard/Winter storm
Respiratory	Overall	5.82 [-3.01, 14.65]	6.43 [2.35, 10.51]	7.69 [-5.99, 21.37]
Injuries	Overall	2.76 [-5.97, 11.49]	4.42 [0.89, 7.95]	4.39 [-7.15, 15.93]
Infectious	Overall	5.55 [-0.36, 11.46]	4.64 [2.03, 7.25]	7.43 [-2.74, 17.6]
Mental	Overall	4.29 [0.51, 8.07]	2.57 [1.11, 4.03]	5.06 [-1.52, 11.64]
Neoplasms	Overall	2.33 [-4.1, 8.76]	4.36 [0.51, 8.21]	2.49 [-7.17, 12.15]
Endocrine	Overall	4.36 [0.03, 8.69]	2.94 [0.32, 5.56]	4.98 [-2.76, 12.72]
Neurology	Overall	3.92 [-2.31, 10.15]	4.35 [1.23, 7.47]	2.42 [-5.47, 10.31]
Genitourinary	Overall	3.54 [-4.79, 11.87]	4.87 [0.77, 8.97]	5.21 [-5.91, 16.33]
CVD	Overall	6.35 [-2.37, 15.07]	7.42 [3.36, 11.48]	5.63 [-6.51, 17.77]

Stabilization-I period

Disease	Resilience	Storm	Flood	Blizzard/Winter storm
Respiratory	Overall	5.34 [-4.12, 14.8]	5.96 [1.87, 10.05]	4.63 [-5.35, 14.61]
Injuries	Overall	2.31 [-5.54, 10.16]	3.64 [0.41, 6.87]	3.21 [-4.76, 11.18]
Infectious	Overall	5.14 [1.35, 8.93]	4.46 [2.32, 6.6]	5.23 [-0.41, 10.87]
Mental	Overall	3.73 [1.23, 6.23]	3.32 [1.89, 4.75]	4.57 [0.91, 8.23]
Neoplasms	Overall	2.93 [-4.04, 9.9]	4.61 [1.23, 7.99]	1.78 [-5.36, 8.92]
Endocrine	Overall	3.76 [0.07, 7.45]	2.22 [0.41, 4.03]	4.48 [-0.88, 9.84]
Neurology	Overall	3.64 [-1.52, 8.8]	3.56 [1.13, 5.99]	2.68 [-2.12, 7.48]
Genitourinary	Overall	3.28 [-4.81, 11.37]	4.58 [0.64, 8.52]	4.48 [-5.87, 14.83]
CVD	Overall	5.23 [-4.47, 14.93]	4.65 [0.33, 8.97]	4.01 [-4.36, 12.38]

Stabilization-II period

Disease	Resilience	Storm	Flood	Blizzard/Winter storm
Respiratory	Overall	4.02 [-4.21, 12.25]	2.94 [-1.32, 7.2]	1.63 [-8.1, 11.36]
Injuries	Overall	2.75 [-3.41, 8.91]	0.34 [-2.84, 3.52]	2.31 [-4.67, 9.29]
Infectious	Overall	3.57 [-1.21, 8.35]	3.41 [1.39, 5.43]	2.99 [-2.3, 8.28]
Mental	Overall	2.55 [0.31, 4.79]	2.42 [1.24, 3.6]	1.86 [-2.35, 6.07]
Neoplasms	Overall	2.23 [-4.23, 8.69]	2.21 [-1.23, 5.65]	0.74 [-7.11, 8.59]
Endocrine	Overall	1.63 [-2.02, 5.28]	1.54 [-0.34, 3.42]	1.93 [-2.86, 6.72]
Neurology	Overall	2.34 [-2.14, 6.82]	2.51 [0.38, 4.64]	1.37 [-3.88, 6.62]
Genitourinary	Overall	3.13 [-4.36, 10.62]	3.37 [-0.78, 7.52]	2.63 [-6.45, 11.71]
CVD	Overall	0.77 [-7.33, 8.87]	1.98 [-2.14, 6.1]	3.41 [-4.99, 11.81]

Return to normal period

Disease	Resilience	Storm	Flood	Blizzard/Winter storm
Respiratory	Overall	0.39 [-8.54, 9.32]	0.62 [-3.65, 4.89]	-0.05 [-9.89, 9.79]
Injuries	Overall	-1.08 [-8.78, 6.62]	0.22 [-2.93, 3.37]	0.36 [-6.89, 7.61]

Infectious	Overall	1.39 [-3.03, 5.81]	1.25 [-1.68, 4.18]	0.13 [-5.37, 5.63]
Mental	Overall	0.93 [-1.73, 3.59]	0.21 [-1.75, 2.17]	0.62 [-2.67, 3.91]
Neoplasms	Overall	-0.42 [-6.68, 5.84]	-0.52 [-4.73, 3.69]	0.58 [-8.41, 9.57]
Endocrine	Overall	0.88 [-1.89, 3.65]	1.21 [-0.35, 2.77]	1.35 [-4.76, 7.46]
Neurology	Overall	1.04 [-2.45, 4.53]	0.03 [-2.38, 2.44]	0.74 [-4.52, 6]
Genitourinary	Overall	0.74 [-7.32, 8.8]	1.34 [-3.14, 5.82]	-0.93 [-9.35, 7.49]
CVD	Overall	1.82 [-6.63, 10.27]	1.03 [-3.03, 5.09]	-0.56 [-8.36, 7.24]

Note: the western and eastern subregions are defined by the Hu Huanyong Line[83]. Tropical cyclones were not applicable for western China.

Table 16 | Relative changes of cause-specific hospitalization rates in neighboring regions (100-200 km) in western China.

Enduring period

Disease	Resilience	Storm	Flood	Blizzard/Winter storm
Respiratory	Overall	4.75 [-0.36, 9.86]	5.63 [2.78, 8.48]	5.72 [-3.31, 14.75]
Injuries	Overall	-2.24 [-1.98, -2.5]	3.78 [1.52, 6.04]	3.35 [-3.31, 10.01]
Infectious	Overall	4.76 [1.01, 8.51]	4.56 [2.74, 6.38]	6.35 [1.87, 10.83]
Mental	Overall	3.51 [1.21, 5.81]	2.08 [1.13, 3.03]	4.02 [0.23, 7.81]
Neoplasms	Overall	3.33 [-0.81, 7.47]	3.32 [0.94, 5.7]	1.21 [-4.92, 7.34]
Endocrine	Overall	3.94 [1.02, 6.86]	2.35 [1.21, 3.49]	4.42 [0.26, 8.58]
Neurology	Overall	3.92 [-2.31, 10.15]	3.32 [1.23, 5.41]	2.41 [-2.95, 7.77]
Genitourinary	Overall	1.83 [-2.97, 6.63]	4.44 [1.76, 7.12]	4.43 [-2.81, 11.67]
CVD	Overall	4.21 [-0.77, 9.19]	4.98 [2.86, 7.1]	4.79 [-3.08, 12.66]

Stabilization-I period

Disease	Resilience	Storm	Flood	Blizzard/Winter storm
Respiratory	Overall	3.21 [-3.42, 9.84]	4.26 [1.42, 7.1]	3.88 [-3.12, 10.88]
Injuries	Overall	2.04 [-4.12, 8.2]	3.52 [1.36, 5.68]	2.94 [-2.41, 8.29]
Infectious	Overall	4.46 [0.67, 8.25]	3.97 [2.34, 5.6]	3.97 [0.73, 7.21]
Mental	Overall	2.78 [0.43, 5.13]	2.62 [1.72, 3.52]	3.07 [1.26, 4.88]
Neoplasms	Overall	1.63 [-4.05, 7.31]	2.98 [0.78, 5.18]	1.02 [-4.11, 6.15]
Endocrine	Overall	3.52 [0.42, 6.62]	2.31 [1.03, 3.59]	3.52 [1.42, 5.62]
Neurology	Overall	3.47 [-1.18, 8.12]	2.45 [1.01, 3.89]	1.93 [-1.46, 5.32]
Genitourinary	Overall	1.42 [-4.46, 7.3]	3.89 [1.32, 6.46]	3.44 [-2.98, 9.86]
CVD	Overall	2.33 [-4.21, 8.87]	3.78 [1.04, 6.52]	3.38 [-3.03, 9.79]

Stabilization-II period

Disease	Resilience	Storm	Flood	Blizzard/Winter storm
Respiratory	Overall	2.27 [-3.13, 7.67]	3.34 [0.28, 6.4]	-0.12 [-8.12, 7.88]
Injuries	Overall	2.44 [-2.78, 7.66]	0.69 [-1.45, 2.83]	0.51 [-5.35, 6.37]
Infectious	Overall	2.04 [-1.21, 5.29]	3.72 [1.4, 6.04]	2.44 [-1.01, 5.89]
Mental	Overall	1.93 [0.31, 3.55]	1.68 [0.86, 2.5]	2.08 [-0.51, 4.67]
Neoplasms	Overall	1.66 [-3.02, 6.34]	2.84 [0.5, 5.18]	1.21 [-4.89, 7.31]
Endocrine	Overall	1.08 [-1.43, 3.59]	1.33 [-0.03, 2.69]	1.42 [-1.01, 3.85]
Neurology	Overall	1.73 [-1.89, 5.35]	2.41 [0.33, 4.49]	1.55 [-2.01, 5.11]
Genitourinary	Overall	1.44 [-3.49, 6.37]	3.16 [0.2, 6.12]	1.81 [-5.33, 8.95]
CVD	Overall	2.74 [-2.58, 8.06]	2.34 [-0.63, 5.31]	0.92 [-6.12, 7.96]

Return to normal period

Disease	Resilience	Storm	Flood	Blizzard/Winter storm
Respiratory	Overall	0.91 [-4.41, 6.23]	0.22 [-2.76, 3.2]	2.25 [-5.87, 10.37]
Injuries	Overall	1.43 [-3.79, 6.65]	0.41 [-1.61, 2.43]	1.18 [-4.64, 7]

Infectious	Overall	1.25 [-1.69, 4.19]	-0.42 [-2.85, 2.01]	1.13 [-3.16, 5.42]
Mental	Overall	0.58 [-0.71, 1.87]	0.06 [-1.05, 1.17]	1.66 [-1.74, 5.06]
Neoplasms	Overall	1.39 [-2.89, 5.67]	-1.73 [-3.87, 0.41]	0.06 [-5.89, 6.01]
Endocrine	Overall	1.13 [-1.65, 3.91]	0.78 [-1.19, 2.75]	1.39 [-3.25, 6.03]
Neurology	Overall	0.52 [-2.41, 3.45]	0.08 [-1.63, 1.79]	2.16 [-1.54, 5.86]
Genitourinary	Overall	0.83 [-3.99, 5.65]	-0.66 [-3.91, 2.59]	0.52 [-6.77, 7.81]
CVD	Overall	1.18 [-3.9, 6.26]	0.82 [-1.84, 3.48]	0.98 [-5.92, 7.88]

Note: the western and eastern subregions are defined by the Hu Huanyong Line[83]. Tropical cyclones were not applicable for western China.

Table 17 | Relative changes of cause-specific hospitalization rates in neighboring regions (200-300 km) in western China.

Enduring period

Disease	Resilience	Storm	Flood	Blizzard/Winter storm
Respiratory	Overall	1.39 [-2.71, 5.49]	1.94 [-0.56, 4.44]	1.74 [-4.98, 8.46]
Injuries	Overall	0.24 [-3.12, 3.6]	1.33 [-0.31, 2.97]	1.67 [-3.12, 6.46]
Infectious	Overall	1.44 [-1.25, 4.13]	1.38 [-0.25, 3.01]	2.74 [-1.21, 6.69]
Mental	Overall	1.52 [-0.17, 3.21]	1.69 [0.88, 2.5]	1.43 [-1.83, 4.69]
Neoplasms	Overall	0.65 [-2.64, 3.94]	1.35 [-0.57, 3.27]	1.66 [-4.13, 7.45]
Endocrine	Overall	1.55 [-0.41, 3.51]	1.42 [0.31, 2.53]	2.97 [-0.12, 6.06]
Neurology	Overall	1.32 [-1.68, 4.32]	1.16 [-0.17, 2.49]	1.12 [-2.76, 5]
Genitourinary	Overall	0.52 [-3.31, 4.35]	0.41 [-2.01, 2.83]	0.72 [-4.99, 6.43]
CVD	Overall	2.21 [-2.14, 6.56]	2.47 [0.37, 4.57]	2.41 [-3.99, 8.81]

Stabilization-I period

Disease	Resilience	Storm	Flood	Blizzard/Winter storm
Respiratory	Overall	2.15 [-3.21, 7.51]	-0.27 [-2.68, 2.14]	1.64 [-4.21, 7.49]
Injuries	Overall	0.95 [-3.89, 5.79]	1.23 [-0.47, 2.93]	1.77 [-2.65, 6.19]
Infectious	Overall	0.98 [-3.31, 5.27]	1.62 [-0.21, 3.45]	1.96 [-0.63, 4.55]
Mental	Overall	1.89 [-0.42, 4.2]	1.34 [0.66, 2.02]	1.65 [0.48, 2.82]
Neoplasms	Overall	0.26 [-4.05, 4.57]	-0.99 [-2.55, 0.57]	-0.32 [-4.42, 3.78]
Endocrine	Overall	1.84 [-0.71, 4.39]	0.75 [-0.42, 1.92]	1.56 [-0.18, -0.16]
Neurology	Overall	-0.31 [-5.98, 5.36]	0.78 [-0.65, 2.21]	0.97 [-1.87, 3.81]
Genitourinary	Overall	0.36 [-4.71, 5.43]	0.42 [-2.11, 2.95]	-0.02 [-5.03, 4.99]
CVD	Overall	0.74 [-4.31, 5.79]	0.54 [-1.45, 2.53]	1.89 [-3.14, 6.92]

Stabilization-II period

Disease	Resilience	Storm	Flood	Blizzard/Winter storm
Respiratory	Overall	0.85 [-3.55, 5.25]	0.94 [-3.08, 4.96]	1.82 [-5.02, 8.66]
Injuries	Overall	-0.98 [-5.77, 3.81]	0.03 [-1.94, 2]	0.86 [-3.98, 5.7]
Infectious	Overall	0.79 [-2.14, 3.72]	-0.32 [-2.13, 1.49]	1.09 [-1.01, 3.19]
Mental	Overall	0.36 [-2.04, 2.76]	-0.24 [-1.32, 0.84]	0.96 [-0.06, 1.98]
Neoplasms	Overall	-0.38 [-4.67, 3.91]	0.88 [-0.92, 2.68]	0.14 [-6.11, 6.39]
Endocrine	Overall	1.64 [-0.69, 3.97]	0.63 [-82, 83.26]	0.94 [-0.89, 2.77]
Neurology	Overall	0.97 [-2.02, 3.96]	0.72 [-1.13, 2.57]	0.91 [-2, 3.82]
Genitourinary	Overall	-0.37 [-4.73, 3.99]	-0.93 [-3.02, 1.16]	-1.53 [-8.47, 5.41]
CVD	Overall	1.22 [-3.48, 5.92]	-0.39 [-2.84, 2.06]	-0.69 [-6.51, 5.13]

Return to normal period

Disease	Resilience	Storm	Flood	Blizzard/Winter storm
Respiratory	Overall	1.15 [-3.03, 5.33]	0.32 [-1.87, 2.51]	1.25 [-5.62, 8.12]
Injuries	Overall	0.51 [-2.82, 3.84]	0.73 [-1.62, 3.08]	0.21 [-6.03, 6.45]

Infectious	Overall	0.78 [-2.44, 4]	0.53 [-1.21, 2.27]	-0.56 [-4.88, 3.76]
Mental	Overall	0.45 [-1.49, 2.39]	0.82 [0.13, 1.51]	1.37 [-0.59, 3.33]
Neoplasms	Overall	1.17 [-2.36, 4.7]	0.24 [-1.63, 2.11]	0.69 [-5.72, 7.1]
Endocrine	Overall	0.14 [-3.13, 3.41]	0.15 [-1.42, 1.72]	0.99 [-0.45, 2.43]
Neurology	Overall	0.78 [-2.03, 3.59]	1.21 [-1.03, 3.45]	1.21 [-2.42, 4.84]
Genitourinary	Overall	0.32 [-3.44, 4.08]	1.32 [-1.87, 4.51]	0.05 [-6.11, 6.21]
CVD	Overall	1.13 [-2.97, 5.23]	1.13 [-1.04, 3.3]	0.42 [-5.88, 6.72]

Note: the western and eastern subregions are defined by the Hu Huanyong Line[83]. Tropical cyclones were not applicable for western China.

Table 18 | Relative changes of cause-specific hospitalization rates in neighboring regions (0-100 km) in eastern China.

Enduring period

Disease	Resilience	Storm	Flood	Blizzard/Winter storm
Respiratory	Overall	5.23 [-2.77, 13.23]	5.97 [1.73, 10.21]	7.13 [-6.64, 20.9]
Injuries	Overall	2.14 [-7.02, 11.3]	3.82 [0.44, 7.2]	3.97 [-6.31, 14.25]
Infectious	Overall	4.88 [0.02, 9.74]	4.21 [1.35, 7.07]	6.23 [-4.21, 16.67]
Mental	Overall	3.82 [0.06, 7.58]	2.14 [0.52, 3.76]	4.32 [0.06, 8.58]
Neoplasms	Overall	1.85 [-5.33, 9.03]	3.59 [0.12, 7.06]	2.11 [-7.84, 12.06]
Endocrine	Overall	3.83 [-0.94, 8.6]	2.56 [0.01, 5.11]	4.32 [-3.46, 12.1]
Neurology	Overall	3.52 [-2.78, 9.82]	3.76 [0.89, 6.63]	2.03 [-6.09, 10.15]
Genitourinary	Overall	3.01 [-5.52, 11.54]	4.24 [0.23, 8.25]	4.78 [-6.82, 16.38]
CVD	Overall	5.83 [-3.42, 15.08]	6.88 [2.64, 11.12]	5.03 [-7.31, 17.37]

Stabilization-I period

Disease	Resilience	Storm	Flood	Blizzard/Winter storm
Respiratory	Overall	3.13 [-1.42, 7.68]	6.64 [3.24, 10.04]	4.18 [-6.03, 14.39]
Injuries	Overall	1.01 [-3.48, 5.5]	4.65 [2.03, 7.27]	2.78 [-4.89, 10.45]
Infectious	Overall	4.45 [1.23, 7.67]	2.89 [0.89, 4.89]	4.78 [-0.23, 9.79]
Mental	Overall	3.31 [0.78, 5.84]	2.75 [1.47, 4.03]	4.12 [0.43, 7.81]
Neoplasms	Overall	0.24 [-3.94, 4.42]	3.31 [0.64, 5.98]	1.44 [-5.72, 8.6]
Endocrine	Overall	3.24 [0.16, 6.32]	1.32 [-0.34, 2.98]	3.89 [-2.34, 10.12]
Neurology	Overall	2.46 [-1.27, 6.19]	2.87 [0.48, 5.26]	2.19 [-2.94, 7.32]
Genitourinary	Overall	2.78 [-5.67, 11.23]	2.43 [-0.89, 5.75]	3.21 [-6.02, 12.44]
CVD	Overall	4.47 [-0.05, 8.99]	3.56 [0.13, 6.99]	3.62 [-5.5, 12.74]

Stabilization-II period

Disease	Resilience	Storm	Flood	Blizzard/Winter storm
Respiratory	Overall	3.57 [-5.07, 12.21]	2.64 [-1.74, 7.02]	1.34 [-8.87, 11.55]
Injuries	Overall	2.33 [-4.21, 8.87]	-0.53 [-4.12, 3.06]	1.93 [-5.38, 9.24]
Infectious	Overall	3.14 [-1.72, 8]	2.45 [0.23, 4.67]	2.64 [-2.78, 8.06]
Mental	Overall	2.29 [0.03, 4.55]	1.98 [0.75, 3.21]	1.53 [-2.84, 5.9]
Neoplasms	Overall	1.78 [-4.82, 8.38]	1.74 [-1.56, 5.04]	0.33 [-7.52, 8.18]
Endocrine	Overall	1.33 [-2.54, 5.2]	1.03 [-0.77, 2.83]	1.57 [-3.25, 6.39]
Neurology	Overall	2.38 [-2.46, 7.22]	1.89 [-0.25, 4.03]	1.01 [-4.42, 6.44]
Genitourinary	Overall	2.67 [-4.82, 10.16]	2.04 [-2.21, 6.29]	2.33 [-6.89, 11.55]
CVD	Overall	3.01 [-5.48, 11.5]	0.57 [-3.69, 4.83]	2.98 [-5.64, 11.6]

Return to normal period

Disease	Resilience	Storm	Flood	Blizzard/Winter storm
Respiratory	Overall	0.11 [-8.93, 9.15]	0.37 [-3.96, 4.7]	-0.56 [-10.72, 9.6]
Injuries	Overall	-1.42 [-9.35, 6.51]	-0.24 [-3.24, 2.76]	-1.03 [-8.04, 5.98]

Infectious	Overall	1.03 [-3.07, 5.13]	0.78 [-2.03, 3.59]	-0.42 [-5.83, 4.99]
Mental	Overall	0.67 [-1.98, 3.32]	-0.16 [-1.99, 1.67]	0.24 [-3.13, 3.61]
Neoplasms	Overall	-0.87 [-7.32, 5.58]	-0.93 [-5.23, 3.37]	0.13 [-9.13, 9.39]
Endocrine	Overall	0.48 [-2.24, 3.2]	1.59 [-0.63, 3.81]	0.89 [-5.34, 7.12]
Neurology	Overall	0.76 [-3.13, 4.65]	-0.37 [-3.38, 2.64]	0.33 [-5.13, 5.79]
Genitourinary	Overall	0.47 [-6.68, 7.62]	0.87 [-3.67, 5.41]	-1.34 [-10.21, 7.53]
CVD	Overall	1.53 [-7.21, 10.27]	0.65 [-3.59, 4.89]	-1.56 [-9.67, 6.55]

Note: the western and eastern subregions are defined by the Hu Huanyong Line[83]. Tropical cyclones were concentrated in eastern China, with identical DID esimates as primary analysis.

Table 19 | Relative changes of cause-specific hospitalization rates in neighboring regions (100-200 km) in eastern China.

Enduring period

Disease	Resilience	Storm	Flood	Blizzard/Winter storm
Respiratory	Overall	4.11 [-0.98, 9.2]	5.08 [2.29, 7.87]	5.36 [-3.82, 14.54]
Injuries	Overall	1.76 [-2.44, 5.96]	3.41 [1.02, 5.8]	3.07 [-3.86, 10]
Infectious	Overall	4.05 [0.52, 7.58]	3.88 [2.16, 5.6]	5.88 [1.45, 10.31]
Mental	Overall	3.03 [0.84, 5.22]	1.62 [0.67, 2.57]	3.56 [0.04, 7.08]
Neoplasms	Overall	2.89 [-1.22, 7]	2.74 [0.33, 5.15]	0.78 [-5.93, 7.49]
Endocrine	Overall	3.45 [0.57, 6.33]	2.01 [0.78, 3.24]	3.82 [0.01, 7.63]
Neurology	Overall	3.36 [0.11, 6.61]	2.76 [0.94, 4.58]	1.32 [-3.59, 6.23]
Genitourinary	Overall	1.56 [-3.32, 6.44]	3.68 [1.02, 6.34]	3.89 [-3.45, 11.23]
CVD	Overall	3.93 [-1.13, 8.99]	4.52 [1.59, 7.45]	4.24 [-3.86, 12.34]

Stabilization-I period

Disease	Resilience	Storm	Flood	Blizzard/Winter storm
Respiratory	Overall	2.64 [-4.11, 9.39]	3.72 [0.87, 6.57]	3.52 [-3.76, 10.8]
Injuries	Overall	1.56 [-4.82, 7.94]	2.89 [0.73, 5.05]	2.45 [-2.87, 7.77]
Infectious	Overall	3.88 [0.13, 7.63]	3.54 [1.86, 5.22]	3.62 [0.39, 6.85]
Mental	Overall	2.24 [0.16, 4.32]	2.21 [1.28, 3.14]	2.65 [0.87, 4.43]
Neoplasms	Overall	1.13 [-4.37, 6.63]	2.67 [0.42, 4.92]	0.64 [-5.12, 6.4]
Endocrine	Overall	2.87 [0, 5.74]	1.48 [0.39, 2.57]	2.98 [0.67, 5.29]
Neurology	Overall	3.02 [-1.18, 7.22]	2.13 [0.48, 3.78]	1.56 [-2.01, 5.13]
Genitourinary	Overall	1.04 [-5.21, 7.29]	3.33 [0.67, 5.99]	2.93 [-3.42, 9.28]
CVD	Overall	1.76 [-4.62, 8.14]	3.25 [0.72, 5.78]	2.88 [-3.57, 9.33]

Stabilization-II period

Disease	Resilience	Storm	Flood	Blizzard/Winter storm
Respiratory	Overall	1.67 [-3.93, 7.27]	2.89 [0.08, 5.7]	-0.78 [-9.21, 7.65]
Injuries	Overall	1.98 [-3.87, 7.83]	0.24 [-2.03, 2.51]	0.14 [-6.02, 6.3]
Infectious	Overall	1.45 [-2.68, 5.58]	3.13 [1.34, 4.92]	2.13 [-1.48, 5.74]
Mental	Overall	1.46 [0.02, 2.9]	1.34 [0.39, 2.29]	1.67 [-0.98, 4.32]
Neoplasms	Overall	1.32 [-3.55, 6.19]	2.32 [0.11, 4.53]	0.73 [-5.63, 7.09]
Endocrine	Overall	0.48 [-2.13, 3.09]	0.93 [-0.23, 2.09]	1.02 [-1.36, 3.4]
Neurology	Overall	1.31 [-2.38, 5]	1.67 [-0.05, 3.39]	1.03 [-2.83, 4.89]
Genitourinary	Overall	1.04 [-3.34, 5.42]	2.73 [-0.23, 5.69]	1.48 [-5.76, 8.72]
CVD	Overall	2.24 [-3.13, 7.61]	1.88 [-1.23, 4.99]	-0.34 [-7.89, 7.21]

Return to normal period

Disease	Resilience	Storm	Flood	Blizzard/Winter storm
Respiratory	Overall	0.47 [-4.86, 5.8]	-0.21 [-3.04, 2.62]	1.73 [-6.32, 9.78]
Injuries	Overall	1.21 [-4.04, 6.46]	0.34 [-1.89, 2.57]	0.88 [-5.24, 7]

Infectious	Overall	0.83 [-1.89, 3.55]	-0.92 [-3.25, 1.41]	-0.72 [-3.74, 2.3]
Mental	Overall	0.37 [-0.92, 1.66]	-0.47 [-1.87, 0.93]	1.34 [-2.03, 4.71]
Neoplasms	Overall	1.23 [-3.34, 5.8]	-2.03 [-4.42, 0.36]	-0.24 [-6.73, 6.25]
Endocrine	Overall	0.58 [-1.92, 3.08]	0.34 [-1.41, 2.09]	0.83 [-3.78, 5.44]
Neurology	Overall	0.21 [-2.78, 3.2]	-0.25 [-2.01, 1.51]	1.78 [-1.93, 5.49]
Genitourinary	Overall	0.63 [-4.92, 6.18]	-1.44 [-4.27, 1.39]	0.28 [-7.33, 7.89]
CVD	Overall	0.71 [-4.65, 6.07]	0.33 [-2.46, 3.12]	0.63 [-6.64, 7.9]

Note: the western and eastern subregions are defined by the Hu Huanyong Line[83]. Tropical cyclones were concentrated in eastern China, with identical DID esimates as primary analysis.

Table 20 | Relative changes of cause-specific hospitalization rates in neighboring regions (200-300 km) in eastern China.

Enduring period

Disease	Resilience	Storm	Flood	Blizzard/Winter storm
Respiratory	Overall	0.78 [-3.62, 5.18]	1.04 [-0.95, 3.03]	1.32 [-5.51, 8.15]
Injuries	Overall	0.04 [-3.94, 4.02]	1.09 [-0.76, 2.94]	1.17 [-4.23, 6.57]
Infectious	Overall	1.03 [-1.76, 3.82]	0.98 [-0.52, 2.48]	2.09 [-1.78, 5.96]
Mental	Overall	1.15 [-0.44, 2.74]	1.21 [0.33, 2.09]	1.16 [-2.41, 4.73]
Neoplasms	Overall	0.21 [-3.59, 4.01]	0.83 [-0.79, 2.45]	1.12 [-4.52, 6.76]
Endocrine	Overall	1.23 [-0.93, 3.39]	0.96 [0.02, 1.9]	2.24 [-0.04, 4.52]
Neurology	Overall	0.34 [-2.05, 2.73]	0.78 [-0.41, 1.97]	0.46 [-3.41, 4.33]
Genitourinary	Overall	0.32 [-3.92, 4.56]	-0.04 [-2.32, 2.24]	0.31 [-5.34, 5.96]
CVD	Overall	1.44 [-2.69, 5.57]	1.78 [0.03, 3.53]	2.03 [-4.37, 8.43]

Stabilization-I period

Disease	Resilience	Storm	Flood	Blizzard/Winter storm
Respiratory	Overall	1.73 [-3.65, 7.11]	-1.21 [-3.48, 1.06]	1.23 [-4.87, 7.33]
Injuries	Overall	0.44 [-4.24, 5.12]	0.42 [-0.89, 1.73]	1.34 [-3.42, 6.1]
Infectious	Overall	0.56 [-3.21, 4.33]	1.03 [0.02, 2.04]	1.69 [-1.03, 4.41]
Mental	Overall	1.52 [-0.73, 3.77]	1.08 [0.34, 1.82]	1.17 [0.16, 2.18]
Neoplasms	Overall	-0.21 [-4.98, 4.56]	-1.44 [-3.13, 0.25]	-0.76 [-5.13, 3.61]
Endocrine	Overall	1.34 [-1.32, 4]	0.36 [-0.87, 1.59]	1.24 [-0.47, 2.95]
Neurology	Overall	-0.58 [-6.69, 5.53]	0.24 [-1.03, 1.51]	0.56 [-2.34, 3.46]
Genitourinary	Overall	-0.05 [-5.42, 5.32]	0.13 [-2.67, 2.93]	-0.56 [-5.78, 4.66]
CVD	Overall	0.45 [-4.89, 5.79]	0.24 [-4.75, 5.23]	1.45 [-3.89, 6.79]

Stabilization-II period

Disease	Resilience	Storm	Flood	Blizzard/Winter storm
Respiratory	Overall	0.24 [-4.37, 4.85]	0.38 [-3.42, 4.18]	1.48 [-5.48, 8.44]
Injuries	Overall	-1.42 [-6.03, 3.19]	-0.32 [-2.54, 1.9]	0.67 [-4.21, 5.55]
Infectious	Overall	0.57 [-2.41, 3.55]	-0.94 [-2.69, 0.81]	0.67 [-1.92, 3.26]
Mental	Overall	0.07 [-2.32, 2.46]	-0.67 [-1.78, 0.44]	0.76 [-0.32, 1.84]
Neoplasms	Overall	-0.78 [-5.52, 3.96]	0.52 [-1.35, 2.39]	-0.23 [-6.78, 6.32]
Endocrine	Overall	1.18 [-1.03, 3.39]	0.44 [-1.21, 2.09]	0.56 [-1.38, 2.5]
Neurology	Overall	0.46 [-2.32, 3.24]	0.47 [-1.35, 2.29]	-0.67 [-3.13, 1.79]
Genitourinary	Overall	-1.13 [-5.38, 3.12]	-1.21 [-3.41, 0.99]	0.47 [-2.33, 3.27]
CVD	Overall	0.76 [-3.73, 5.25]	-1.21 [-3.37, 0.95]	-1.98 [-10.93, 6.97]

Return to normal period

Disease	Resilience	Storm	Flood	Blizzard/Winter storm
Respiratory	Overall	0.82 [-3.67, 5.31]	-0.36 [-2.62, 1.9]	0.83 [-6.42, 8.08]
Injuries	Overall	0.33 [-3.35, 4.01]	0.38 [-1.66, 2.42]	-0.02 [-6.74, 6.7]

Infectious	Overall	0.26 [-2.34, 2.86]	-0.32 [-1.73, 1.09]	-1.32 [-6.35, 3.71]
Mental	Overall	0.03 [-2.34, 2.4]	0.44 [-0.13, 1.01]	1.03 [-0.21, 2.27]
Neoplasms	Overall	0.76 [-3.11, 4.63]	-0.12 [-2.21, 1.97]	0.34 [-6.37, 7.05]
Endocrine	Overall	-0.87 [-3.72, 1.98]	-0.43 [-1.88, 1.02]	0.55 [-1.23, 2.33]
Neurology	Overall	0.45 [-2.48, 3.38]	0.74 [-0.92, 2.4]	0.77 [-2.32, 3.86]
Genitourinary	Overall	-0.27 [-4.52, 3.98]	0.79 [-2.48, 4.06]	-0.41 [-6.42, 5.6]
CVD	Overall	0.41 [-3.79, 4.61]	0.35 [-1.89, 2.59]	0.15 [-6.57, 6.87]

Note: the western and eastern subregions are defined by the Hu Huanyong Line[83]. Tropical cyclones were concentrated in eastern China, with identical DID esimates as primary analysis.

Table 21 | Summary of matched and unmatched counties using propensity score matching (PSM) for climate-related hazard analyses.

Region	Climate hazards	Matched disaster-exposed counties*	Matched control counties**	Unmatched disaster-exposed counties	Average number of matches
Disaster-exposed	Storm	232	467	0	3.1
	Flood	555	367	0	2.1
	Tropical cyclone	156	282	0	3.2
	Blizzard/Winter storm	307	480	0	2.8
N-I	Storm	50	158	0	3.5
	Flood	140	351	0	3.3
	Tropical cyclone	45	151	0	3.8
	Blizzard/Winter storm	29	115	0	3.9
N-II	Storm	193	430	0	2.6
	Flood	388	469	0	2.3
	Tropical cyclone	175	365	0	3.1
	Blizzard/Winter storm	103	286	0	3.3
N-III	Storm	311	493	0	2.9
	Flood	548	390	0	2.2
	Tropical cyclone	311	408	0	2.7
	Blizzard/Winter storm	176	410	0	3.0

Note: *The number of matched affected counties represent the administrative region where at least one Climate hazard took place in 2016 – 2023.
**The number of matched control counties were the regions which were selected as non-exposed controls at least once.

Reference

1. (PHC) SPoPHC. Health system resilience indicators: an integrated package for measuring and monitoring health system resilience in countries. Geneva: World Health Organization, 2024.
2. WHO. Measuring the climate resilience of health systems. Geneva: World Health Organization, 2022.
3. WHO. Building health system resilience to public health challenges: guidance for implementation in countries. Geneva: World Health Organization, 2024.
4. WHO. Health system resilience indicators. 2024. https://iris.who.int/bitstream/handle/10665/376071/9789240088986-eng.pdf?sequence=1.
5. Nuzzo JB, Meyer D, Snyder M, et al. What makes health systems resilient against infectious disease outbreaks and natural hazards? Results from a scoping review. *BMC Public Health* 2019; **19**(1): 1310.
6. Kruk ME, Ling EJ, Bitton A, et al. Building resilient health systems: a proposal for a resilience index. *Bmj* 2017; **357**: j2323.
7. Hanefeld J, Mayhew S, Legido-Quigley H, et al. Towards an understanding of resilience: responding to health systems shocks. *Health Policy and Planning* 2018; **33**(3): 355-67.
8. Zhong S, Clark M, Hou XY, Zang YL, Fitzgerald G. Development of hospital disaster resilience: conceptual framework and potential measurement. *Emerg Med J* 2014; **31**(11): 930-8.
9. Ebi KL, Semenza JC. Community-based adaptation to the health impacts of climate change. *Am J Prev Med* 2008; **35**(5): 501-7.
10. Romanello M, McGushin A, Di Napoli C, et al. The 2021 report of the Lancet Countdown on health and climate change: code red for a healthy future. *The Lancet* 2021; **398**(10311): 1619-62.
11. Ebi KL, Vanos J, Baldwin JW, et al. Extreme Weather and Climate Change: Population Health and Health System Implications. *Annu Rev Public Health* 2021; **42**: 293-315.
12. English PB, Sinclair AH, Ross Z, et al. Environmental Health Indicators of Climate Change for the United States: Findings from the State Environmental Health Indicator Collaborative. *Environmental Health Perspectives* 2009; **117**(11): 1673-81.
13. NBS. China Statistical Yearbook. https://data.stats.gov.cn/ (accessed 2 March 2024).
14. NHSA. National Statistical Bulletin on the Development of Healthcare Security Industry. https://www.nhsa.gov.cn/ (accessed 14th February 2024).
15. Legatum. The 2023 Legatum Prosperity Index: Legatum Institute, 2023.
16. Group WB. Distance to Frontier and Ease of Doing Business Ranking: World Bank, 2018.
17. Tee Lewis PG, Chiu WA, Nasser E, et al. Characterizing vulnerabilities to climate change across the United States. *Environ Int* 2023; **172**: 107772.
18. Institute TL. Measuring Prosperity: The Legatum Institute, 2023.
19. Greco S, Ishizaka A, Tasiou M, Torrisi G. On the Methodological Framework of Composite Indices: A Review of the Issues of Weighting, Aggregation, and Robustness. *Social Indicators Research* 2019; **141**(1): 61-94.
20. Nardo M, Saisana M, Saltelli A, Tarantola S. Tools for Composite Indicators Building; 2005.
21. UNDP. Human Development Report 2020. *UNDP (United Nations Development Programme)* 2020.
22. OECD. Handbook on Constructing Composite Indicators: Methodology and User Guide. Paris: OECD Publishing, 2008.

23. Nardo M, Saisana M, Saltelli A, Tarantola S, Hoffman A, Giovannini E. Handbook on Constructing Composite Indicators and User Guide; 2008.
24. CRED. EM-DAT The International Disaster Database. Centre for Research on the Epidemiology of Disasters.
25. EM-DAT. Minimal Requirements for a Disaster To Be Entered in EM-DAT. 2024. https://doc.emdat.be/docs/protocols/entry-criteria/.
26. EM-DAT. Know issues and limitations. 2025. https://doc.emdat.be/docs/known-issues-and-limitations/ (accessed March 25 2025).
27. IRDR. IRDR Peril Classification and Hazard Glossary: Integrated Research on Disaster Risk, 2014.
28. Rosvold EL, Buhaug H. GDIS, a global dataset of geocoded disaster locations. *Scientific Data* 2021; **8**(1): 61.
29. Observatory DF. Dartmouth Flood Observatory. In: Observatory DF, editor.; 2024.
30. Tellman B, Sullivan JA, Kuhn C, et al. Satellite imaging reveals increased proportion of population exposed to floods. *Nature* 2021; **596**(7870): 80-6.
31. EM-DAT. Encoding, quality control, and validation procedure. 2025. https://doc.emdat.be/docs/protocols/encoding-qc-and-validation/ (accessed March 25 2025).
32. Salas RN, Burke LG, Phelan J, Wellenius GA, Orav EJ, Jha AK. Impact of extreme weather events on healthcare utilization and mortality in the United States. *Nature Medicine* 2024; **30**(4): 1118-26.
33. NHSA. China Health Statical Yearbook. http://en.nhc.gov.cn/index.html (accessed 15 January 2024).
34. Bondarenko M. PR, Tejedor-Garavito N., Zhang W., McKeen T., Cunningham A., Woods T., Hilton J., Cihan D., Nosatiuk B., Brinkhoff T., Tatem A., Sorichetta A.. Estimates of 2015-2030 total number of people per grid square broken down by gender and age groupings at a resolution of 30 arc (approximately 1km at the equator) R2025A version v1. In: WorldPop - School of Geography and Environmental Science UoS, editor.: WorldPop; 2025.
35. WHO. International Statistical Classification of Diseases and Related Health Problems (ICD). https://icd.who.int/browse10/2019/en (accessed 15 January 2024).
36. Council GOotS. Notice of the General Office of the State Council on Issuing the Outline of the National Medical and Health Service System Plan (2015-2020): General Office of the State Council, 2015.
37. Beck HE, McVicar TR, Vergopolan N, et al. High-resolution (1 km) Köppen-Geiger maps for 1901–2099 based on constrained CMIP6 projections. *Scientific Data* 2023; **10**(1): 724.
38. Zhiwei L, Yinguang F, Hong S, et al. The 2022 Summer record-breaking heatwave and health information-seeking behaviours: an infodemiology study in Mainland China. *BMJ Global Health* 2023; **8**(9): e013231.
39. Bell JE, Langford BC, Kathryn C, et al. Changes in extreme events and the potential impacts on human health. *Journal of the Air & Waste Management Association* 2018; **68**(4): 265-87.
40. Vanderweele TJ. Explanation in causal inference: Methods for mediation and interaction. New York, NY, US: Oxford University Press; 2015.
41. Sun L, Abraham S. Estimating dynamic treatment effects in event studies with heterogeneous treatment effects. *Journal of Econometrics* 2021; **225**(2): 175-99.
42. Baker AC, Larcker DF, Wang CCY. How much should we trust staggered difference-in-

differences estimates? *Journal of Financial Economics* 2022; **144**(2): 370-95.

43. Parks RM, Benavides J, Anderson GB, et al. Association of Tropical Cyclones With County-Level Mortality in the US. *JAMA* 2022; **327**(10): 946-55.

44. Daw JR, Hatfield LA. Matching and Regression to the Mean in Difference-in-Differences Analysis. *Health Services Research* 2018; **53**(6): 4138-56.

45. Daw JR, Hatfield LA. Matching in Difference-in-Differences: between a Rock and a Hard Place. *Health Serv Res* 2018; **53**(6): 4111-7.

46. IPCC. Climate Change 2022: Impacts, Adaptation, and Vulnerability. Working Group II Contribution to the IPCC Sixth Assessment Report. Cambridge University Press, Cambridge, UK and New York, NY, USA, 2022.

47. GBD. Global Burden of Disease. https://www.healthdata.org/research-analysis/gbd (accessed 11 May 2024).

48. Grömping U. Variable Importance Assessment in Regression: Linear Regression versus Random Forest. *The American Statistician* 2009; **63**(4): 308-19.

49. Hastie T, Tibshirani R, Friedman J. Elements of Statistical Learning: Data Mining, Inference, and Prediction. Second Edition ed. New York: Springer; 2009.

50. Apley DW, Zhu J. Visualizing the Effects of Predictor Variables in Black Box Supervised Learning Models. *Journal of the Royal Statistical Society Series B: Statistical Methodology* 2020; **82**(4): 1059-86.

51. NBS. Classification of eastern, western, central, and northeastern region in China. 2011. https://www.stats.gov.cn/zt_18555/zthd/sjtjr/dejtjkfr/tjkp/202302/t20230216_1909741.htm.

52. Liu Z, Ma R, Wang H. Assessing urban resilience to public health disaster using the rough analytic hierarchy process method: A regional study in China. *Journal of Safety Science and Resilience* 2022; **3**(2): 93-104.

53. Jiang N, Jiang W. How does regional integration policy affect urban resilience? Evidence from urban agglomeration in China. *Environmental Impact Assessment Review* 2024; **104**: 107298.

54. Rosenback RG, Svensson A. Resilience in keeping the balance between demand and capacity in the COVID-19 pandemic, a case study at a Swedish middle-sized hospital. *BMC Health Serv Res* 2023; **23**(1): 202.

55. Cacace M, Bocken J, Edquist K, et al. Coping with COVID-19: the role of hospital care structures and capacity expansion in five countries. *Health Econ Policy Law* 2023; **18**(2): 186-203.

56. Ledesma JR, Chrysanthopoulou SA, Lurie MN, Nuzzo JB, Papanicolas I. Health system resilience during the COVID-19 pandemic: A comparative analysis of disruptions in care from 32 countries. *Health Serv Res* 2024.

57. Croghan IT, Chesak SS, Adusumalli J, et al. Stress, Resilience, and Coping of Healthcare Workers during the COVID-19 Pandemic. *J Prim Care Community Health* 2021; **12**: 21501327211008448.

58. Vainieri M, Caputo A, Vinci A. Resilience dimensions in health system performance assessments, European Union. *Bull World Health Organ* 2024; **102**(7): 498-508.

59. Tumusiime P, Karamagi H, Titi-Ofei R, et al. Building health system resilience in the context of primary health care revitalization for attainment of UHC: proceedings from the Fifth Health Sector Directors' Policy and Planning Meeting for the WHO African Region. *BMC Proc* 2020; **14**(Suppl 19): 16.

60. Burau V, Falkenbach M, Neri S, Peckham S, Wallenburg I, Kuhlmann E. Health system resilience

and health workforce capacities: Comparing health system responses during the COVID-19 pandemic in six European countries. *Int J Health Plann Manage* 2022; **37**(4): 2032-48.
61. Organization WH. A Universal Truth: No Health Without A Workforce. Geneva: World Health Organization, 2014.
62. McCauley L, Hayes R. Taking responsibility for front-line health-care workers. *Lancet Public Health* 2020; **5**(9): e461-e2.
63. Organization WH. WHO guideline on health workforce development, attraction, recruitment and retention in rural and remote areas. Geneva: World Health Organization, 2021.
64. Okoroafor SC, Ahmat A, Asamani JA, Millogo JJS, Nyoni J. An overview of health workforce education and accreditation in Africa: implications for scaling-up capacity and quality. *Hum Resour Health* 2022; **20**(1): 37.
65. Lehmann U. Strengthening human resources for health systems resilience to care for mothers and children. *BMC Health Serv Res* 2015; **15 Suppl 1**(Suppl 1): S6.
66. OECD. Health at a Glance 2021; 2021.
67. WHO. International health regulations (2005): state party self-assessment annual reporting tool. Geneva: World Health Organization, 2021.
68. WHO. Harmonized Health Facility Assessment (HHFA). World Health Organization.
69. World Health O, United Nations Children's F. Primary health care measurement framework and indicators: monitoring health systems through a primary health care lens. Web annex: technical specifications. Geneva: World Health Organization; 2022.
70. SA. The sphere handbook: humanitarian charter and minimum standards in humanitarian response. Geneva: Sphere Association, 2018.
71. Li G, Kou C, Wen F. The dynamic development process of urban resilience: From the perspective of interaction and feedback. *Cities* 2021; **114**: 103206.
72. Chen T, Radke JD, Lang W, Li X. Environment resilience and public health: Assessing healthcare's vulnerability to climate change in the San Francisco Bay Area. *Growth and Change* 2020; **51**(2): 607-25.
73. Okyere DO, Lomazzi M, Peri K, Moore M. Investing in health system resilience: A scoping review to identify strategies for enhancing preparedness and response capacity. *Population Medicine* 2024; **6**(February): 1-21.
74. Organization WH. Building health systems resilience for universal health coverage and health security during the COVID-19 pandemic and beyond: WHO position paper. Geneva: World Health Organization, 2021.
75. Wang K-L, Jiang W, Miao Z. Impact of high-speed railway on urban resilience in China: Does urban innovation matter? *Socio-Economic Planning Sciences* 2023; **87**: 101607.
76. Bareket M, Kahane Y. Achieving Environmental Resilience and Other Sustainability Goals through the Insurance and Pension System. *Harvard Business Review* 2020.
77. WHO. Global Health Observatory. Geneva: WHO.
78. Lake IR. Food-borne disease and climate change in the United Kingdom. *Environmental Health* 2017; **16**(1): 117.
79. Sathyendranath S, Abdulaziz A, Menon N, et al. Building Capacity and Resilience Against Diseases Transmitted via Water Under Climate Perturbations and Extreme Weather Stress. In: Ferretti S, ed. Space Capacity Building in the XXI Century. Cham: Springer International Publishing; 2020: 281-98.

80. Mather AE, Gilmour MW, Reid SWJ, French NP. Foodborne bacterial pathogens: genome-based approaches for enduring and emerging threats in a complex and changing world. *Nature Reviews Microbiology* 2024; **22**(9): 543-55.

81. Mu W, van Asselt ED, van der Fels-Klerx HJ. Towards a resilient food supply chain in the context of food safety. *Food Control* 2021; **125**: 107953.

82. UNICEF. Under-five mortality. United Nations Children' s Fund.

83. Association RS. The Hu Huanyong Line. 2025. https://www.regionalstudies.org/rsa-blog/hu-huanyong-line/.
